# Antihypertensive, Anti-Inflammatory, and Antiangiogenic In Silico Activity of Lactoferrin-Derived Peptides of Equine Milk Hydrolysate

**DOI:** 10.3390/biomedicines12122715

**Published:** 2024-11-27

**Authors:** Meiramkul Narmuratova, Dmitriy Berillo, Zhanar Narmuratova, Pavel Tarlykov, Assiya Serikbayeva, Shattyk Kanayat

**Affiliations:** 1 Department of Biotechnology, Faculty of Biology and Biotechnology, Al-Farabi Kazakh National University, Al-Farabi 71, Almaty 050040, Kazakhstan; m.narmuratova@gmail.com (M.N.); janarka.90b@gmail.com (Z.N.);; 2 Department of Chemical and Biochemical Engineering, Satbayev University, Satbayev 22a, Almaty 050013, Kazakhstan; 3 Department of Biochemistry, Asfendiyarov Kazakh National Medical University, Tole bi 94, Almaty 050000, Kazakhstan; 4 National Center for Biotechnology, Astana 010000, Kazakhstan; 5 Kazakh National Agrarian Research University, Abay Avenue 8, Almaty 050000, Kazakhstan

**Keywords:** equine milk, protein, lactoferrin, peptides, in silico, biological activity, 3D structure

## Abstract

Background: Equine milk, including its whey proteins, is a source of nutrients and functional components in the human diet, and is especially beneficial for people with weakened immune systems, newborns, and athletes. Objectives Whey proteins in equine milk constitute approximately 20% of the total protein content and include various fractions such as albumin, globulin, and lactoferrin. Lactoferrin is one of the most extensively studied whey proteins in equine milk. Methods: HPLC-Mass analysis, enzymatic hydrolysis, modeling of 3D structure and biological activity in silico. Results: It has antioxidant, anti-inflammatory, and immunomodulatory properties, making it a promising candidate for influencing the various aspects of cardiovascular disease pathogenesis. The products of Lactoferrin hydrolysis by trypsin were confirmed using HPLC. The half-lives of the hydrolysate in the bloodstream and in an intestine-like environment were predicted in silico. Various biological activities (antihypertensive, anti-inflammatory, and antiangiogenic) were also estimated in silico and compared with the corresponding activities of lactoferrin hydrolysate amino acid sequences from camel and dromedary milk. Conclusions: The three-dimensional modeling of lactoferrin hydrolysate peptides was performed to support the development of computational models or simulations, as well as to investigate their potential antimicrobial, anti-inflammatory, or immune-modulating functions in clinical or nutritional applications.

## 1. Introduction

According to the WHO, 1.3 billion adults are affected by hypertension worldwide (2019) [1]. The proportion of adults with hypertension is greater in low- and middle-income countries (31.5%, or 1.04 billion people) compared to high-income countries (28.5%, or 349 million people) [2]. The most commonly recommended antihypertensive medications for the first-line treatment of hypertension include (a) thiazide-type diuretics, (b) calcium channel blockers, (c) angiotensin-converting enzyme (ACE) inhibitors, and (d) angiotensin II receptor blockers (ARBs). This review summarizes the information about adverse effects, such as toxicity from thiazide and loop diuretics, which can lead to electrolyte imbalances, particularly low potassium (hypokalemia) and low sodium (hyponatremia), as well as hypochloremic metabolic acidosis. Severe dehydration is also possible, and there are no specific antidotes for these diuretics [3]. “Regulatory peptides” have reached a mature stage in research, characterized by immense complexity and diversity. Although significant progress has been made over the past 50 years, discoveries in this field continue to expand, with no indication of slowing down. Regulatory peptides remain a valuable and active area for both preclinical and clinical research [4]. In the past few decades, the problem of hypertension has become urgent. There are several research studies related not only to the development of efficient treatments for associated diseases but also to promising drugs that are in different stages of clinical trials [5,6,7]. In total, 10% of clinical trials associated with antihypertensive drugs have been terminated due to the discovery of adverse effects. The synthetic tetrazole derivative angiotensin receptor blocker Candesartan is efficient, but nevertheless, it has some side effects [8,9]. Taking into account the side effects of synthetic drugs, the search for novel, safe, and efficient treatments is of high priority. Consuming soybean peptides for eight weeks has been shown to effectively lower both mean diastolic and systolic blood pressure by inhibiting angiotensin-converting enzyme (ACE), in turn reducing the production of angiotensin II and subsequently decreasing sympathetic nervous activity, which contributes to hypertension [10]. There are some reports that dairy products may play a role in preventing and managing hypertension [11,12]. Recent research has proposed that certain peptides found in milk proteins might possess blood pressure-lowering effects [13]. An Iranian study performed on 1854 premature coronary artery disease patients revealed that in populations with low dairy intake, the moderate consumption of high-fat fermented dairy products may be associated with a lower risk of developing hypertension [14]. A clinical trial in 2019 investigated the impact of consuming whole hemp seed protein, hemp seed protein hydrolysate-derived bioactive peptides, and casein protein on systolic and diastolic ambulatory blood pressure. Participants received treatment in the form of protein powders containing either 25 g of casein protein or 25 g of hemp seed protein [15]. Peptides are extensively used as therapeutic agents in healthcare, making up over half of the pharmaceutical market, with more than 100 peptides being prescribed globally. Despite the considerable advancements in FDA-approved parenteral peptide formulations, they continue to present notable challenges [16].

According to a WHO report, in Kazakhstan, the statistics on hypertension are significantly worse compared to the global average, with 3.8 million adults aged 30–79 years affected by the condition (2023). To reach a 50% control rate for hypertension, an additional 736,000 individuals with the condition would need to receive effective treatment [17]. The solution to this problem is altering food preferences and making healthier dietary choices. The people of Kazakhstan often consume animal products; in 2023, milk and milk product (cow, camel, equine, etc.) consumption per person amounted to 227.2 kg [18]. Equine milk is an important part of the local population’s diet, as the inhabitants have used equine milk as one of the main sources of nutrition for many centuries. The supply of equine milk is very low compared to that of conventional milk types; it is less than 0.1% worldwide, but no exact statistics are available. Equine milk is consumed by approximately 30 million people worldwide (mainly in Central Asia). Equine milk is used in the non-food sector (as an ingredient in cosmetic products) and in the food sector, especially for sensitive consumers such as immunocompromised people and children allergic to cow’s milk protein [19].

Equine milk is a special product due to its high nutritional and biological value and digestibility. The milk contains more than 40 biologically active ingredients, including proteins; low-molecular-weight peptides; amino acids; vitamins A, C, B1, B2, B6, and B12; and macro- and microelements. In terms of protein composition, equine milk is close to human milk. The total amount of protein in equine milk is equal to 1.85–2.20%, and casein from equine milk, unlike cow’s milk, is easily soluble in water, which indicates good digestibility [20,21,22]. In addition, the proteins in equine milk are well balanced in terms of amino acid composition and are a source of valuable substances for humans compared to cow’s milk due to the absence of allergic reactions [23,24]. Equine milk is considered “whey milk” because it contains more whey protein than milk from other mammals. The percentage of whey proteins in equine milk is 20% higher than in cow’s milk, approximately 40%, but lower than in human milk (50%). The composition of equine milk serum proteins is as follows: β-lactoglobulin, α-lactalbumin, serum albumin, immunoglobulins, lactoferrin, and lysozyme. Among them, lactoferrin is a protein belonging to the group of transferrins found in mammals [25].

Lactoferrin (LF) is a single-polypeptide-chain glycoprotein with a molecular weight of approximately ~80 kDa. It plays an important multifunctional role in the formation of natural immunity. LF is secreted by mucosal epithelial cells of various mammals [26]. The LF concentration in equine milk is 0.2–2 g/L, which is lower than that in a mother’s milk (1–7 g/L) [27], similar to that in camel milk (0.02–2.1 g/L) [28], but higher than that in cow’s milk (0.03–0.2 g/L) above. In addition, the mentioned protein has been determined from all exocrine gland juices—tears (2 or 0.4 mg/mL), saliva (0.013 mg/mL), bronchi, bile juices (0.0042 mg/mL), pancreatitis juice, seminal fluid (0.518 mg/mL), urine (0.00005 mg/mL), and sweat (0.0025 mg/mL) [29]. 

LF is important in regulating the level of free iron in the body fluids of mammals. It has a high Fe^3+^ binding capacity (KD ≈ 10^−20^ mol L^−1^) [30] and shows significant antimicrobial activity against a variety of bacterial, viral, and fungal pathogens in laboratory tests [26,31]. LF displays its antimicrobial properties not just in its native molecular structure; both mono-ferric lobes and the active peptides of lactoferrin contribute to safeguarding host cells from microbial infections [32,33]. LF is rich in cationic and hydrophobic antimicrobial peptides that can be effective against microorganisms [34] and is enzymatically hydrolyzed by proteolytic enzymes, resulting in the formation of various functional peptides [35]. These enzymes are anticipated to exist in the gastrointestinal tract and the locations of microbial infections, possibly playing a role in lactoferrin’s natural function in the human body. This study presents an interspecies comparison of LF peptides. Numerous antimicrobial peptides have been extracted from lactoferrin and examined, but only three have received thorough investigation: LF1-11, lactoferrampin, and lactoferricin. The sequences of these peptides indicate that they belong to the N-terminal half of lactoferrin. The hydrophobicity, cationic nature, and helical conformation of these antimicrobial peptides are crucial characteristics that determine their antimicrobial potential. All of these peptides have high pI values (>9) and are anticipated to interact with negatively charged components [32].

The Food and Agriculture Organization (FAO) predicts that the world’s population will increase by one-third by 2050 [36]. As the population grows, it is predicted that the demand for protein-rich food products will increase, which will, in turn, increase the demand for milk protein. Therefore, the global milk protein market (USD 13.2 billion in 2024) may grow by an average of 7.8% over the next decade to reach approximately USD 21.7 billion at a Compound Annual Growth Rate of 5.7% by 2033 [37]. As one can see, the consumption of dairy products is huge, and the evaluation of the benefits for health is crucial. Therefore, in the current research, we focused on modeling lactoferrin enzymatic hydrolysis and evaluating the protein product degradation using HPLC–mass spectrometry. The identified peptide chains were modeled in silico to determine the 3D structure and potential biological effect of these products.

## 2. Materials and Methods

Dithiothreitol at 98%, acetonitrile HPLC at grade 99.9%, and trypsin were obtained from Thermo Scientific, Waltham, MA, USA, NUNC, Archdale, NC, USA, and trifluoroacetic acid at 99.9% was purchased from Sigma Aldrich, St. Louis, MO, USA. Trypsin at 99% was obtained from Thermo, and ZipTip-C18 was obtained from Millipore, Carrigtwohill, Ireland.

### 2.1. Lactoferrin Separation from Milk Components 

Fresh milk was sourced from a local farm to separate proteins from equine milk. The milk was precooled to 4 °C for 0.5 h before isolation. Afterward, it was transferred into 50 mL tubes and centrifuged at 5000 rpm for 30 min at 4 °C, after which the fat layer was removed. Casein was separated from other proteins by adjusting the milk’s pH to 4.2, its isoelectric point, using hydrochloric acid, followed by another centrifugation at 5000 rpm for 30 min. The resulting supernatant containing the whey proteins was neutralized to pH 6.8 with sodium hydroxide solution and dialyzed in distilled water using a dialysis membrane with a molecular weight cutoff of 6–8 kDa (SpectraPor; Spectrum Labs Inc., Rancho Dominguez, CA, USA) for 72 h at 4 °C to remove the salts. The final solution was dried under vacuum and resuspended in water to a given concentration just before analysis. The protein composition of each fraction was analyzed via 12% SDS-PAGE electrophoresis [38].

### 2.2. Protein Extraction

Each SDS-PAGE electrophoresis made of acrylamide cross-linked with an N,N^1^-methylenbisacrylamide (AAm-BisAAm) gel band was cut into 1 mm^2^ pieces. The hydrogel pieces were incubated with a mixture containing an equal amount of ammonium bicarbonate (100 mM) and acetonitrile. Reduction was performed by adding dithiothreitol (5 mM), followed by incubation at 60 °C for 10 min [39]. Afterward, the supernatant was removed, and the sample was washed three times with a 1:1 solution containing ammonium bicarbonate (50 mM) and acetonitrile for 5 min incubations at 37 °C. After the reduction was completed, the samples were digested with 20 ng/µL of trypsin at 37 °C overnight. The obtained peptide mixtures were then purified and concentrated using ZipTip-C18. The eluted peptides were dried using a centrifugal evaporator (Eppendorf, Komödie Winterhuder Fährhaus, Germany), resuspended in 10 μL of 0.1% trifluoroacetic acid, and stored at −20 °C before LC-MS/MS analysis.

### 2.3. Mass Spectrometry Analysis

The mixtures were examined using online nanoflow reversed-phase C18 liquid chromatography–tandem mass spectrometry (LC-MS/MS, Thermo). A trapping column (Acclaim PepMap 100 C18 pre-column) and a Dionex nano-HPLC pump were employed for the chromatography process. The separation of peptides took place on an Acclaim Pep-Map RSLC column (Thermo) through a 75 min multistep acetonitrile gradient, delivered at a flow rate of 0.3 mL/min. A standard captive spray ion source was employed to connect the LC system to the Impact II ESI-QUAD-TOF mass spectrometer (Bruker Bremen Dalto nics, Bremen, Germany). The setup included a capillary voltage of 1300 V, a dry gas flow rate of 3.0 L/min, and a dry temperature of 150 °C. Full-scan MS spectra were collected at a rate of 2.0 Hz, after which one MS/MS spectrum was obtained. The MS/MS peak list data were processed using DataAnalysis 3.4 software from Bruker Daltonics (Bremen, Germany) and saved in the Mascot generic format (*.mgf). These MS/MS peak lists in Mascot format were then searched on a local server with Mascot 2.6.1 software (Matrix Science, London, UK), targeting the Swiss-Prot protein database (release 2024_02), which contains 571,282 sequences and 206,678,396 residues. The search was limited to the “Other Mammalia” taxonomy, which includes 13,494 sequences. The search parameters included methionine oxidation as a variable modification and carbamidomethylation of cysteine residues as a fixed modification. For the mass spectrometry analysis, mass error windows of 100 ppm for the MS and 0.05 Da for the MS/MS were permitted [40].

### 2.4. Biological Activity of Peptides In Silico

Machine learning techniques were used for developing prediction models. Antihypertensive peptides can be checked using AHTpin software, providing various options for predicting, designing, and screening antihypertensive (AHT) peptides. It is freely available at http://crdd.osdd.net/raghava/ahtpin (accessed on 5 September 2024). We used a user-friendly web server called AntiAngioPred to predict the antiangiogenic peptides. The AntiAngioPred web server can be accessed at https://webs.iiitd.edu.in/raghava/antiangiopred/predict.html (accessed on 5 September 2024). The peptide sequence analyzer, we used is based on the PreAIP predictor database, which allowed us to determine the activity of the peptide “AIP.” The selected datasets and PreAIP are freely available at http://kurata14.bio.kyutech.ac.jp/PreAIP/ (accessed on 5 September 2024) https://webs.iiitd.edu.in/raghava/antiangiopred/predict.html (accessed on 5 September 2024). The peptides were evaluated via PLifePred analyses. Their corresponding half-lives in blood were predicted in silico using the PLifePred “Batch Submission” tool (https://webs.iiitd.edu.in/raghava/plifepred/batch.php accessed on 5 September 2024) https://webs.iiitd.edu.in/raghava/antiangiopred/predict.html (accessed on 5 September 2024).

The 3D models of the peptides generated from Lattoferrine hydrolysis were obtained using PEP-Fold and PEP-fould4, which was validated in numerous publications (https://bioserv.rpbs.univ-paris-diderot.fr/services/PEP-FOLD4/) accessed on 5 September 2024, https://webs.iiitd.edu.in/raghava/antiangiopred/predict.html (accessed on 5 September 2024). 

## 3. Results and Discussion

FitzGerald’s research group summarized data on the production of antioxidative peptides from milk proteins, the suggested mechanisms behind protein/peptide-induced antioxidant activity, methods for measuring antioxidant activity in vitro, and in vivo assessments of plasma antioxidant capacity, along with the bioavailability of these peptides. Insights from other food proteins were also referenced in cases where specific data on milk-derived peptides were lacking [41].

Previously, Gu and Wu investigated the effects of bovine lactoferrin hydrolysate digested by thermolysin, which demonstrated an IC_50_ value of 31.0 ± 1.1 µg/mL, and was unaffected by subsequent digestion with pepsin and trypsin. At a concentration of 50 µM, tripeptide Leu-Arg-Pro (LRP) significantly inhibited inflammation stimulated by tumor necrosis factor-alpha (TNF-α) in endothelial cells. These findings suggest that the ACE inhibitory tripeptide LRP not only exhibits antioxidative properties but also possesses anti-inflammatory activities, highlighting its potential for application in the management of hypertension [42].

**Figure 1:**
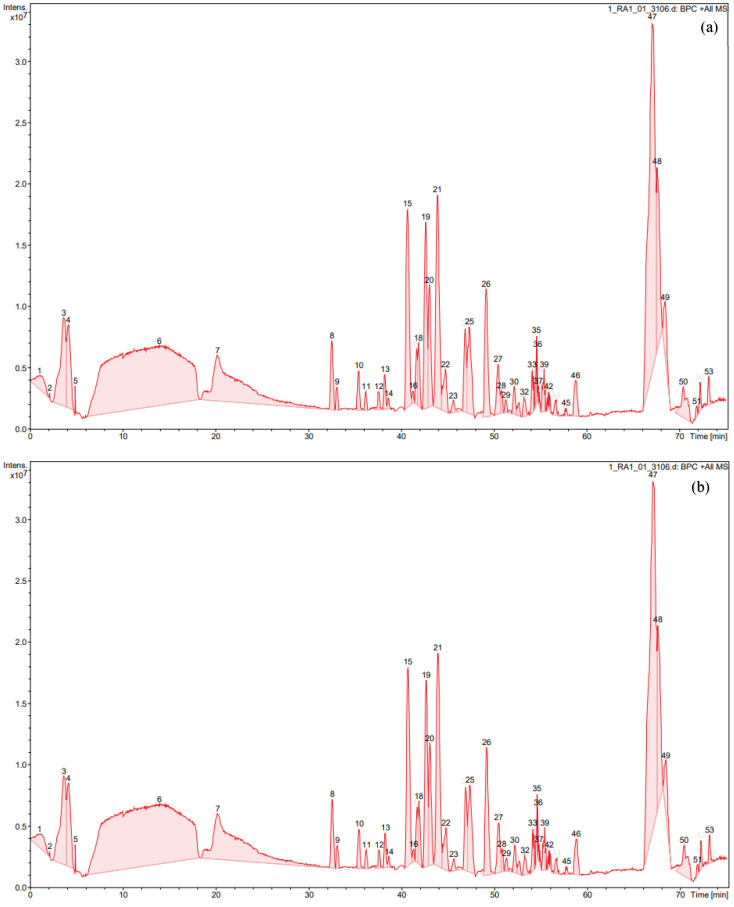
HPLC chromatogram of equine milk lactoferrin hydrolysate. (**a**) Batch 1; (**b**) batch 2.

From Table S1, and HPLC chromatograms of hydrolysate lactoferrin one can observe 52–53 peptides with different retention times, and the data on the hydrolysate content are reproducible (Figure 1).

Table 1 summarized separation of peptides by their isoelectric point, which significant constant for estimation of aggregative stability in solution. Various groups of peptides are highlighted with different colors.

It is noteworthy that the peptides exhibit a relatively short half-life of within seconds in an intestine-like environment (Table 2, Table 3 and Table 4). In contrast, their half-life in blood plasma is in the order of tens of minutes. A previous study examined the transepithelial transport of the antihypertensive hexapeptide LfcinB20–25 (RRWQWR), derived from lactoferricin B, along with its two primary fragments, RWQ and WQ, using a Caco-2 human intestinal cell monolayer. These peptides were susceptible to degradation by brush-border peptidases. While the intact LfcinB20–25 was not transported across the Caco-2 monolayer, both RWQ and WQ were absorbed. The apparent permeability (Papp) values for their absorption were 0.7 × 10^−8^ cm/s for RWQ and 3.9 × 10^−8^ cm/s for WQ [43]. Recent research indicates that the plasma concentration–time curve for radiolabeled S cell-penetrating peptides followed a two-compartment pharmacokinetic model. This model revealed a rapid distribution phase (t1/2α between 1.25 and 3 min), followed by a slower elimination phase (t1/2β between 5 and 15 h) after intravenous injection. When the S cell-penetrating peptides were combined with cargo IgG, the elimination half-life was extended to as long as 25 h. The rapid decline in the plasma concentration of the S cell-penetrating peptides was linked to their accumulation in target organs [44].

**Figure 2:**
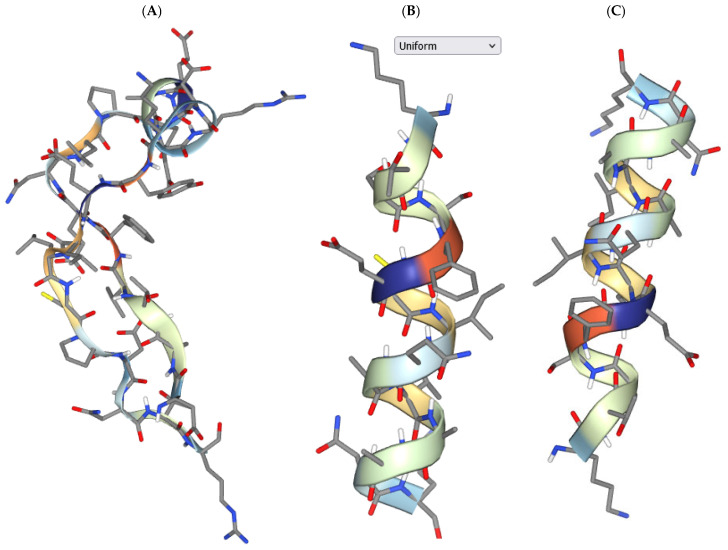
Three-dimensional structure of the peptides under conditions based on the Debye–Hückel formalism: solvent pH 7.5 and ionic strength 150 mM. (**A**) DSTVFENLPDEADRDKYELLCPDNTR; (**B**) KTSSFECIQAIA; (**C**) KTSSFECIQAIAANK.

**Figure 3:**
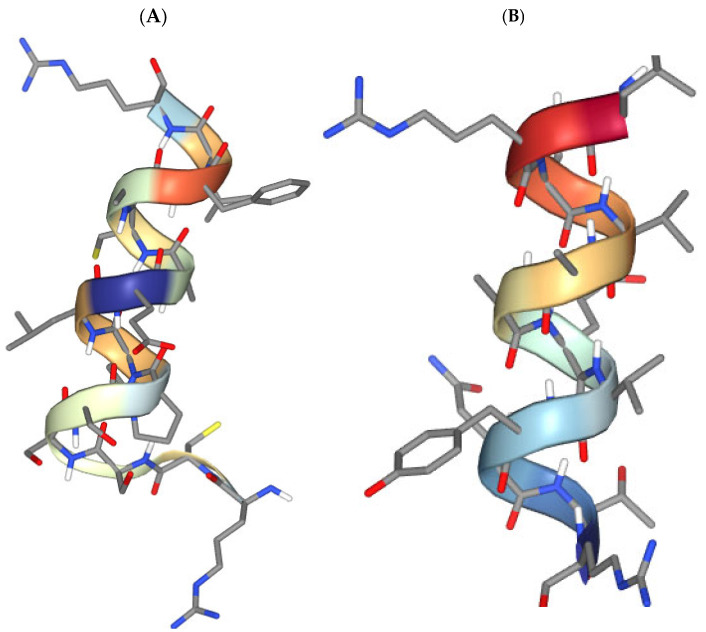
Three-dimensional structure of peptides under conditions based on Debye–Hückel formalism: solvent pH 7.5 and ionic strength 150 mM. (**A**) RCSSSPLLEACAFLR; (**B**) LRPVAAEVYQTR.

Among 13 positively charged peptides at physiological pH, only 5 amino acid sequences possessed antihypertensive activity (Table 2), while 4 of 23 negatively charged peptides had a probability of activity (Table 3). There was no correlation between the isoelectric point of the peptides and their antihypertensive activity (Table 1). It is clear that there should be a good match between the 3D structure of the peptides and the active cavity of the receptor antihypertensive peptide inhibitors. It is known that the ionic strength reaction of human blood is approximately 0.15 mol/L [45]. Therefore, we used a pH of 7.5 and an ionic strength of 0.15 mol/L for modeling 3D structures of the identified peptides (Figure 2 and Figure 3).

We provide the five most probable 3D peptide structures generated in silico (Figures S1–S5 and Tables S2–S6), which could be highly valuable for researchers across various fields. Most of the peptides exist in a-helix configuration. To better visualize the 3D structures of the peptides (DSTVFENLPDEADRDKYELLCPDNTR, KTSSFECIQAIAANK, LRPVAAEVYQTR, KTSSFECIQAIA, and RCSSSPLLEACAFLR) from various perspectives, their structure rotation can be reconstructed from the data in Tables S2–S6. The predicted local structure profile (LSP) of these peptides can be utilized for structure–function analysis. The LSP provides detailed insights into the secondary and tertiary structures at each position in the peptide chain, allowing researchers to link specific structural features with biological activities, such as antimicrobial or immune-modulating effects. These data could be particularly useful for researchers studying peptide structure–function relationships, especially those focused on lactoferrin or other milk-derived peptides from various sources (such as cows, camels, mares, or buffalos). This information may serve as a valuable reference for investigating the potential antimicrobial, anti-inflammatory, or immune-modulating functions of lactoferrin peptides in both clinical and nutritional applications. Future research will focus on evaluating the predicted activities in vivo, although this is challenging due to the complex mixture of peptides, and a synergetic effect is likely to be observed.

Casein-derived peptides have shown antihypertensive properties. Glycomacropeptide, a peptide derived from kappa casein, possesses antibacterial and antithrombotic activities. Additionally, alpha-lactalbumin has been found to exhibit antiviral, antitumor, and anti-stress effects [46].

The gastrointestinal digestion, transport, and in vivo antihypertensive effects of LVLPGE have been reported previously. The peptide showed resistance to gastrointestinal enzymes, achieving a stability of up to 98% and a permeability rate (Papp) of 5 × 10^−7^ cm/s. LVLPGE was mainly transported across the Caco-2 cell monolayer through the peptide transporter PepT1 and passive-mediated transport. LVLPGE exhibited a positive antihypertensive effect in an animal model at doses of 30 and 50 mg/kg [47]. Ten years ago, it was found that the oral bioavailability of the milk casein-derived peptide HLPLP, known for its antihypertensive effects in spontaneously hypertensive rats, was 5.18%. After oral administration, HLPLP was rapidly absorbed and eliminated, breaking down into smaller fragments, LPLP and HLPL, which were then circulated throughout the body via the bloodstream. The elimination half-lives (T1/2β) were 7.95 min for intravenous administration and 11.7 min for oral administration [48].

Of the 20 peptides with an isoelectric point in the range of 5.6–6.8, only three amino acid sequences showed activity (Table 4). Of the 13 cationic peptides, 23% and 45% showed high and medium anti-inflammatory potency in silico, respectively (Table 3).

It is interesting to note that 69.5% and 27.7% of the negatively charged amino acid sequences derived from equine milk lactoferrin showed high and medium anti-inflammatory potency, respectively (Table 4). A similar tendency was observed in the slightly negatively charged peptides from equine milk lactoferrin hydrolysate: 65% and 20% showed high and medium anti-inflammatory potency, respectively (Table 4). A decade ago, peptide-317 demonstrated significant anti-inflammatory and antinociceptive effects in mice models of colonic inflammation induced by trinitrobenzene sulfonic acid and dextran sodium sulfate. These effects, which were opioid receptor-dependent, were observed at doses of 0.1 mg/kg (intraperitoneally) and 1 mg/kg (orally). Peptide-317 also reduced the mRNA expression of proinflammatory cytokines, contributing to its anti-inflammatory action while providing pain relief in mice with acute colitis [49].

From Table 2, one can see that 8 of the 13 cationic nature peptides had a probability of possessing antiangiogenic activity, whereas only 7 of the 23 negatively charged peptides possessed this activity in silico (Table 3). An isoelectric point analysis indicates that peptides with a higher probability of activity should be positively charged. Thus, only 6 of 20 weakly acidic amino acid sequences had antiangiogenic activity (Table 4). There was no correlation between the length of the amino acid chain and the activity.

Tsuda et al. investigated the preventive effects of bovine lactoferrin and its peptide fragment, lactoferricin (bLFcin), which comprises a 25-amino-acid sequence devoid of iron-binding capacity. Their study focused on the influence of these compounds on chemically induced colon carcinogenesis in rats and on the metastasis of transplanted carcinoma cells in mice [50].

Milk-derived bioactive peptides represent a valuable source of health-promoting components with various potential health benefits. These peptides can be released during gastrointestinal digestion, food processing, or enzymatic and bacterial fermentation. They are recognized for their ability to provide a range of beneficial effects, including lipid-lowering, antihypertensive, immunomodulating, anti-inflammatory, and antithrombotic properties [51]. 

The amino acid sequence composition of dromedary lactoferrin peptic hydrolysate is different from a mare’s lactoferrin. The half-life in the blood plasma of hydrolysate is comparable to equine milk lactoferrin. Of the twelve dromedary lactoferrin peptic hydrolysates, only four (IDKVAHL, RIDKVAHL, GSPAGQKDLL, and QLFGSPAGQKDL) demonstrated antihypertensive activity (Table 5).

Five peptides of different lengths (WAKDLKL, GSPAGQKDLL, GRRRSVQWCAV, WNLLRQAQEKFGKDKSP, and KCFQWQRNMRKVRGPPVSCIKRDS) revealed antiangiogenic activity in silico (Table 5). DVTVLDNTDGK and VKDVTVLDNTDGKNTEQW, with a common amino acid sequence derived from camel LF, did not possess antihypertensive or antiangiogenic activities. CTTSPAESSKCAQW had the probability of antihypertensive activity. Peptides with numbers 14, 15, and 16 from Table 5 can be considered as potentially having antiangiogenic activity.

Recently, 24 out of 63 lactoferrin-derived peptides from camel LF hydrolysate were found to be cationic, which is a characteristic feature of antimicrobial peptides. The LFFPALLSLGALGLCLAASK peptide demonstrated strong antimicrobial activity against a resistant strain of *Escherichia coli* at low concentrations while exhibiting relatively low toxicity to human fibroblast cells [55]. Additionally, the peptide showed high bioavailability and excellent digestibility. Peptides can be utilized for specific medical purposes, such as supporting immunity or treating allergies. Cow’s milk can be a more affordable option for the mass production of peptides, but its peptides may have some restrictions in terms of digestibility and specific activity [23]. Cow’s milk is actively used in functional foods and additives, and its peptides also have useful properties. Mare’s milk and its composition are distinguished by the best conditions for processing and use for medical and dietary purposes. In mare’s milk, lactoferrin can have other properties due to its unique composition. Lactoferrin made from mare’s milk has higher bioavailability and can be easier than for an organism to assimilate [53]. This makes it more effective in some specific therapeutic areas (e.g., for the immune system).

Human LF1-11 (GRRRSVQWCAV) features a highly variable loop region and a short β-strand with a high concentration of arginine. This arginine-rich portion resembles other naturally occurring cationic arginine-rich peptides known for their ability to penetrate cells, enabling them to cross the plasma membrane of eukaryotic cells [32].

The enzymatic absorption of lactoferrin occurs in the context of antimicrobial peptides that exhibit antimicrobial properties, sometimes proving to be more effective than native LF. This could play a crucial role in protecting neonates from invading pathogens. These peptides, derived from the N-lobe of lactoferrin, show a remarkable similarity to cationic antimicrobial peptides present in various invertebrate and vertebrate species [32]. LF preserves these peptides in a manner that is largely consistent across most species. While there may be slight variations in the structure and characteristics of these lactoferrin peptides among different species [53], the core mechanisms remain largely similar and intact. This indicates that these peptides significantly contribute to the antimicrobial function of the protein. The antimicrobial effect of cationic peptides from various sources is also linked to their ability to disrupt the cytoplasmic membrane of target cells.

## 4. Conclusions

Lactoferrin peptides with antihypertensive properties are promising biologically active substances for controlling and reducing blood pressure. Their ability to inhibit ACE, improve vascular tone, and protect cells from oxidative stress makes them interesting for use in medicine and the food industry as a preventive and therapeutic agent against hypertension. In this study, of the investigated cationic peptides, 34.8% exhibited antihypertensive activity in silico, while 17.4% of the 23 anionic peptides and 15% of the 20 slightly anionic peptides showed potential for similar activity. Of the 13 cationic peptides analyzed, 23% and 45% demonstrated high and medium anti-inflammatory potency, respectively, when compared to some published in vivo studies on bovine lactoferrin derivatives. Additionally, 61% of the 13 positively charged peptides were predicted to possess antiangiogenic activity, in contrast to only 34.4% of the 23 negatively charged peptides. An isoelectric point analysis suggested that positively charged peptides have a higher likelihood of exhibiting activity, as 30% of the 20 weakly negative amino acid sequences also displayed antiangiogenic activity. It is interesting to note that the hydrolysis of lactoferrin from different sources (camel, dromedary, equine, etc.) leads to variations in amino acid sequences, generating extensive data. The predicted biological potency of these sequences should also be evaluated in vitro through solid-phase peptide synthesis and subsequent in vitro and in vivo studies. A more complex task is to predict the structure–activity relationships and synergistic effects associated with the consumption of such lactoferrin hydrolysates. 

We created models of the five most likely 3D peptide structures under physiological conditions, which could be highly valuable for researchers across various fields, such as scientists studying peptide structure–function relationships, especially those focusing on lactoferrin or other milk-derived peptides. Future research will focus on evaluating projected activities in vivo, which is difficult due to the complex mixture of peptides, and a synergetic effect will likely be observed. This 3D peptide model may further explain the interaction with the active site of the appropriate receptor.

## Figures and Tables

**Table 1:** Amino acid sequence and isoelectric point of hydrolysate equine milk lactoferrin peptides.

Amino Acid Sequence	Amino Acid Sequence	Isoelectric Point pI/Mw:
Cationic peptides		
KGSGFQLNQLQGVK	Lys-Gly-Ser-Gly-Phe-Gln-Leu-Asn-Gln-Leu-Gln-Val-Lys	10.00/1502.82
VPSHAVVAR	Val-Pro-Ser-His-Ala-Val-Val-Ala-Arg	9.73/934.53
GSGFQLNQLQGVK	Gly-Ser-Gly-Phe-Gln-Leu-Asn-Gln-Leu-Gln-Gly-Val-Lys	8.75/1374.73
LRPVAAEVYQTR	Leu-Arg-Pro-Val-Ala-Ala-Glu-Val-Tyr-Glu-Thr-Arg	8.75/1401.77
YYAVAVVK	Tyr-Tyr-Ala-Val-Ala-Val-Val-Lys	8.50/911.51
KSDADLTWNSLSGKK	Lys-Ser-Asp-Ala-Asp-Leu-Thr-Trp-Asn-Ser-Leu-Ser-Gly-Lys-Lys	8.50/1648.84
YYGYTGAFR	Tyr-Tyr-Gly-Tyr-Thr-Gly-Ala-Phe-Arg	8.50/1096.50
SSAFQLFK	Ser-Ser-Ala-Phe-Gln-Leu-Phe-Lys	8.47/926.49
GPSVSCIR	Gly-Pro-Ser-Val-Ser-Cys-Ile-Arg	8.25/817.41
QYPNLCR	Gln-Tyr-Pro-Asn-Leu-Cys-Arg	8.22/892.42
FCLFK	Phe-Cys-Leu-Phe-Lys	8.22/656.34
KTSSFECIQAIAANK	Lys-Thr-Ser-Ser-Phe-Glu-Cys-Ile-Gln-Ala-Ile-Ala-Ala-Asn-Lys	8.20/1609.81
RCSSSPLLEACAFLR	Arg-Cys-Ser-Ser-Ser-Pro-Leu-Leu-Glu-Ala-Cys-Ala-Phe-Lys-Arg	8.07/1651.82
Neutral		
APNHAVVSQSDR	Ala-Pro-Asn-His-Ala-Val-Val-Ser-Gln-Ser-Asp-Arg	6.79/1279.63
KPVAEAESCHLAR	Lys-Pro-Val-Ala-Glu-Ala-Glu-Ser-Cys-His-Leu-Ala-Arg	6.75/1409.71
SQNSNAPDCVHRPPEGYLAVAVVR	Ser-Gln-Asn-Ser-Asn-Ala-Pro-Asp-Cys-Val-His-Arg-Pro-Pro-Glu-Gly-Tyr-Leu-Ala-Val-Ala-Val-Val-Arg	6.47/2578.27
KVACASASTTEECIALVLK	Lys-Val-Ala-Cys-Ala-Ser-Ala-Ser-Thr-Thr-Glu-Glu-Cys-Ile-Ala-Leu-Val-Leu-Lys	6.13/1936.00
NSEPWAK	Asn-Ser-Glu-Pro-Trp-Ala-Lys	6.00/830.39
WCTISPAEAAK	Trp-Cys-Thr-Ile-Ser-Pro-Ala-Glu-Ala-Ala-Lys	5.99/1175.56
CSSSPLLEACAFLR	Cys-Ser-Ser-Ser-Pro-Leu-Leu-Glu-Ala-Cys-Ala-Phe-Leu-Arg	5.99/1495.72
CGLVPVLAENQK	Cys-Gly-Leu-Val-Pro-Val-Leu-Ala-Glu-Asn-Gln-Lys	5.99/1269.68
CACSSQEPYFGYSGAFK	Cys-Ala-Cys-Ser-Ser-Gln-Glu-Pro-Tyr-Ser-Gly-Ala-Phe-Lys	5.99/1843.75
DLLFKDSALGFVR	Asp-Leu-Leu-Phe-Lys-Asp-Ser-Ala-Leu-Gly-Phe-Val-Arg	5.96/1479.81
KSDADLTWNSLSGK	Lys-Ser-Asp-Ala-Asp-Leu-Thr-Trp-Asn-Ser-Leu-Ser-Gly-Lys	5.96/1520.75
KVLFLQQDQFGGNGPDCPGK	Lys-Val-Leu-Phe-Leu-Gln-Gln-Asp-Gln-Phe-Gly-Gly-Asn-Gly-Pro-Asp-Cys-Pro-Gly-Lys	5.95/2147.05
AGDVAFVK	Ala-Gly-Asp-Val-Ala-Phe-Val-Lys	5.88/805.43
AVANFFSASCVPCADGK	Ala-Val-Ala-Asn-Phe-Phe-Ser-Ala-Ser-Cys-Val-Pro-Cys-Ala-Asp-Gly-Lys	5.86/1685.75
QWSDVSNR	Gln-Trp-Ser-Asp-Val-Ser-Asn-Arg	5.84/990.45
DSALGFVR	Asp-Ser-Ala-Leu-Gly-Phe-Val-Arg	5.84/863.45
IPSQIDSGLYLGANYLTATQNLR	Ile-Pro-Ser-Gln-Ile-Asp-Ser-Gly-Leu-Tyr-Leu-Gly-Ala-Asn-Tyr-Leu-Thr-Ala-Thr-Gln-Asn-Leu-Arg	5.83/2507.30
TTYEQYLGSEYVTSITNLRR	Thr-Thr-Tyr-Glu-Gln-Tyr-Leu-Gly-Ser-Glu-Tyr-Val-Thr-Ser-Ile-Thr-Asn-Leu-Arg-Arg	5.81/2393.19
STPENKDLLFK	Ser-Thr-Pro-Glu-Asn-Lys-Asp-Leu-Leu-Phe-Lys	5.79/1290.68
TSSFECIQAIAANK	Thr-Ser-Ser-Phe-Glu-Cys-Ile-Gln-Ala-Ile-Ala-Ala-Asn-Lys	5.66/1481.72
Anionic peptides		
VVWCAVGPEEERK	Val-Val-Trp-Cys-Ala-Val-Gly-Pro-Glu-Glu-Glu-Arg-Lys	4.79/1500.74
LCAGTEADKCACSSQEPYFGYSGAFK	Leu-Cys-Ala-Gly-Thr-Glu-Ala-Asp-Lys-Cys-Ala-Cys-Ser-Ser-Gln-Glu-Pro-Tyr-Phe-Gly-Tyr-Ser-Gly-Ala-Phe-Lys	4.68/2732.16
SVDGREDLIWR	Ser-Val-Asp-Gly-Arg-Glu-Asp-Leu-Ile-Trp-Arg	4.56/1344.68
ADAVTLDGGLVYEAGLHPYK	Ala-Asp-Ala-Val-Thr-Leu-Asp-Gly-Gly-Leu-Val-Tyr-Glu-Ala-Gly-Leu-His-Pro-Tyr-Lys	4.54/2088.05
ETAAEVAAR	Glu-Thr-Ala-Ala-Glu-Val-Ala-Ala-Arg	4.53/916.46
VACASASTTEECIALVLK	Val-Ala-Cys-Ala-Ser-Ala-Ser-Thr-Thr-Glu-Glu-Cys-Ile-Ala-Leu-Val-Leu-Lys	4.53/1807.91
TTYEQYLGSEYVTSITNLR	Thr-Thr-Tyr-Glu-Gln-Tyr-Leu-Gly-Ser-Glu-Tyr-Val-Thr-Ser-Ile-Thr-Asn-Leu-Arg	4.53/2237.09
CLENGAGDVAFVK	Cys-Leu-Glu-Asn-Gly-Ala-Gly-Asp-Val-Ala-Phe-Val-Lys	4.37/1321.63
YELLCPDNTR	Tyr-Glu-Leu-Leu-Cys-Pro-Asp-Asn-Thr-Arg	4.37/1222.57
EDLIWR	Glu-Asp-Leu-Ile-Trp-Arg	4.37/830.43
VVWCAVGPEEER	Val-Val-Trp-Cys-Ala-Val-Gly-Pro-Glu-Glu-Glu-Arg	4.25/1372.64
DVTVLQNTDGK	Asp-Val-Thr-Val-Leu-Gln-Asn-Thr-Asp-Gly-Lys	4.21/1188.60
SDADLTWNSLSGK	Ser-Asp-Ala-Asp-Leu-Thr-Trp-Asn-Ser-Leu-Ser-Gly-Lys	4.21/1392.65
VLFLQQDQFGGNGPDCPGK	Val-Leu-Phe-Leu-Gln-Gln-Asp-Gln-Phe-Gly-Gly-Asn-Gly-Pro-Asp-Cys-Pro-Gly-Lys	4.21/2018.95
VACASASTTEECIALVLKGEADALNLDGGFIYVAGK	Val-Ala-Cys-Ala-Ser-Ala-Ser-Thr-Thr-Glu-Glu-Cys-Ile-Ala-Leu-Val-Leu-Lys-Gly-Glu-Ala-Asp-Ala-Leu-Asp-Gly-Gly-Phe-Ile-Tyr-Val-Ala-Gly-Lys	4.18/3598.79
NLLFNDNTECLAELQGK	Asn-Leu-Leu-Phe-Asn-Asp-Asn-Thr-Glu-Cys-Leu-Ala-Glu-Leu-Gln-Gly-Lys	4.14/1920.93
FFSQSCAPGADPQSSLCALCVGNNENENK	Phe-Phe-Ser-Gln-Ser-Cys-Ala-Pro-Gly-Ala-Asp-Pro-Gln-Ser-Ser-Leu-Cys-Ala-Leu-Cys-Val-Gly-Asn-Asn-Glu-Asn-Glu-Asn-Lys	4.14/3029.30
DLKQEDFELLCLDGTR	Asp-Leu-Lys-Gln-Glu-Asp-Phe-Glu-Leu-Leu-Cys-Leu-Asp-Gly-Thr-Arg	4.11/1893.91
GEADALNLDGGFIYVAGK	Gly-Glu-Ala-Asp-Ala-Leu-Asn-Leu-Asp-Gly-Gly-Phe-Ile-Tyr-Val-Ala-Gly-Lys	4.03/1808.89
DSTVFENLPDEADRDKYELLCPDNTR	Asp-Ser-Thr-Val-Phe-Glu-Asn-Leu-Pro-Asp-Glu-Ala-Asp-Arg-Asp-Lys-Tyr-Glu-Leu-Leu-Cys-Pro-Asp-Asn-Thr-Arg	4.03/3054.39
DSTVFENLPDEADRDK	Asp-Ser-Thr-Val-Phe-Glu-Asn-Leu-Pro-Asp-Glu-Ala-Asp-Arg-Lys	3.96/1849.83
QEDFELLCLDGTR	Gln-Glu-Asp-Phe-Glu-Leu-Leu-Cys-Leu-Asp-Gly-Thr-Arg	3.92/1537.71
DSTVFENLPDEADR	Asp-Ser-Thr-Val-Phe-Glu-Asn-Leu-Pro-Asp-Glu-Ala-Asp-Arg	3.77/1606.71

**Table 2:** Predicted biological activity of equine milk lactoferrin hydrolysate peptides with isoelectric point in the range of 8.1–10.0.

#	Amino Acid Sequence	Half-Life in Blood Plasma (in Seconds)	Half-Life in Intestine-like Environment (in Seconds)	AHTpin		PreAIP (Anti-Inflammatory Potency)		AntiAngio-Pred	
				Score	Prediction	Score	Confidence Label	Score	Prediction
1	GPSVSCIR	771	1.19	−1.12	Non-AHT	0.41	Medium	1.09	Anti-AAP
2	KTSSFECIQAIAANK	832	0.92	−0.99	Non-AHT	0.39	Medium	−0.11	Non-anti-AAP
3	LRPVAAEVYQTR	866	0.70	−0.30	Non-AHT	0.51	High	−0.88	Non-anti-AAP
4	YYAVAVVK	834	0.95	0.39	AHT	0.41	Medium	0.14	Anti-AAP
5	KGSGFQLNQLQGVK	900	3.74	−1.02	Non-AHT	0.44	Medium	0.50	Anti-AAP
6	GSGFQLNQLQGVK	855	3.8	−0.79	Non-AHT	0.48	High	0.25	Anti-AAP
7	QYPNLCR	698	1.74	0.49	AHT	0.39	Medium	2.88	Anti-AAP
8	VPSHAVVAR	828	1.10	−0.12	Non-AHT	0.38	Low	−0.89	Non-anti-AAP
9	SSAFQLFK	841	1.35	−0.93	Non-AHT	0.33	Negative	−0.84	Non-anti-AAP
10	KSDADLTWNSLSGKK	936	1.37	−1.43	Non-AHT	0.37	Low	−0.46	Non-anti-AAP
11	YYGYTGAFR	788	1.47	0.29	AHT	0.46	Medium	0.20	Anti-AAP
12	FCLFK	834	1.24	0.11	AHT	0.38	Low	0.79	Anti-AAP
13	RCSSSPLLEACAFLR	723	0.90	0.53	AHT	0.47	High	1.81	Anti-AAP

**Table 3:** Predicted biological activity of hydrolysate equine milk lactoferrin peptides with isoelectric point in the range of 4.8–3.7.

#	Amino Acid Sequence	Half-Life in Blood Plasma (Seconds)	Half-Life in Intestine-like Environment (Seconds)	AHTpin		PreAIP (Anti-Inflammatory Potency)		AntiAngio-Pred	
				Score	Prediction	Score	Confidence Label	Score	Prediction
1.	ADAVTLDGGLVYEAGLHPYK	884	0.548	−1.22	Non-AHT	0.446	Medium	−2.28	Non-anti-AAP
2.	LCAGTEADKCACSSQEPYFGYSGAFK	2390	3.04	−0.33	Non-AHT	0.58	High	0.20	Anti-AAP
3.	CLENGAGDVAFVK	589	2.714	−1.80	Non-AHT	0.59	High	1.81	Non-anti-AAP
4.	DSTVFENLPDEADR	763	3.376	−0.91	Non-AHT	0.45	Medium	−0.49	Non-anti-AAP
5.	DSTVFENLPDEADRDK	795	0.268	−0.93	Non-AHT	0.53	High	−0.86	Non-anti-AAP
6.	DSTVFENLPDEADRDKYELLCPDNTR	669	0.385	−1.10	Non-AHT	0.56	High	0.04	Anti-AAP
7.	YELLCPDNTR	719	1.187	0.47	AHT	0.57	High	1.44	Anti-AAP
8.	SVDGREDLIWR	775	0.731	−1.57	Non-AHT	0.56	High	0.10	Anti-AAP
9.	EDLIWR	914	1.407	−0.75	Non-AHT	0.40	Medium	−0.56	Non-anti-AAP
10.	ETAAEVAAR	904	1.188	−0.73	Non-AHT	0.35	Low	−0.46	Non-anti-AAP
11.	VVWCAVGPEEER	821	2.281	0.15	AHT	0.60	High	0.12	Anti-AAP
12.	VVWCAVGPEEERK	812	2.442	−0.01	Non-AHT	0.57	High	−0.04	Non-anti-AAP
13.	VACASASTTEECIALVLK	863	0.679	−1.17	Non-AHT	0.55	High	0.01	Anti-AAP
14.	VACASASTTEECIALVLKGEADALNLDGGFIYVAGK	843	1.048	−2.09	Non-AHT	0.47	High	−1.56	Non-anti-AAP
15.	GEADALNLDGGFIYVAGK	1044	1.483	−2.04	Non-AHT	0.47	High	−2.73	Non-anti-AAP
16.	SDADLTWNSLSGK	661	1.347	−0.77	Non-AHT	0.46	Medium	−0.21	Non-anti-AAP
17.	FFSQSCAPGADPQSSLCALCVGNNENENK	683	1.936	−0.29	Non-AHT	0.57	High	0.20	Anti-AAP
18.	DVTVLQNTDGK	762	1.009	−1.49	Non-AHT	0.36	Low	−0.80	Non-anti-AAP
19.	DLKQEDFELLCLDGTR	802	0.8	−0.52	Non-AHT	0.54	High	−0.28	Non-anti-AAP
20.	QEDFELLCLDGTR	854	1.678	−0.23	Non-AHT	0.54	High	−0.12	Non-anti-AAP
21.	VLFLQQDQFGGNGPDCPGK	956	3.601	0.05	AHT	0.44	Medium	−0.32	Non-anti-AAP
22.	NLLFNDNTECLAELQGK	851	0.418	−1.00	Non-AHT	0.55	High	−0.73	Non-anti-AAP
23.	TTYEQYLGSEYVTSITNLR	694	0.403	0.74	AHT	0.53	High	−0.11	Non-anti-AAP

**Table 4:** Predicted biological activity of hydrolysate equine milk lactoferrin peptides with isoelectric point in the range of 5.6–6.8.

#	Amino Acid Sequence	Half-Life in Blood Plasma (in Seconds)	Half-Life in Intestine-like Environment (in Seconds)	Ahtpin		PreAIP (Anti-Inflammatory Potency)		AntiAngio-Pred	
				Score	Prediction	Score	Confidence Label	Score	Prediction
1.	WCTISPAEAAK	865	1.304	−0.57	Non-AHT	0.53	High	0.92	Anti-AAP
2.	TSSFECIQAIAANK	800	0.996	−0.95	Non-AHT	0.51	High	−0.07	Non-anti-AAP
3.	AVANFFSASCVPCADGK	734	0.356	−1.23	Non-AHT	0.55	High	−0.55	Non-anti-AAP
4.	CACSSQEPYFGYSGAFK	210	2.279	0.60	AHT	0.59	High	0.63	Anti-AAP
5.	STPENKDLLFK	988	1.701	−1.20	Non-AHT	0.46	Medium	0.21	Anti-AAP
6.	DLLFKDSALGFVR	555	4.246	−1.64	Non-AHT	0.50	High	−0.74	Non-anti-AAP
7.	DSALGFVR	440	3.828	−1.47	Non-AHT	0.43	Medium	−0.79	Non-anti-AAP
8.	IPSQIDSGLYLGANYLTATQNLR	375	2.446	−0.83	Non-AHT	0.52	High	−0.28	Non-anti-AAP
9.	QWSDVSNR	904	1.748	−1.94	Non-AHT	0.35	Low	0.52	Anti-AAP
10.	KVACASASTTEECIALVLK	806	1.095	−1.24	Non-AHT	0.57	High	−0.11	Non-anti-AAP
11.	CGLVPVLAENQK	738	2.278	−0.93	Non-AHT	0.45	Medium	−0.27	Non-anti-AAP
12.	SQNSNAPDCVHRPPEGYLAVAVVR	686	1.134	−0.57	Non-AHT	0.48	High	−0.71	Non-anti-AAP
13.	KSDADLTWNSLSGK	778	1.124	−1.21	Non-AHT	0.48	High	−0.45	Non-anti-AAP
14.	AGDVAFVK	799	3.238	−0.17	Non-AHT	0.36	Low	−1.58	Non-anti-AAP
15.	NSEPWAK	813.81	1.373	−0.65	Non-AHT	0.31	negative AIP	−0.13	Non-anti-AAP
16.	KPVAEAESCHLAR	915	3.021	−0.54	Non-AHT	0.47	High	−0.48	Non-anti-AAP
17.	APNHAVVSQSDR	811	1.262	−1.35	Non-AHT	0.49	High	−0.95	Non-anti-AAP
18.	KVLFLQQDQFGGNGPDCPGK	1098	1.272	−0.09	Non-AHT	0.45	Medium	−0.10	Non-anti-AAP
19.	TTYEQYLGSEYVTSITNLRR	819	0.977	0.62	AHT	0.55	High	0.32	Anti-AAP
20.	CSSSPLLEACAFLR	748	0.87	0.60	AHT	0.57	High	1.45	Anti-AAP

**Table 5:** Predicted biological activity for dromedary and camel lactoferrin peptic hydrolysates based on peptide amino acids sequences published previously.

#	Amino Acid Sequence	Half-Life in Blood Plasma (in Seconds)	Half-Life in Intestine-like Environment (in Seconds)	AHTpin		AntiAngio-Pred		
				Score	Prediction	Score	Prediction	
1	QLFGSPAGQKDL	1256	2.11	0.24	AHT	−0.15	Non-anti-AAP	[52]
2	GSPAGQKDLL	1637	1.66	0.56	AHT	0.45	Anti-AAP	
3	FKDSALGL	596	0.59	−0.44	Non-AHT	−1.15	Non-anti-AAP	
4	VLKGEADAL	877	0.59	−0.97	Non-AHT	−1.48	Non-anti-AAP	
5	LDCVHRPVKGY	772	0.59	−0.20	Non-AHT	−0.50	Non-anti-AAP	
6	WAKDLKL	839	0.59	−0.29	Non-AHT	0.70	Anti-AAP	
7	RIDKVAHL	761	0.594	0.36	AHT	−1.54	Non-anti-AAP	
8	FKDSALGL	596	-	−0.44	Non-AHT	−1.15	Non-anti-AAP	
9	IDKVAHL	813	-	0.34	AHT	−1.38	Non-anti-AAP	
10	GRRRSVQWCAV	862	1.455	−1.54	Non-AHT	0.23	Anti-AAP	[53]
11	WNLLRQAQEKFGKDKSP	0.243	-	−1.07	Non-AHT	0.41	Anti-AAP	
12	KCFQWQRNMRKVRGPPVSCIKRDS	1325	-	−0.92	Non-AHT	1.48	Anti-AAP	
	camel lactoferrin signature peptide							
13	DVTVLDNTDGK	815	1.228	−1.17	Non-AHT	−0.44	Non-anti-AAP	[54]
14	FPALLSLGALGLCLAASKKSVRW	798	0.036	−0.96	Non-AHT	0.68	Anti-AAP	[55]
15	CTTSPAESSKCAQW	855	2.171	1.54	AHT	1.80	Anti-AAP	[55]
16	QRRMKKVRGPSVTCVKKTSRF	876	-	−0.52	Non-AHT	0.33	Anti-AAP	[55]
17	VKDVTVLDNTDGKNTEQW	790	-	−0.84	Non-AHT	−1.05	Non-anti-AAP	[55]

## Data Availability

Data are contained within the article and Supplementary Materials.

## Supplementary Materials

The following supporting information can be downloaded at: https://www.mdpi.com/article/10.3390/biomedicines12122715/s1, Figure S1: HPLC data for 3 batches of Equine Milk Lactoferrin hydrolysate; Table S1. 3D structure of peptide DSTVFENLPDEADRDKYELLCPDNTR and predicted Local Structure Profile of the peptide. pep-fold4.py--maxSimSze 50--maxSeqSze 50--noMQA--sortKey sOPEP-s iSeq.fasta-l PEPFOLD--generator fbt--nRuns 100--mcSteps 30000--mcT 370--seed 1 Debye-Huckel formalism Solvent pH 7.5 Ionic strength (mM) 150; Table S2. DSTVFENLPDEADRDKYELLCPDNTR PEPFOLD-5bestmodels.pdb; Figure S2. 3D structure of peptide and predicted Local Structure Profile of KTSSFECIQAIA job execution pep-fold2.py--autoFromTo--parallelposttreatment--maxSimSze 40-s iSeq.fasta-l PEPFOLD--shortcombined--sortKey sOPEP; Table S3. Model 1 (*Protein* KTSSFECIQAIA *_3DStructure*); Figure S3. 3D structure of peptide and Predicted Local Structure Profile KTSSFECIQAIAANK job execution pep-fold4.py--maxSimSze 50--maxSeqSze 50--noMQA--sortKey sOPEP-s iSeq.fasta-l PEPFOLD--generator fbt--nRuns 100--mcSteps 30000--mcT 370--seed 1--user_pKa pKa.data Solvent pH 7.5, Ionic strength (150mM); Table S4. KTSSFECIQAIAANK PEPFOLD-5 bestmodels.pdb; Figure S4. 3D structure of peptide and predicted local structure profile RCSSSPLLEACAFLR job execution pep-fold4.py--maxSimSze 50--maxSeqSze 50--noMQA--sortKey sOPEP-s iSeq.fasta-l PEPFOLD--generator fbt--nRuns 100--mcSteps 30000--mcT 370--seed 1 Debye-Huckel formalism Solvent pH 7.5 Ionic strength (mM) 150; Table S5. 5 best Models *(Protein_3DStructure)* RCSSSPLLEACAFLR; Figure S5. 3D structure of LRPVAAEVYQTR and Predicted Local Structure Profile job execution pep-fold4.py--maxSimSze 50--maxSeqSze 50--noMQA--sortKey sOPEP-s iSeq.fasta-l PEPFOLD--generator fbt--nRuns 100--mcSteps 30000--mcT 370--seed 1 Debye-Huckel formalism Solvent pH7.5 Ionic strength (150 mM); Table S6. LRPVAAEVYQTR PEPFOLD-5 bestmodels.pdb.
