# Supplementary material for: Antihypertensive, Anti-Inflammatory, and Antiangiogenic In Silico Activity of Lactoferrin-Derived Peptides of Equine Milk Hydrolysate
Source: Biomedicines. 2024 Nov 27;12(12):2715. doi: 10.3390/biomedicines12122715 (PMC11726958; doi:10.3390/biomedicines12122715)
Supplement: Supplementary file 1 [file biomedicines-12-02715-s001.zip › biomedicines-3265010-supplementary.pdf]

Supplementary Information

Antihypertensive, Anti-Inflammatory and Antiangiogenic Activity of Lactoferrin-Derived Peptides of Equine Milk Hydrolysate In Silico

Meiramkul Narmuratova[1,ʃ*], Dmitriy Berillo[ʃ,2,3*], Zhanar Narmuratova[1,2], Pavel Tarlykov[4], Assiya Serikbayeva[5], Shattyk Kanayat[1]

[1] *Department of Biotechnology, Faculty of Biology and Biotechnology, Al-Farabi Kazakh National University, Al-Farabi 71, 050040, Almaty, Kazakhstan*

[2] *Department of Chemical and Biochemical Engineering, Satbayev University, Satbayev 22a, 050013 Almaty, Kazakhstan*

[3] *Department of Pharmaceutical and Toxicological Chemistry, School of Pharmacy, Asfendiyarov Kazakh National Medical University, Tole bi 94, Almaty 050000, Kazakhstan*

[4] *National Center for Biotechnology, Astana, 010000, Kazakhstan*

[5] *Kazakh National Agrarian Research University, Abay Avenue 8, 050000, Almaty, Kazakhstan*

* ***E-mail****:* m.narmuratova@gmail.com, d.berillo@satbayev.university

ʃ Authors contributed equally

Table S1 HPLC data for 3 batches of Equine Milk Lactoferrin hydrolysate

Com-pound #	Retention Time (RT) [min]	relative content, %	RT [min]	relative content, %	RT [min]	relative content, %
1			0.7	0.81	0.5	0.57
2	1	0.91	1.5	9.63	1.2	0.24
3	2.1	0.03	20.3	1.67	11	0.23
4	3.6	3.44	30.8	0.09	14.1	1.18
5	4.1	2.09	31.3	0.16	15.1	0.05
6	4.9	0.07	31.7	1.02	17.5	1.19

7	13.9	31.06	33.1	11.55	19.9	1.05
8	20.2	8.47	33.7	1.74	20.1	0.31
9	32.5	1.13	34.3	0.08	20.5	0.04
10	33.1	0.29	34.9	0.59	20.7	0.07
11	35.4	0.57	35.6	1.99	30.1	1.74
12	36.2	0.24	36	3.27	30.6	0.17
13	37.6	0.25	37.1	0.20	31.3	0.18
14	38.2	0.50	37.8	0.56	31.7	0.64
15	38.6	0.12	38.1	1.21	32.2	1.03
16	40.7	4.03	38.5	0.40	32.7	2.54
17	41.3	0.16	39.3	1.68	33.4	6.83
18	41.7	0.62	41	9.94	34.6	1.99
19	41.9	0.83	41.6	0.38	35.1	1.39
20	42.6	3.33	42	1.62	35.8	0.24
21	43	2.07	43.1	0.17	37.2	4.10
22	43.9	4.87	43.6	1.09	37.6	1.62
23	44.8	1.02	44	0.21	38.3	6.52
24	45.6	0.23	45.2	0.71	39	9.00
25	46.9	1.35	45.7	4.40	39.4	6.14
26	47.3	2.15	46.7	0.28	39.8	0.05
27	49.1	2.64	47	1.07	40.2	2.67

28	50.4	0.98	47.8	2.55	40.8	1.34
29	50.8	0.26	48.7	0.35	41.4	0.72
30	51.3	0.18	49.4	2.55	42.2	0.14
31	52.2	0.42	50.4	2.66	42.6	1.50
32	52.7	0.22	53	1.96	42.9	0.63
33	53.2	0.34	59	0.24	43.1	1.04
34	54.1	0.41	62.2	25.66	44.2	0.34
35	54.3	0.15	63.3	3.15	44.7	9.30
36	54.6	0.48	64.1	0.13	45.6	0.71
37	54.7	0.15	65	0.89	45.9	1.61
38	54.8	0.08			46.5	0.29
39	54.9	0.10			47.2	2.79
40	55.4	0.52			47.6	0.46
41	55.5	0.08			48.1	0.08
42	55.7	0.06			49	0.36
43	55.9	0.08			58.4	1.24
44	56	0.10			60.2	19.93
45	56.7	0.24			61.3	2.90
46	57.8	0.10			62.2	0.10
47	58.8	0.76	66	0.16	63.1	0.66
48	67.1	15.20	67.9	1.22	64	0.12

49	67.6	3.46	69.7	0.43	65.8	1.11
50	68.4	1.23	70.1	0.32	67.7	0.47
51	70.4	1.19	70.8	0.68	69.8	0.32
52	71.9	0.12			70.3	0.08
53	72.2	0.28	72	0.52		
54	73.1	0.34				

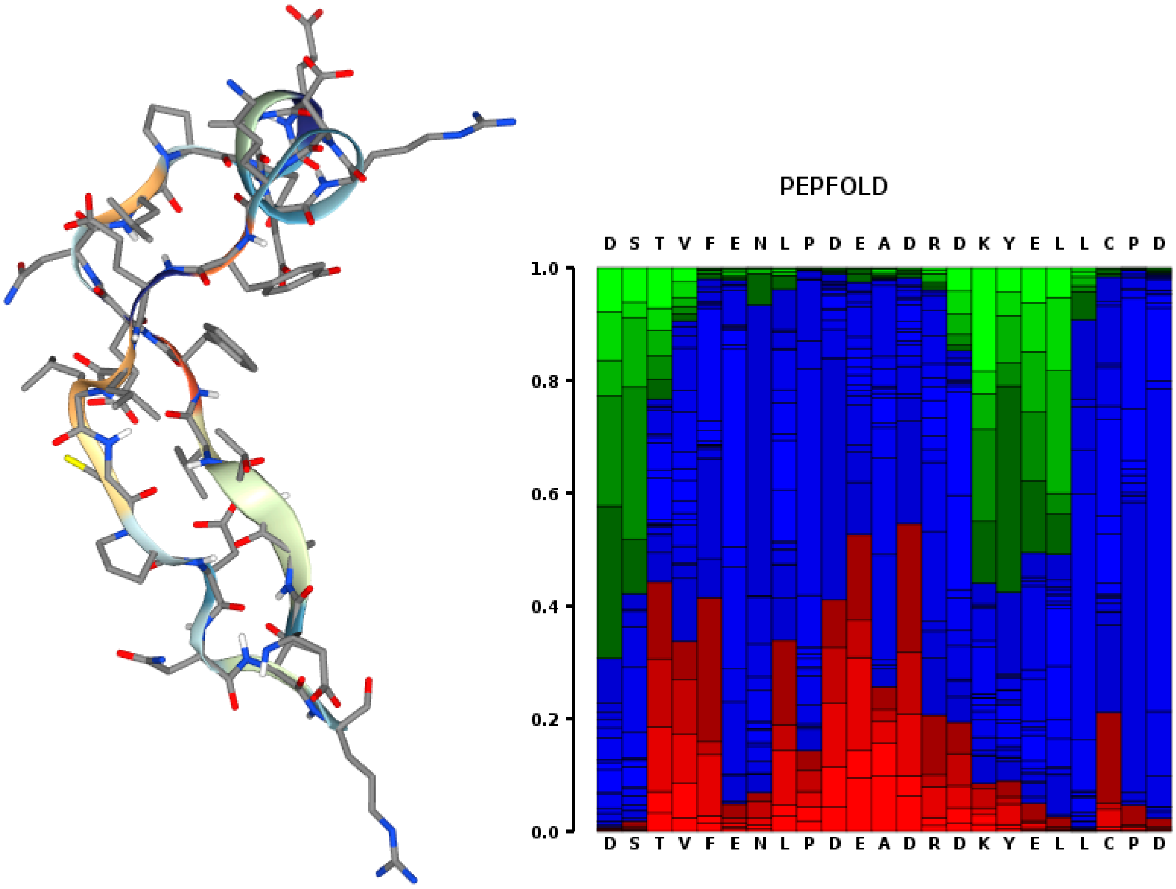

Figure S1 3D structure of peptide DSTVFENLPDEADRDKYELLCPDNTR and predicted Local Structure Profile of the peptide. pep-fold4.py --maxSimSze 50 --maxSeqSze 50 --noMQA --sortKey sOPEP -s iSeq.fasta -l PEPFOLD --generator fbt --nRuns 100 --mcSteps 30000 --mcT 370 --seed 1 Debye-Huckel formalism Solvent pH 7.5 Ionic strength (mM) 150

Table S2 DSTVFENLPDEADRDKYELLCPDNTR PEPFOLD-5bestmodels.pdb

```
MODEL     1
ATOM    0 N   ASP A  1    122.700  10.787 216.085
ATOM    1 CA  ASP A  1    124.085  10.355 215.910
ATOM    2 C   ASP A  1    124.439   9.522 214.677
ATOM    3 O   ASP A  1    125.555   9.627 214.164
ATOM    4 H   ASP A  1    122.439  11.344 216.899
ATOM    5 CB  ASP A  1    124.462   9.517 217.146
ATOM    6 CG  ASP A  1    124.484  10.354 218.415
ATOM    7 OD1 ASP A  1    124.808  11.549 218.364
ATOM    8 OD2 ASP A  1    124.178   9.840 219.502
ATOM    0 N   SER A  2    123.517   8.701 214.188
ATOM    1 CA  SER A  2    123.834   7.893 213.015
ATOM    2 C   SER A  2    123.011   8.204 211.775
ATOM    3 O   SER A  2    121.938   8.805 211.842
ATOM    4 H   SER A  2    122.636   8.638 214.607
ATOM    5 CB  SER A  2    123.608   6.414 213.377
ATOM    6 OG  SER A  2    122.206   6.217 213.578
ATOM    0 N   THR A  3    123.543   7.785 210.633
ATOM    1 CA  THR A  3    122.894   7.982 209.352
ATOM    2 C   THR A  3    121.965   6.803 209.112
ATOM    3 O   THR A  3    122.282   5.674 209.480
ATOM    4 H   THR A  3    124.405   7.326 210.656
ATOM    5 CB  THR A  3    123.950   8.050 208.234
ATOM    6 OG1 THR A  3    124.821   9.164 208.451
ATOM    7 CG2 THR A  3    123.317   8.210 206.861
ATOM    0 N   VAL A  4    120.797   7.048 208.499
ATOM    1 CA  VAL A  4    119.889   5.927 208.253
ATOM    2 C   VAL A  4    119.818   6.013 206.703
ATOM    3 O   VAL A  4    119.869   7.046 206.042
ATOM    4 H   VAL A  4    120.557   7.954 208.222
ATOM    5 CB  VAL A  4    118.512   6.142 208.907
ATOM    6 CG1 VAL A  4    117.485   5.135 208.415
ATOM    7 CG2 VAL A  4    118.574   6.015 210.420
ATOM    0 N   PHE A  5    119.753   4.796 206.174
ATOM    1 CA  PHE A  5    119.512   4.561 204.727
ATOM    2 C   PHE A  5    118.198   3.755 204.501
ATOM    3 O   PHE A  5    117.708   3.025 205.372
ATOM    4 H   PHE A  5    119.868   4.023 206.761
ATOM    5 CB  PHE A  5    120.694   3.767 204.141
ATOM    6 CG  PHE A  5    122.001   4.537 204.237
ATOM    7 CD1 PHE A  5    122.718   4.525 205.404
ATOM    8 CE1 PHE A  5    123.896   5.219 205.492
ATOM    9 CZ  PHE A  5    124.356   5.926 204.412
ATOM   10 CE2 PHE A  5    123.639   5.938 203.245
ATOM   11 CD2 PHE A  5    122.461   5.244 203.158
ATOM    0 N   GLU A  6    117.631   3.977 203.327
ATOM    1 CA  GLU A  6    116.412   3.330 202.905
ATOM    2 C   GLU A  6    116.039   3.624 201.451
ATOM    3 O   GLU A  6    116.665   4.492 200.816
ATOM    4 H   GLU A  6    118.058   4.608 202.715
```

ATOM 5 CB GLU A 6 115.263 3.813 203.810
ATOM 6 CG GLU A 6 115.460 3.389 205.256
ATOM 7 CD GLU A 6 114.352 3.907 206.157
ATOM 8 OE1 GLU A 6 114.304 3.556 207.346
ATOM 9 OE2 GLU A 6 113.495 4.680 205.704
ATOM 0 N ASN A 7 115.128 2.823 200.906
ATOM 1 CA ASN A 7 114.832 2.835 199.485
ATOM 2 C ASN A 7 116.242 2.645 198.694
ATOM 3 O ASN A 7 116.485 3.331 197.697
ATOM 4 H ASN A 7 114.639 2.204 201.483
ATOM 5 CB ASN A 7 114.185 4.176 199.091
ATOM 6 CG ASN A 7 112.752 4.284 199.585
ATOM 7 OD1 ASN A 7 111.980 3.321 199.463
ATOM 8 ND2 ASN A 7 112.450 5.470 200.130
ATOM 0 N LEU A 8 117.070 1.717 199.149
ATOM 1 CA LEU A 8 118.228 1.308 198.331
ATOM 2 C LEU A 8 118.324 -0.102 198.568
ATOM 3 O LEU A 8 118.362 -0.346 199.780
ATOM 4 H LEU A 8 116.915 1.305 200.021
ATOM 5 CB LEU A 8 119.509 2.029 198.789
ATOM 6 CG LEU A 8 119.407 3.536 198.616
ATOM 7 CD1 LEU A 8 120.647 4.253 199.124
ATOM 8 CD2 LEU A 8 119.226 3.931 197.160
ATOM 0 N PRO A 9 118.354 -1.059 197.647
ATOM 1 CA PRO A 9 118.450 -2.473 198.019
ATOM 2 C PRO A 9 119.860 -2.700 198.721
ATOM 3 O PRO A 9 120.762 -1.857 198.756
ATOM 4 CB PRO A 9 118.335 -3.294 196.721
ATOM 5 CG PRO A 9 117.610 -2.348 195.746
ATOM 6 CD PRO A 9 118.075 -0.959 196.194
ATOM 0 N ASP A 10 119.851 -3.785 199.484
ATOM 1 CA ASP A 10 120.919 -4.070 200.426
ATOM 2 C ASP A 10 122.257 -4.282 199.821
ATOM 3 O ASP A 10 123.251 -4.060 200.516
ATOM 4 H ASP A 10 119.104 -4.411 199.411
ATOM 5 CB ASP A 10 120.543 -5.346 201.203
ATOM 6 CG ASP A 10 119.343 -5.127 202.110
ATOM 7 OD1 ASP A 10 119.152 -4.016 202.627
ATOM 8 OD2 ASP A 10 118.556 -6.058 202.333
ATOM 0 N GLU A 11 122.306 -4.573 198.517
ATOM 1 CA GLU A 11 123.598 -4.819 197.837
ATOM 2 C GLU A 11 124.080 -3.379 197.561
ATOM 3 O GLU A 11 125.276 -3.122 197.717
ATOM 4 H GLU A 11 121.476 -4.623 198.004
ATOM 5 CB GLU A 11 123.405 -5.606 196.528
ATOM 6 CG GLU A 11 122.987 -7.045 196.785
ATOM 7 CD GLU A 11 122.750 -7.808 195.492
ATOM 8 OE1 GLU A 11 122.511 -9.025 195.526
ATOM 9 OE2 GLU A 11 122.794 -7.215 194.404
ATOM 0 N ALA A 12 123.199 -2.462 197.166
ATOM 1 CA ALA A 12 123.641 -1.102 196.874

ATOM 2 C ALA A 12 124.097 -0.432 198.206
ATOM 3 O ALA A 12 125.108 0.273 198.154
ATOM 4 H ALA A 12 122.255 -2.699 197.070
ATOM 5 CB ALA A 12 122.479 -0.297 196.262
ATOM 0 N ASP A 13 123.418 -0.623 199.336
ATOM 1 CA ASP A 13 123.849 0.032 200.567
ATOM 2 C ASP A 13 125.305 -0.390 200.817
ATOM 3 O ASP A 13 126.084 0.451 201.270
ATOM 4 H ASP A 13 122.632 -1.203 199.342
ATOM 5 CB ASP A 13 122.962 -0.414 201.744
ATOM 6 CG ASP A 13 123.253 0.377 203.009
ATOM 7 OD1 ASP A 13 124.212 1.162 203.042
ATOM 8 OD2 ASP A 13 122.531 0.238 204.008
ATOM 0 N ARG A 14 125.691 -1.621 200.507
ATOM 1 CA ARG A 14 127.075 -2.030 200.691
ATOM 2 C ARG A 14 128.082 -1.155 199.776
ATOM 3 O ARG A 14 129.063 -0.527 200.167
ATOM 4 H ARG A 14 125.042 -2.258 200.152
ATOM 5 CB ARG A 14 127.214 -3.514 200.306
ATOM 6 CG ARG A 14 128.610 -4.047 200.585
ATOM 7 CD ARG A 14 128.617 -5.555 200.775
ATOM 8 NE ARG A 14 129.945 -6.055 201.161
ATOM 9 CZ ARG A 14 130.133 -7.375 201.371
ATOM 10 NH1 ARG A 14 131.343 -7.831 201.723
ATOM 11 NH2 ARG A 14 129.111 -8.229 201.229
ATOM 0 N ASP A 15 127.691 -1.093 198.524
ATOM 1 CA ASP A 15 128.511 -0.358 197.596
ATOM 2 C ASP A 15 128.728 1.139 197.699
ATOM 3 O ASP A 15 129.818 1.703 197.601
ATOM 4 H ASP A 15 126.869 -1.532 198.230
ATOM 5 CB ASP A 15 127.904 -0.603 196.202
ATOM 6 CG ASP A 15 127.990 -2.064 195.792
ATOM 7 OD1 ASP A 15 128.941 -2.761 196.177
ATOM 8 OD2 ASP A 15 127.110 -2.560 195.074
ATOM 0 N LYS A 16 127.598 1.792 197.951
ATOM 1 CA LYS A 16 127.577 3.244 198.054
ATOM 2 C LYS A 16 126.180 3.450 198.875
ATOM 3 O LYS A 16 125.156 2.840 198.558
ATOM 4 H LYS A 16 126.768 1.290 198.070
ATOM 5 CB LYS A 16 127.528 3.916 196.669
ATOM 6 CG LYS A 16 128.869 3.849 195.956
ATOM 7 CD LYS A 16 128.828 4.533 194.600
ATOM 8 CE LYS A 16 130.169 4.466 193.887
ATOM 9 NZ LYS A 16 130.128 5.132 192.566
ATOM 0 N TYR A 17 126.220 4.310 199.890
ATOM 1 CA TYR A 17 125.044 4.881 200.502
ATOM 2 C TYR A 17 124.636 6.280 200.176
ATOM 3 O TYR A 17 125.524 7.094 200.507
ATOM 4 H TYR A 17 127.094 4.571 200.241
ATOM 5 CB TYR A 17 125.262 4.827 202.025
ATOM 6 CG TYR A 17 125.273 3.400 202.549

ATOM	7	CD1	TYR	A	17	126.417	2.651	202.472
ATOM	8	CE1	TYR	A	17	126.427	1.365	202.944
ATOM	9	CZ	TYR	A	17	125.292	0.828	203.492
ATOM	10	CE2	TYR	A	17	124.148	1.577	203.569
ATOM	11	CD2	TYR	A	17	124.138	2.863	203.097
ATOM	12	OH	TYR	A	17	125.302	-0.468	203.968
ATOM	0	N	GLU	A	18	123.423	6.647	199.759
ATOM	1	CA	GLU	A	18	123.099	8.070	199.805
ATOM	2	C	GLU	A	18	122.178	8.288	201.040
ATOM	3	O	GLU	A	18	121.406	7.461	201.516
ATOM	4	H	GLU	A	18	122.775	5.990	199.437
ATOM	5	CB	GLU	A	18	122.363	8.488	198.518
ATOM	6	CG	GLU	A	18	123.263	8.408	197.296
ATOM	7	CD	GLU	A	18	122.524	8.774	196.019
ATOM	8	OE1	GLU	A	18	123.143	8.866	194.949
ATOM	9	OE2	GLU	A	18	121.301	8.980	196.048
ATOM	0	N	LEU	A	19	122.397	9.488	201.568
ATOM	1	CA	LEU	A	19	121.567	10.045	202.667
ATOM	2	C	LEU	A	19	120.107	9.824	202.494
ATOM	3	O	LEU	A	19	119.658	10.251	201.432
ATOM	4	H	LEU	A	19	123.134	10.027	201.219
ATOM	5	CB	LEU	A	19	121.820	11.562	202.746
ATOM	6	CG	LEU	A	19	123.279	11.883	203.025
ATOM	7	CD1	LEU	A	19	123.578	13.364	202.862
ATOM	8	CD2	LEU	A	19	123.687	11.497	204.438
ATOM	0	N	LEU	A	20	119.340	9.453	203.507
ATOM	1	CA	LEU	A	20	117.887	9.507	203.444
ATOM	2	C	LEU	A	20	117.633	9.913	204.816
ATOM	3	O	LEU	A	20	116.701	10.743	204.910
ATOM	4	H	LEU	A	20	119.764	9.131	204.327
ATOM	5	CB	LEU	A	20	117.265	8.136	203.120
ATOM	6	CG	LEU	A	20	115.988	8.268	202.306
ATOM	7	CD1	LEU	A	20	115.042	7.101	202.538
ATOM	8	CD2	LEU	A	20	115.224	9.537	202.648
ATOM	0	N	CYS	A	21	118.240	9.430	205.900
ATOM	1	CA	CYS	A	21	117.818	9.937	207.219
ATOM	2	C	CYS	A	21	118.528	10.040	208.459
ATOM	3	O	CYS	A	21	119.694	9.674	208.328
ATOM	4	H	CYS	A	21	118.946	8.759	205.823
ATOM	5	CB	CYS	A	21	116.590	9.077	207.570
ATOM	6	SG	CYS	A	21	115.259	9.094	206.329
ATOM	0	N	PRO	A	22	118.061	10.469	209.613
ATOM	1	CA	PRO	A	22	118.942	10.514	210.762
ATOM	2	C	PRO	A	22	118.417	9.470	211.609
ATOM	3	O	PRO	A	22	117.202	9.616	211.743
ATOM	4	CB	PRO	A	22	118.830	11.907	211.409
ATOM	5	CG	PRO	A	22	118.368	12.816	210.254
ATOM	6	CD	PRO	A	22	117.528	11.878	209.383
ATOM	0	N	ASP	A	23	119.132	8.637	212.356
ATOM	1	CA	ASP	A	23	118.501	7.693	213.269
ATOM	2	C	ASP	A	23	119.240	8.601	214.473

```
ATOM      3  O   ASP A  23      120.445   8.778 214.716
ATOM      4  H   ASP A  23      120.107   8.657 212.293
ATOM      5  CB  ASP A  23      118.990   6.237 213.152
ATOM      6  CG  ASP A  23      118.305   5.496 212.016
ATOM      7  OD1 ASP A  23      117.136   5.776 211.708
ATOM      8  OD2 ASP A  23      118.914   4.611 211.396
ATOM      0  N   ASN A  24      118.294   9.329 215.047
ATOM      1  CA  ASN A  24      118.687  10.489 215.836
ATOM      2  C   ASN A  24      118.902   9.828 217.377
ATOM      3  O   ASN A  24      118.998  10.312 218.505
ATOM      4  H   ASN A  24      117.352   9.093 214.944
ATOM      5  CB  ASN A  24      117.581  11.560 215.841
ATOM      6  CG  ASN A  24      117.806  12.616 214.770
ATOM      7  OD1 ASN A  24      116.861  12.989 214.059
ATOM      8  ND2 ASN A  24      119.069  13.059 214.699
ATOM      0  N   THR A  25      119.126   8.543 217.130
ATOM      1  CA  THR A  25      119.368   7.611 218.222
ATOM      2  C   THR A  25      120.600   7.970 219.077
ATOM      3  O   THR A  25      121.566   8.532 218.554
ATOM      4  H   THR A  25      119.128   8.218 216.208
ATOM      5  CB  THR A  25      119.583   6.207 217.628
ATOM      6  OG1 THR A  25      118.428   5.810 216.883
ATOM      7  CG2 THR A  25      119.827   5.163 218.706
ATOM      0  N   ARG A  26      120.550   7.664 220.372
ATOM      1  CA  ARG A  26      121.672   7.927 221.272
ATOM      2  C   ARG A  26      122.839   6.987 220.944
ATOM      3  O   ARG A  26      122.703   6.077 220.122
ATOM      4  H   ARG A  26      119.741   7.252 220.732
ATOM      5  CB  ARG A  26      121.226   7.695 222.728
ATOM      6  CG  ARG A  26      122.320   8.048 223.722
ATOM      7  CD  ARG A  26      121.756   8.388 225.092
ATOM      8  NE  ARG A  26      122.801   8.844 226.020
ATOM      9  CZ  ARG A  26      122.478   9.188 227.285
ATOM     10  NH1 ARG A  26      123.430   9.603 228.131
ATOM     11  NH2 ARG A  26      121.204   9.115 227.694
TER
ENDMDL
MODEL        2
ATOM      0  N   ASP A   1      123.043  14.325 211.546
ATOM      1  CA  ASP A   1      124.208  13.766 212.228
ATOM      2  C   ASP A   1      124.510  12.277 212.052
ATOM      3  O   ASP A   1      125.678  11.881 212.066
ATOM      4  H   ASP A   1      122.820  15.316 211.647
ATOM      5  CB  ASP A   1      124.017  14.014 213.736
ATOM      6  CG  ASP A   1      124.054  15.495 214.078
ATOM      7  OD1 ASP A   1      124.762  16.267 213.415
ATOM      8  OD2 ASP A   1      123.375  15.931 215.020
ATOM      0  N   SER A   2      123.489  11.443 211.886
ATOM      1  CA  SER A   2      123.755  10.018 211.720
ATOM      2  C   SER A   2      123.360   9.440 210.371
ATOM      3  O   SER A   2      122.561  10.012 209.629
```

ATOM	4	H	SER	A	2	122.572	11.780	211.873
ATOM	5	CB	SER	A	2	122.977	9.257	212.810
ATOM	6	OG	SER	A	2	121.580	9.396	212.534
ATOM	0	N	THR	A	3	123.946	8.289	210.065
ATOM	1	CA	THR	A	3	123.681	7.585	208.826
ATOM	2	C	THR	A	3	122.483	6.677	209.049
ATOM	3	O	THR	A	3	122.319	6.114	210.130
ATOM	4	H	THR	A	3	124.580	7.901	210.699
ATOM	5	CB	THR	A	3	124.907	6.742	208.430
ATOM	6	OG1	THR	A	3	126.035	7.593	208.209
ATOM	7	CG2	THR	A	3	124.664	5.946	207.158
ATOM	0	N	VAL	A	4	121.618	6.528	208.034
ATOM	1	CA	VAL	A	4	120.460	5.655	208.226
ATOM	2	C	VAL	A	4	120.413	5.057	206.810
ATOM	3	O	VAL	A	4	120.835	5.679	205.824
ATOM	4	H	VAL	A	4	121.759	6.992	207.186
ATOM	5	CB	VAL	A	4	119.183	6.444	208.569
ATOM	6	CG1	VAL	A	4	117.972	5.535	208.706
ATOM	7	CG2	VAL	A	4	119.319	7.208	209.876
ATOM	0	N	PHE	A	5	119.872	3.847	206.734
ATOM	1	CA	PHE	A	5	119.664	3.166	205.467
ATOM	2	C	PHE	A	5	118.294	2.768	205.342
ATOM	3	O	PHE	A	5	117.664	2.307	206.291
ATOM	4	H	PHE	A	5	119.603	3.398	207.559
ATOM	5	CB	PHE	A	5	120.564	1.918	205.404
ATOM	6	CG	PHE	A	5	120.543	1.270	204.029
ATOM	7	CD1	PHE	A	5	121.424	1.682	203.064
ATOM	8	CE1	PHE	A	5	121.405	1.098	201.825
ATOM	9	CZ	PHE	A	5	120.505	0.102	201.551
ATOM	10	CE2	PHE	A	5	119.625	-0.311	202.516
ATOM	11	CD2	PHE	A	5	119.644	0.273	203.755
ATOM	0	N	GLU	A	6	117.780	2.952	204.131
ATOM	1	CA	GLU	A	6	116.454	2.415	203.731
ATOM	2	C	GLU	A	6	116.253	2.401	202.308
ATOM	3	O	GLU	A	6	117.186	2.623	201.525
ATOM	4	H	GLU	A	6	118.296	3.461	203.475
ATOM	5	CB	GLU	A	6	115.358	3.289	204.369
ATOM	6	CG	GLU	A	6	115.377	3.208	205.887
ATOM	7	CD	GLU	A	6	114.327	4.108	206.518
ATOM	8	OE1	GLU	A	6	114.137	4.074	207.743
ATOM	9	OE2	GLU	A	6	113.661	4.876	205.809
ATOM	0	N	ASN	A	7	115.031	2.065	201.900
ATOM	1	CA	ASN	A	7	114.716	1.968	200.481
ATOM	2	C	ASN	A	7	116.047	1.680	199.740
ATOM	3	O	ASN	A	7	116.373	2.297	198.723
ATOM	4	H	ASN	A	7	114.334	1.882	202.561
ATOM	5	CB	ASN	A	7	114.109	3.291	199.980
ATOM	6	CG	ASN	A	7	112.685	3.487	200.472
ATOM	7	OD1	ASN	A	7	111.882	2.543	200.438
ATOM	8	ND2	ASN	A	7	112.427	4.725	200.917
ATOM	0	N	LEU	A	8	116.751	0.669	200.226

```
ATOM 1 CA LEU A 8 117.846 0.090 199.424
ATOM 2 C LEU A 8 117.295 -1.222 198.833
ATOM 3 O LEU A 8 116.433 -1.854 199.448
ATOM 4 H LEU A 8 116.546 0.307 201.110
ATOM 5 CB LEU A 8 119.068 -0.199 200.316
ATOM 6 CG LEU A 8 119.635 1.069 200.933
ATOM 7 CD1 LEU A 8 120.803 0.777 201.862
ATOM 8 CD2 LEU A 8 120.135 2.042 199.878
ATOM 0 N PRO A 9 117.768 -1.602 197.647
ATOM 1 CA PRO A 9 117.335 -2.842 197.003
ATOM 2 C PRO A 9 118.372 -3.919 197.444
ATOM 3 O PRO A 9 117.970 -5.060 197.638
ATOM 4 CB PRO A 9 117.356 -2.615 195.480
ATOM 5 CG PRO A 9 117.221 -1.088 195.331
ATOM 6 CD PRO A 9 117.913 -0.549 196.586
ATOM 0 N ASP A 10 119.647 -3.571 197.562
ATOM 1 CA ASP A 10 120.661 -4.569 197.849
ATOM 2 C ASP A 10 121.383 -3.923 199.086
ATOM 3 O ASP A 10 121.114 -2.795 199.483
ATOM 4 H ASP A 10 119.906 -2.634 197.456
ATOM 5 CB ASP A 10 121.628 -4.750 196.664
ATOM 6 CG ASP A 10 120.986 -5.511 195.516
ATOM 7 OD1 ASP A 10 120.133 -6.381 195.749
ATOM 8 OD2 ASP A 10 121.314 -5.262 194.347
ATOM 0 N GLU A 11 122.289 -4.701 199.678
ATOM 1 CA GLU A 11 123.130 -4.210 200.744
ATOM 2 C GLU A 11 124.117 -3.183 200.215
ATOM 3 O GLU A 11 124.497 -2.275 200.958
ATOM 4 H GLU A 11 122.388 -5.627 199.382
ATOM 5 CB GLU A 11 123.908 -5.387 201.360
ATOM 6 CG GLU A 11 122.987 -6.378 202.053
ATOM 7 CD GLU A 11 123.750 -7.565 202.620
ATOM 8 OE1 GLU A 11 123.160 -8.407 203.312
ATOM 9 OE2 GLU A 11 124.962 -7.690 202.389
ATOM 0 N ALA A 12 124.614 -3.407 198.995
ATOM 1 CA ALA A 12 125.799 -2.667 198.504
ATOM 2 C ALA A 12 125.392 -1.205 198.316
ATOM 3 O ALA A 12 126.258 -0.329 198.294
ATOM 4 H ALA A 12 124.189 -4.070 198.416
ATOM 5 CB ALA A 12 126.267 -3.256 197.160
ATOM 0 N ASP A 13 124.095 -0.932 198.199
ATOM 1 CA ASP A 13 123.626 0.438 197.999
ATOM 2 C ASP A 13 123.824 1.314 199.234
ATOM 3 O ASP A 13 123.910 2.541 199.149
ATOM 4 H ASP A 13 123.444 -1.660 198.248
ATOM 5 CB ASP A 13 122.125 0.401 197.658
ATOM 6 CG ASP A 13 121.864 -0.236 196.303
ATOM 7 OD1 ASP A 13 122.764 -0.270 195.450
ATOM 8 OD2 ASP A 13 120.751 -0.721 196.050
ATOM 0 N ARG A 14 123.896 0.671 200.396
ATOM 1 CA ARG A 14 124.047 1.398 201.655
```

```
ATOM      2  C    ARG A  14      125.525    1.656 201.755
ATOM      3  O    ARG A  14      125.930    2.742 202.139
ATOM      4  H    ARG A  14      123.848   -0.305 200.409
ATOM      5  CB   ARG A  14      123.561    0.546 202.841
ATOM      6  CG   ARG A  14      122.058    0.320 202.802
ATOM      7  CD   ARG A  14      121.569   -0.470 204.005
ATOM      8  NE   ARG A  14      120.125   -0.739 203.938
ATOM      9  CZ   ARG A  14      119.530   -1.474 204.901
ATOM     10  NH1  ARG A  14      118.214   -1.719 204.839
ATOM     11  NH2  ARG A  14      120.255   -1.958 205.918
ATOM      0  N    ASP A  15      126.360    0.705 201.375
ATOM      1  CA   ASP A  15      127.811    0.964 201.410
ATOM      2  C    ASP A  15      128.198    2.053 200.442
ATOM      3  O    ASP A  15      129.003    2.908 200.776
ATOM      4  H    ASP A  15      126.020   -0.160 201.073
ATOM      5  CB   ASP A  15      128.561   -0.327 201.036
ATOM      6  CG   ASP A  15      128.434   -1.392 202.113
ATOM      7  OD1  ASP A  15      128.343   -1.060 203.305
ATOM      8  OD2  ASP A  15      128.423   -2.592 201.801
ATOM      0  N    LYS A  16      127.608    2.090 199.261
ATOM      1  CA   LYS A  16      127.925    3.188 198.330
ATOM      2  C    LYS A  16      127.201    4.506 198.585
ATOM      3  O    LYS A  16      127.770    5.583 198.417
ATOM      4  H    LYS A  16      126.967    1.397 199.006
ATOM      5  CB   LYS A  16      127.567    2.722 196.907
ATOM      6  CG   LYS A  16      128.472    1.597 196.432
ATOM      7  CD   LYS A  16      128.135    1.153 195.018
ATOM      8  CE   LYS A  16      129.039    0.027 194.543
ATOM      9  NZ   LYS A  16      128.711   -0.405 193.166
ATOM      0  N    TYR A  17      125.927    4.417 198.974
ATOM      1  CA   TYR A  17      125.137    5.622 199.223
ATOM      2  C    TYR A  17      124.663    5.360 200.744
ATOM      3  O    TYR A  17      124.890    4.328 201.364
ATOM      4  H    TYR A  17      125.518    3.538 199.096
ATOM      5  CB   TYR A  17      123.936    5.723 198.264
ATOM      6  CG   TYR A  17      124.372    5.969 196.829
ATOM      7  CD1  TYR A  17      124.382    4.937 195.929
ATOM      8  CE1  TYR A  17      124.775    5.159 194.635
ATOM      9  CZ   TYR A  17      125.158    6.415 194.242
ATOM     10  CE2  TYR A  17      125.149    7.447 195.142
ATOM     11  CD2  TYR A  17      124.756    7.225 196.436
ATOM     12  OH   TYR A  17      125.554    6.639 192.939
ATOM      0  N    GLU A  18      124.004    6.391 201.272
ATOM      1  CA   GLU A  18      123.378    6.306 202.571
ATOM      2  C    GLU A  18      122.631    7.558 202.767
ATOM      3  O    GLU A  18      123.005    8.616 202.240
ATOM      4  H    GLU A  18      123.942    7.227 200.769
ATOM      5  CB   GLU A  18      124.448    6.152 203.668
ATOM      6  CG   GLU A  18      123.830    5.997 205.048
ATOM      7  CD   GLU A  18      124.884    5.784 206.122
ATOM      8  OE1  GLU A  18      125.839    5.022 205.912
```

ATOM	9	OE2	GLU	A	18	124.788	6.372	207.210
ATOM	0	N	LEU	A	19	121.534	7.459	203.508
ATOM	1	CA	LEU	A	19	120.703	8.609	203.825
ATOM	2	C	LEU	A	19	121.289	9.164	205.054
ATOM	3	O	LEU	A	19	121.547	8.324	205.916
ATOM	4	H	LEU	A	19	121.275	6.583	203.854
ATOM	5	CB	LEU	A	19	119.245	8.177	204.067
ATOM	6	CG	LEU	A	19	118.608	7.595	202.815
ATOM	7	CD1	LEU	A	19	117.264	6.950	203.108
ATOM	8	CD2	LEU	A	19	118.373	8.654	201.751
ATOM	0	N	LEU	A	20	121.339	10.472	205.281
ATOM	1	CA	LEU	A	20	121.883	11.014	206.520
ATOM	2	C	LEU	A	20	120.497	11.670	207.051
ATOM	3	O	LEU	A	20	119.499	12.001	206.404
ATOM	4	H	LEU	A	20	121.001	11.087	204.601
ATOM	5	CB	LEU	A	20	122.981	12.066	206.277
ATOM	6	CG	LEU	A	20	124.239	11.450	205.687
ATOM	7	CD1	LEU	A	20	125.227	12.508	205.222
ATOM	8	CD2	LEU	A	20	124.966	10.569	206.690
ATOM	0	N	CYS	A	21	120.552	11.656	208.377
ATOM	1	CA	CYS	A	21	119.459	12.125	209.228
ATOM	2	C	CYS	A	21	119.651	12.255	210.715
ATOM	3	O	CYS	A	21	120.806	12.238	211.137
ATOM	4	H	CYS	A	21	121.362	11.317	208.807
ATOM	5	CB	CYS	A	21	118.286	11.149	209.020
ATOM	6	SG	CYS	A	21	117.668	11.044	207.311
ATOM	0	N	PRO	A	22	118.607	12.579	211.471
ATOM	1	CA	PRO	A	22	118.766	12.943	212.874
ATOM	2	C	PRO	A	22	118.451	11.656	213.701
ATOM	3	O	PRO	A	22	119.195	11.260	214.605
ATOM	4	CB	PRO	A	22	117.763	14.071	213.178
ATOM	5	CG	PRO	A	22	117.489	14.705	211.801
ATOM	6	CD	PRO	A	22	117.639	13.526	210.836
ATOM	0	N	ASP	A	23	117.244	11.155	213.502
ATOM	1	CA	ASP	A	23	116.657	10.189	214.426
ATOM	2	C	ASP	A	23	117.538	9.004	214.773
ATOM	3	O	ASP	A	23	118.444	8.562	214.057
ATOM	4	H	ASP	A	23	116.733	11.437	212.718
ATOM	5	CB	ASP	A	23	115.363	9.647	213.792
ATOM	6	CG	ASP	A	23	114.287	10.717	213.693
ATOM	7	OD1	ASP	A	23	114.222	11.612	214.548
ATOM	8	OD2	ASP	A	23	113.475	10.694	212.756
ATOM	0	N	ASN	A	24	117.269	8.497	215.981
ATOM	1	CA	ASN	A	24	118.169	7.435	216.484
ATOM	2	C	ASN	A	24	118.493	6.392	215.634
ATOM	3	O	ASN	A	24	118.536	5.453	216.434
ATOM	4	H	ASN	A	24	116.510	8.813	216.509
ATOM	5	CB	ASN	A	24	117.500	6.818	217.726
ATOM	6	CG	ASN	A	24	117.453	7.793	218.891
ATOM	7	OD1	ASN	A	24	118.449	8.477	219.167
ATOM	8	ND2	ASN	A	24	116.278	7.816	219.534

ATOM 0 N THR A 25 118.513 6.233 214.315
ATOM 1 CA THR A 25 118.611 4.927 213.663
ATOM 2 C THR A 25 119.982 5.016 213.078
ATOM 3 O THR A 25 120.457 6.044 212.592
ATOM 4 H THR A 25 118.460 7.029 213.750
ATOM 5 CB THR A 25 117.538 4.752 212.572
ATOM 6 OG1 THR A 25 116.236 4.742 213.162
ATOM 7 CG2 THR A 25 117.715 3.453 211.802
ATOM 0 N ARG A 26 120.654 3.871 213.143
ATOM 1 CA ARG A 26 122.011 3.758 212.630
ATOM 2 C ARG A 26 122.035 3.270 211.192
ATOM 3 O ARG A 26 121.066 2.688 210.702
ATOM 4 H ARG A 26 120.229 3.087 213.543
ATOM 5 CB ARG A 26 122.795 2.763 213.505
ATOM 6 CG ARG A 26 124.259 2.684 213.106
ATOM 7 CD ARG A 26 125.137 2.205 214.251
ATOM 8 NE ARG A 26 126.565 2.240 213.902
ATOM 9 CZ ARG A 26 127.489 1.848 214.804
ATOM 10 NH1 ARG A 26 128.791 1.881 214.486
ATOM 11 NH2 ARG A 26 127.106 1.426 216.017
TER
ENDMDL
MODEL 3
ATOM 0 N ASP A 1 125.573 13.479 212.939
ATOM 1 CA ASP A 1 126.644 12.543 213.272
ATOM 2 C ASP A 1 126.556 11.113 212.738
ATOM 3 O ASP A 1 127.586 10.495 212.462
ATOM 4 H ASP A 1 125.608 14.439 213.282
ATOM 5 CB ASP A 1 126.709 12.451 214.808
ATOM 6 CG ASP A 1 127.143 13.764 215.439
ATOM 7 OD1 ASP A 1 127.928 14.512 214.838
ATOM 8 OD2 ASP A 1 126.712 14.087 216.556
ATOM 0 N SER A 2 125.352 10.573 212.585
ATOM 1 CA SER A 2 125.244 9.207 212.082
ATOM 2 C SER A 2 124.556 9.069 210.733
ATOM 3 O SER A 2 123.832 9.955 210.280
ATOM 4 H SER A 2 124.548 11.083 212.805
ATOM 5 CB SER A 2 124.448 8.380 213.108
ATOM 6 OG SER A 2 123.103 8.865 213.119
ATOM 0 N THR A 3 124.805 7.932 210.094
ATOM 1 CA THR A 3 124.226 7.621 208.803
ATOM 2 C THR A 3 122.882 6.951 209.040
ATOM 3 O THR A 3 122.725 6.188 209.991
ATOM 4 H THR A 3 125.401 7.279 210.511
ATOM 5 CB THR A 3 125.154 6.666 208.029
ATOM 6 OG1 THR A 3 126.417 7.296 207.794
ATOM 7 CG2 THR A 3 124.570 6.271 206.682
ATOM 0 N VAL A 4 121.888 7.235 208.186
ATOM 1 CA VAL A 4 120.587 6.598 208.393
ATOM 2 C VAL A 4 120.473 5.841 207.041
ATOM 3 O VAL A 4 120.910 6.235 205.963

ATOM 4 H VAL A 4 122.028 7.852 207.442
ATOM 5 CB VAL A 4 119.458 7.630 208.574
ATOM 6 CG1 VAL A 4 119.039 8.253 207.252
ATOM 7 CG2 VAL A 4 118.215 7.012 209.191
ATOM 0 N PHE A 5 119.883 4.664 207.217
ATOM 1 CA PHE A 5 119.488 3.786 206.085
ATOM 2 C PHE A 5 118.077 3.353 205.843
ATOM 3 O PHE A 5 117.460 2.843 206.774
ATOM 4 H PHE A 5 119.701 4.362 208.129
ATOM 5 CB PHE A 5 120.306 2.491 206.246
ATOM 6 CG PHE A 5 119.959 1.463 205.181
ATOM 7 CD1 PHE A 5 120.456 1.597 203.911
ATOM 8 CE1 PHE A 5 120.143 0.671 202.952
ATOM 9 CZ PHE A 5 119.334 -0.390 203.262
ATOM 10 CE2 PHE A 5 118.837 -0.525 204.532
ATOM 11 CD2 PHE A 5 119.150 0.402 205.491
ATOM 0 N GLU A 6 117.545 3.535 204.643
ATOM 1 CA GLU A 6 116.175 3.148 204.337
ATOM 2 C GLU A 6 116.109 1.818 203.728
ATOM 3 O GLU A 6 115.098 1.138 203.836
ATOM 4 H GLU A 6 118.089 3.941 203.940
ATOM 5 CB GLU A 6 115.573 4.174 203.358
ATOM 6 CG GLU A 6 115.445 5.552 203.985
ATOM 7 CD GLU A 6 114.896 6.575 203.004
ATOM 8 OE1 GLU A 6 114.627 7.722 203.391
ATOM 9 OE2 GLU A 6 114.718 6.263 201.818
ATOM 0 N ASN A 7 117.172 1.413 203.038
ATOM 1 CA ASN A 7 117.297 0.056 202.492
ATOM 2 C ASN A 7 117.553 -1.750 202.579
ATOM 3 O ASN A 7 117.699 -2.649 201.723
ATOM 4 H ASN A 7 117.902 2.046 202.889
ATOM 5 CB ASN A 7 118.420 0.357 201.483
ATOM 6 CG ASN A 7 118.079 1.536 200.586
ATOM 7 OD1 ASN A 7 116.946 1.631 200.091
ATOM 8 ND2 ASN A 7 119.090 2.399 200.412
ATOM 0 N LEU A 8 118.038 -1.848 203.788
ATOM 1 CA LEU A 8 118.859 -2.971 204.177
ATOM 2 C LEU A 8 119.100 -4.254 203.290
ATOM 3 O LEU A 8 120.151 -4.862 203.149
ATOM 4 H LEU A 8 117.841 -1.149 204.442
ATOM 5 CB LEU A 8 118.256 -3.489 205.495
ATOM 6 CG LEU A 8 118.163 -2.396 206.548
ATOM 7 CD1 LEU A 8 117.352 -2.836 207.756
ATOM 8 CD2 LEU A 8 119.533 -1.979 207.056
ATOM 0 N PRO A 9 118.001 -4.558 202.606
ATOM 1 CA PRO A 9 117.993 -5.530 201.504
ATOM 2 C PRO A 9 118.875 -5.132 200.322
ATOM 3 O PRO A 9 119.314 -6.024 199.595
ATOM 4 CB PRO A 9 116.525 -5.633 201.050
ATOM 5 CG PRO A 9 115.726 -5.095 202.253
ATOM 6 CD PRO A 9 116.672 -4.061 202.870

```
ATOM      0  N   ASP A  10     119.156  -3.847 200.127
ATOM      1  CA  ASP A  10     119.985  -3.416 199.003
ATOM      2  C   ASP A  10     121.405  -3.072 199.321
ATOM      3  O   ASP A  10     121.667  -2.070 199.984
ATOM      4  H   ASP A  10     118.804  -3.178 200.747
ATOM      5  CB  ASP A  10     119.328  -2.168 198.384
ATOM      6  CG  ASP A  10     117.994  -2.491 197.732
ATOM      7  OD1 ASP A  10     117.811  -3.601 197.210
ATOM      8  OD2 ASP A  10     117.088  -1.644 197.722
ATOM      0  N   GLU A  11     122.344  -3.892 198.869
ATOM      1  CA  GLU A  11     123.767  -3.713 199.099
ATOM      2  C   GLU A  11     124.390  -2.799 198.100
ATOM      3  O   GLU A  11     125.185  -1.951 198.510
ATOM      4  H   GLU A  11     122.061  -4.668 198.345
ATOM      5  CB  GLU A  11     124.461  -5.086 199.013
ATOM      6  CG  GLU A  11     124.008  -6.023 200.121
ATOM      7  CD  GLU A  11     124.656  -7.393 200.008
ATOM      8  OE1 GLU A  11     124.479  -8.238 200.897
ATOM      9  OE2 GLU A  11     125.363  -7.666 199.026
ATOM      0  N   ALA A  12     124.061  -2.939 196.817
ATOM      1  CA  ALA A  12     124.636  -2.094 195.773
ATOM      2  C   ALA A  12     124.133  -0.675 196.005
ATOM      3  O   ALA A  12     124.892   0.267 195.868
ATOM      4  H   ALA A  12     123.415  -3.628 196.565
ATOM      5  CB  ALA A  12     124.187  -2.590 194.386
ATOM      0  N   ASP A  13     122.864  -0.449 196.348
ATOM      1  CA  ASP A  13     122.395   0.891 196.618
ATOM      2  C   ASP A  13     123.052   1.543 197.837
ATOM      3  O   ASP A  13     123.422   2.719 197.790
ATOM      4  H   ASP A  13     122.242  -1.199 196.420
ATOM      5  CB  ASP A  13     120.875   0.835 196.860
ATOM      6  CG  ASP A  13     120.110   0.479 195.596
ATOM      7  OD1 ASP A  13     120.541   0.835 194.489
ATOM      8  OD2 ASP A  13     119.055  -0.168 195.672
ATOM      0  N   ARG A  14     123.203   0.791 198.926
ATOM      1  CA  ARG A  14     123.802   1.359 200.130
ATOM      2  C   ARG A  14     125.269   1.647 199.769
ATOM      3  O   ARG A  14     125.737   2.688 200.203
ATOM      4  H   ARG A  14     122.914  -0.143 198.920
ATOM      5  CB  ARG A  14     123.724   0.356 201.295
ATOM      6  CG  ARG A  14     124.291   0.934 202.582
ATOM      7  CD  ARG A  14     123.720   0.249 203.813
ATOM      8  NE  ARG A  14     124.178   0.884 205.058
ATOM      9  CZ  ARG A  14     123.767   0.403 206.251
ATOM     10  NH1 ARG A  14     124.184   0.982 207.386
ATOM     11  NH2 ARG A  14     122.943  -0.652 206.301
ATOM      0  N   ASP A  15     125.975   0.847 198.991
ATOM      1  CA  ASP A  15     127.355   1.223 198.633
ATOM      2  C   ASP A  15     127.322   2.592 197.912
ATOM      3  O   ASP A  15     128.146   3.434 198.280
ATOM      4  H   ASP A  15     125.587   0.015 198.654
```

ATOM	5	CB	ASP	A	15	127.959	0.159	197.698
ATOM	6	CG	ASP	A	15	128.236	-1.147	198.425
ATOM	7	OD1	ASP	A	15	128.553	-1.134	199.623
ATOM	8	OD2	ASP	A	15	128.144	-2.225	197.819
ATOM	0	N	LYS	A	16	126.428	2.813	196.952
ATOM	1	CA	LYS	A	16	126.358	4.067	196.207
ATOM	2	C	LYS	A	16	126.107	5.267	197.129
ATOM	3	O	LYS	A	16	126.769	6.298	196.989
ATOM	4	H	LYS	A	16	125.789	2.106	196.736
ATOM	5	CB	LYS	A	16	125.209	3.978	195.185
ATOM	6	CG	LYS	A	16	125.515	2.991	194.070
ATOM	7	CD	LYS	A	16	124.392	2.920	193.049
ATOM	8	CE	LYS	A	16	124.697	1.933	191.934
ATOM	9	NZ	LYS	A	16	123.603	1.865	190.939
ATOM	0	N	TYR	A	17	125.168	5.143	198.064
ATOM	1	CA	TYR	A	17	124.872	6.261	198.955
ATOM	2	C	TYR	A	17	124.394	6.296	200.304
ATOM	3	O	TYR	A	17	124.251	5.165	200.765
ATOM	4	H	TYR	A	17	124.676	4.303	198.156
ATOM	5	CB	TYR	A	17	123.828	7.084	198.177
ATOM	6	CG	TYR	A	17	124.318	7.457	196.787
ATOM	7	CD1	TYR	A	17	123.869	6.763	195.694
ATOM	8	CE1	TYR	A	17	124.310	7.099	194.442
ATOM	9	CZ	TYR	A	17	125.200	8.128	194.282
ATOM	10	CE2	TYR	A	17	125.649	8.822	195.375
ATOM	11	CD2	TYR	A	17	125.207	8.486	196.628
ATOM	12	OH	TYR	A	17	125.644	8.467	193.020
ATOM	0	N	GLU	A	18	124.126	7.372	201.013
ATOM	1	CA	GLU	A	18	123.651	7.225	202.374
ATOM	2	C	GLU	A	18	123.102	8.544	202.896
ATOM	3	O	GLU	A	18	123.594	9.602	202.483
ATOM	4	H	GLU	A	18	124.244	8.261	200.626
ATOM	5	CB	GLU	A	18	124.818	6.774	203.273
ATOM	6	CG	GLU	A	18	125.312	5.383	202.909
ATOM	7	CD	GLU	A	18	126.496	4.958	203.763
ATOM	8	OE1	GLU	A	18	126.946	3.807	203.668
ATOM	9	OE2	GLU	A	18	127.010	5.762	204.554
ATOM	0	N	LEU	A	19	122.059	8.488	203.706
ATOM	1	CA	LEU	A	19	121.425	9.661	204.258
ATOM	2	C	LEU	A	19	122.226	10.008	205.446
ATOM	3	O	LEU	A	19	122.521	9.057	206.170
ATOM	4	H	LEU	A	19	121.699	7.611	203.944
ATOM	5	CB	LEU	A	19	119.971	9.354	204.659
ATOM	6	CG	LEU	A	19	119.113	8.995	203.457
ATOM	7	CD1	LEU	A	19	117.751	8.458	203.869
ATOM	8	CD2	LEU	A	19	118.867	10.192	202.554
ATOM	0	N	LEU	A	20	122.460	11.272	205.781
ATOM	1	CA	LEU	A	20	123.254	11.620	206.953
ATOM	2	C	LEU	A	20	122.068	12.170	207.847
ATOM	3	O	LEU	A	20	121.209	12.920	207.387
ATOM	4	H	LEU	A	20	122.090	11.988	205.229

ATOM 5 CB LEU A 20 124.308 12.700 206.649
ATOM 6 CG LEU A 20 125.398 12.188 205.721
ATOM 7 CD1 LEU A 20 126.298 13.308 205.227
ATOM 8 CD2 LEU A 20 126.290 11.163 206.401
ATOM 0 N CYS A 21 122.042 11.772 209.103
ATOM 1 CA CYS A 21 121.057 12.324 210.046
ATOM 2 C CYS A 21 121.648 12.162 211.544
ATOM 3 O CYS A 21 122.750 11.629 211.727
ATOM 4 H CYS A 21 122.684 11.103 209.412
ATOM 5 CB CYS A 21 119.729 11.554 209.924
ATOM 6 SG CYS A 21 118.872 11.745 208.330
ATOM 0 N PRO A 22 120.870 12.578 212.543
ATOM 1 CA PRO A 22 121.273 12.395 213.931
ATOM 2 C PRO A 22 120.332 11.214 214.385
ATOM 3 O PRO A 22 120.692 10.424 215.255
ATOM 4 CB PRO A 22 120.999 13.700 214.701
ATOM 5 CG PRO A 22 121.048 14.790 213.613
ATOM 6 CD PRO A 22 120.545 14.062 212.363
ATOM 0 N ASP A 23 119.067 11.350 214.012
ATOM 1 CA ASP A 23 118.040 10.528 214.636
ATOM 2 C ASP A 23 118.232 9.081 214.221
ATOM 3 O ASP A 23 118.893 8.795 213.211
ATOM 4 H ASP A 23 118.825 11.997 213.321
ATOM 5 CB ASP A 23 116.649 11.010 214.185
ATOM 6 CG ASP A 23 116.296 12.364 214.778
ATOM 7 OD1 ASP A 23 116.718 12.679 215.901
ATOM 8 OD2 ASP A 23 115.585 13.155 214.139
ATOM 0 N ASN A 24 117.699 8.160 215.010
ATOM 1 CA ASN A 24 117.767 6.749 214.654
ATOM 2 C ASN A 24 116.752 6.339 213.711
ATOM 3 O ASN A 24 116.498 5.151 213.934
ATOM 4 H ASN A 24 117.255 8.428 215.838
ATOM 5 CB ASN A 24 117.603 5.914 215.937
ATOM 6 CG ASN A 24 118.815 6.024 216.847
ATOM 7 OD1 ASN A 24 119.957 5.958 216.369
ATOM 8 ND2 ASN A 24 118.512 6.190 218.141
ATOM 0 N THR A 25 115.994 7.097 212.924
ATOM 1 CA THR A 25 114.804 6.558 212.282
ATOM 2 C THR A 25 115.245 5.411 211.245
ATOM 3 O THR A 25 116.262 5.570 210.564
ATOM 4 H THR A 25 116.240 8.031 212.774
ATOM 5 CB THR A 25 114.069 7.681 211.526
ATOM 6 OG1 THR A 25 113.655 8.698 212.442
ATOM 7 CG2 THR A 25 112.834 7.168 210.804
ATOM 0 N ARG A 26 114.508 4.312 211.171
ATOM 1 CA ARG A 26 114.939 3.194 210.344
ATOM 2 C ARG A 26 114.869 3.523 208.871
ATOM 3 O ARG A 26 113.860 4.051 208.419
ATOM 4 H ARG A 26 113.671 4.251 211.673
ATOM 5 CB ARG A 26 114.030 1.984 210.627
ATOM 6 CG ARG A 26 114.494 0.739 209.889

```
ATOM     7  CD  ARG A  26     114.017  -0.535 210.568
ATOM     8  NE  ARG A  26     114.563  -1.739 209.924
ATOM     9  CZ  ARG A  26     114.243  -2.962 210.398
ATOM    10  NH1 ARG A  26     114.740  -4.059 209.811
ATOM    11  NH2 ARG A  26     113.428  -3.079 211.455
TER
ENDMDL
MODEL        4
ATOM     0  N   ASP A   1     119.848 -11.352 210.858
ATOM     1  CA  ASP A   1     118.501 -11.556 210.354
ATOM     2  C   ASP A   1     118.221 -10.856 209.032
ATOM     3  O   ASP A   1     117.356 -11.281 208.267
ATOM     4  H   ASP A   1     120.133 -11.781 211.739
ATOM     5  CB  ASP A   1     117.505 -11.024 211.402
ATOM     6  CG  ASP A   1     117.506 -11.866 212.667
ATOM     7  OD1 ASP A   1     117.734 -13.083 212.601
ATOM     8  OD2 ASP A   1     117.279 -11.336 213.765
ATOM     0  N   SER A   2     118.957  -9.785 208.761
ATOM     1  CA  SER A   2     118.710  -8.967 207.577
ATOM     2  C   SER A   2     119.440  -9.493 206.338
ATOM     3  O   SER A   2     120.671  -9.568 206.313
ATOM     4  H   SER A   2     119.682  -9.537 209.368
ATOM     5  CB  SER A   2     119.190  -7.531 207.858
ATOM     6  OG  SER A   2     120.612  -7.561 208.004
ATOM     0  N   THR A   3     118.678  -9.857 205.312
ATOM     1  CA  THR A   3     119.260 -10.253 204.036
ATOM     2  C   THR A   3     120.139  -9.125 203.513
ATOM     3  O   THR A   3     119.822  -7.948 203.689
ATOM     4  H   THR A   3     117.706  -9.860 205.415
ATOM     5  CB  THR A   3     118.136 -10.540 203.023
ATOM     6  OG1 THR A   3     117.317 -11.614 203.492
ATOM     7  CG2 THR A   3     118.684 -10.929 201.660
ATOM     0  N   VAL A   4     121.242  -9.488 202.868
ATOM     1  CA  VAL A   4     122.258  -8.518 202.480
ATOM     2  C   VAL A   4     121.797  -7.617 201.391
ATOM     3  O   VAL A   4     122.392  -6.548 201.245
ATOM     4  H   VAL A   4     121.376 -10.431 202.647
ATOM     5  CB  VAL A   4     123.508  -9.275 201.997
ATOM     6  CG1 VAL A   4     124.565  -8.334 201.442
ATOM     7  CG2 VAL A   4     124.160 -10.071 203.116
ATOM     0  N   PHE A   5     120.634  -7.912 200.801
ATOM     1  CA  PHE A   5     120.049  -7.019 199.776
ATOM     2  C   PHE A   5     119.364  -5.950 200.653
ATOM     3  O   PHE A   5     119.433  -4.770 200.299
ATOM     4  H   PHE A   5     120.161  -8.730 201.051
ATOM     5  CB  PHE A   5     119.026  -7.763 198.896
ATOM     6  CG  PHE A   5     119.699  -8.726 197.932
ATOM     7  CD1 PHE A   5     119.875 -10.038 198.286
ATOM     8  CE1 PHE A   5     120.481 -10.906 197.417
ATOM     9  CZ  PHE A   5     120.911 -10.463 196.194
ATOM    10  CE2 PHE A   5     120.734  -9.151 195.840
```

ATOM 11 CD2 PHE A 5 120.128 -8.283 196.709
ATOM 0 N GLU A 6 118.722 -6.328 201.757
ATOM 1 CA GLU A 6 118.046 -5.334 202.585
ATOM 2 C GLU A 6 119.238 -4.525 203.220
ATOM 3 O GLU A 6 119.116 -3.301 203.221
ATOM 4 H GLU A 6 118.704 -7.270 202.015
ATOM 5 CB GLU A 6 117.195 -6.009 203.677
ATOM 6 CG GLU A 6 115.991 -6.733 203.095
ATOM 7 CD GLU A 6 115.178 -7.440 204.167
ATOM 8 OE1 GLU A 6 114.104 -7.984 203.872
ATOM 9 OE2 GLU A 6 115.590 -7.473 205.336
ATOM 0 N ASN A 7 120.276 -5.177 203.726
ATOM 1 CA ASN A 7 121.382 -4.461 204.338
ATOM 2 C ASN A 7 122.065 -3.469 203.380
ATOM 3 O ASN A 7 122.345 -2.333 203.770
ATOM 4 H ASN A 7 120.298 -6.153 203.686
ATOM 5 CB ASN A 7 122.430 -5.486 204.808
ATOM 6 CG ASN A 7 123.013 -6.278 203.649
ATOM 7 OD1 ASN A 7 122.889 -5.859 202.488
ATOM 8 ND2 ASN A 7 123.633 -7.407 204.017
ATOM 0 N LEU A 8 122.321 -3.881 202.139
ATOM 1 CA LEU A 8 122.979 -2.986 201.193
ATOM 2 C LEU A 8 122.005 -1.816 200.857
ATOM 3 O LEU A 8 122.501 -0.690 200.766
ATOM 4 H LEU A 8 122.068 -4.784 201.862
ATOM 5 CB LEU A 8 123.328 -3.755 199.905
ATOM 6 CG LEU A 8 123.962 -2.853 198.858
ATOM 7 CD1 LEU A 8 125.283 -2.269 199.334
ATOM 8 CD2 LEU A 8 124.243 -3.596 197.562
ATOM 0 N PRO A 9 120.703 -2.038 200.682
ATOM 1 CA PRO A 9 119.812 -0.932 200.345
ATOM 2 C PRO A 9 119.726 0.028 201.570
ATOM 3 O PRO A 9 119.724 1.237 201.328
ATOM 4 CB PRO A 9 118.436 -1.543 200.022
ATOM 5 CG PRO A 9 118.758 -3.001 199.644
ATOM 6 CD PRO A 9 119.990 -3.322 200.494
ATOM 0 N ASP A 10 119.660 -0.451 202.811
ATOM 1 CA ASP A 10 119.556 0.465 203.943
ATOM 2 C ASP A 10 120.893 1.257 204.066
ATOM 3 O ASP A 10 120.803 2.455 204.344
ATOM 4 H ASP A 10 119.682 -1.416 202.965
ATOM 5 CB ASP A 10 119.305 -0.333 205.237
ATOM 6 CG ASP A 10 117.910 -0.935 205.274
ATOM 7 OD1 ASP A 10 116.967 -0.348 204.725
ATOM 8 OD2 ASP A 10 117.716 -2.015 205.852
ATOM 0 N GLU A 11 122.065 0.654 203.876
ATOM 1 CA GLU A 11 123.307 1.408 204.019
ATOM 2 C GLU A 11 123.271 2.481 202.935
ATOM 3 O GLU A 11 123.525 3.655 203.195
ATOM 4 H GLU A 11 122.095 -0.294 203.640
ATOM 5 CB GLU A 11 124.523 0.485 203.817

ATOM	6	CG	GLU	A	11	124.658	-0.532	204.939
ATOM	7	CD	GLU	A	11	125.829	-1.474	204.715
ATOM	8	OE1	GLU	A	11	126.144	-2.292	205.592
ATOM	9	OE2	GLU	A	11	126.470	-1.426	203.654
ATOM	0	N	ALA	A	12	122.889	2.120	201.732
ATOM	1	CA	ALA	A	12	122.885	3.120	200.695
ATOM	2	C	ALA	A	12	121.949	4.259	200.985
ATOM	3	O	ALA	A	12	122.269	5.421	200.788
ATOM	4	H	ALA	A	12	122.615	1.201	201.545
ATOM	5	CB	ALA	A	12	122.455	2.457	199.373
ATOM	0	N	ASP	A	13	120.788	3.949	201.533
ATOM	1	CA	ASP	A	13	119.862	5.029	201.919
ATOM	2	C	ASP	A	13	120.384	5.935	203.039
ATOM	3	O	ASP	A	13	120.241	7.158	202.969
ATOM	4	H	ASP	A	13	120.545	3.014	201.681
ATOM	5	CB	ASP	A	13	118.543	4.390	202.392
ATOM	6	CG	ASP	A	13	117.796	3.716	201.253
ATOM	7	OD1	ASP	A	13	117.877	4.171	200.102
ATOM	8	OD2	ASP	A	13	117.106	2.710	201.474
ATOM	0	N	ARG	A	14	120.995	5.346	204.066
ATOM	1	CA	ARG	A	14	121.501	6.151	205.174
ATOM	2	C	ARG	A	14	122.653	6.985	204.592
ATOM	3	O	ARG	A	14	122.730	8.142	204.976
ATOM	4	H	ARG	A	14	121.105	4.375	204.078
ATOM	5	CB	ARG	A	14	122.016	5.243	206.307
ATOM	6	CG	ARG	A	14	122.503	6.045	207.502
ATOM	7	CD	ARG	A	14	122.457	5.236	208.788
ATOM	8	NE	ARG	A	14	122.816	6.046	209.962
ATOM	9	CZ	ARG	A	14	122.827	5.486	211.190
ATOM	10	NH1	ARG	A	14	123.154	6.224	212.260
ATOM	11	NH2	ARG	A	14	122.512	4.192	211.340
ATOM	0	N	ASP	A	15	123.485	6.505	203.686
ATOM	1	CA	ASP	A	15	124.524	7.386	203.123
ATOM	2	C	ASP	A	15	123.920	8.570	202.427
ATOM	3	O	ASP	A	15	124.237	9.739	202.631
ATOM	4	H	ASP	A	15	123.413	5.576	203.391
ATOM	5	CB	ASP	A	15	125.366	6.588	202.110
ATOM	6	CG	ASP	A	15	126.223	5.531	202.789
ATOM	7	OD1	ASP	A	15	126.665	5.732	203.930
ATOM	8	OD2	ASP	A	15	126.479	4.470	202.202
ATOM	0	N	LYS	A	16	122.933	8.265	201.617
ATOM	1	CA	LYS	A	16	122.305	9.335	200.885
ATOM	2	C	LYS	A	16	121.618	10.408	201.732
ATOM	3	O	LYS	A	16	121.737	11.612	201.491
ATOM	4	H	LYS	A	16	122.632	7.341	201.514
ATOM	5	CB	LYS	A	16	121.242	8.716	199.957
ATOM	6	CG	LYS	A	16	121.868	7.869	198.861
ATOM	7	CD	LYS	A	16	120.820	7.278	197.931
ATOM	8	CE	LYS	A	16	121.446	6.432	196.834
ATOM	9	NZ	LYS	A	16	120.426	5.856	195.929
ATOM	0	N	TYR	A	17	120.905	9.953	202.761

ATOM	1	CA	TYR	A	17	120.208	10.889	203.638
ATOM	2	C	TYR	A	17	121.309	11.672	204.371
ATOM	3	O	TYR	A	17	121.110	12.867	204.527
ATOM	4	H	TYR	A	17	120.848	8.992	202.930
ATOM	5	CB	TYR	A	17	119.329	10.129	204.648
ATOM	6	CG	TYR	A	17	118.633	11.069	205.618
ATOM	7	CD1	TYR	A	17	117.529	11.774	205.217
ATOM	8	CE1	TYR	A	17	116.902	12.622	206.092
ATOM	9	CZ	TYR	A	17	117.379	12.764	207.368
ATOM	10	CE2	TYR	A	17	118.483	12.058	207.770
ATOM	11	CD2	TYR	A	17	119.110	11.211	206.895
ATOM	12	OH	TYR	A	17	116.747	13.617	208.250
ATOM	0	N	GLU	A	18	122.435	11.105	204.766
ATOM	1	CA	GLU	A	18	123.474	11.935	205.403
ATOM	2	C	GLU	A	18	124.038	13.009	204.536
ATOM	3	O	GLU	A	18	124.261	14.151	204.895
ATOM	4	H	GLU	A	18	122.579	10.147	204.637
ATOM	5	CB	GLU	A	18	124.630	11.012	205.831
ATOM	6	CG	GLU	A	18	124.221	10.069	206.951
ATOM	7	CD	GLU	A	18	125.342	9.116	207.332
ATOM	8	OE1	GLU	A	18	126.340	9.011	206.603
ATOM	9	OE2	GLU	A	18	125.260	8.445	208.371
ATOM	0	N	LEU	A	19	124.306	12.656	203.278
ATOM	1	CA	LEU	A	19	124.799	13.622	202.323
ATOM	2	C	LEU	A	19	123.806	14.743	202.009
ATOM	3	O	LEU	A	19	124.191	15.912	201.936
ATOM	4	H	LEU	A	19	124.166	11.730	202.997
ATOM	5	CB	LEU	A	19	125.128	12.885	201.011
ATOM	6	CG	LEU	A	19	126.188	11.814	201.210
ATOM	7	CD1	LEU	A	19	126.339	10.928	199.984
ATOM	8	CD2	LEU	A	19	127.554	12.412	201.501
ATOM	0	N	LEU	A	20	122.532	14.399	201.825
ATOM	1	CA	LEU	A	20	121.539	15.418	201.500
ATOM	2	C	LEU	A	20	121.188	16.377	202.643
ATOM	3	O	LEU	A	20	121.057	17.579	202.405
ATOM	4	H	LEU	A	20	122.261	13.463	201.909
ATOM	5	CB	LEU	A	20	120.246	14.704	201.066
ATOM	6	CG	LEU	A	20	120.425	13.947	199.760
ATOM	7	CD1	LEU	A	20	119.201	13.117	199.411
ATOM	8	CD2	LEU	A	20	120.680	14.882	198.589
ATOM	0	N	CYS	A	21	121.044	15.863	203.859
ATOM	1	CA	CYS	A	21	120.644	16.696	204.985
ATOM	2	C	CYS	A	21	121.145	15.758	206.225
ATOM	3	O	CYS	A	21	120.914	14.547	206.244
ATOM	4	H	CYS	A	21	121.210	14.911	204.002
ATOM	5	CB	CYS	A	21	119.121	16.921	205.019
ATOM	6	SG	CYS	A	21	118.469	17.974	203.685
ATOM	0	N	PRO	A	22	121.803	16.380	207.200
ATOM	1	CA	PRO	A	22	122.015	15.813	208.511
ATOM	2	C	PRO	A	22	120.759	15.679	209.258
ATOM	3	O	PRO	A	22	120.087	16.709	209.282

```
ATOM      4  CB  PRO A  22      122.974  16.770 209.243
ATOM      5  CG  PRO A  22      123.659  17.551 208.106
ATOM      6  CD  PRO A  22      122.582  17.607 207.019
ATOM      0  N   ASP A  23      120.496  14.625 210.023
ATOM      1  CA  ASP A  23      119.276  14.541 210.817
ATOM      2  C   ASP A  23      119.950  15.107 212.143
ATOM      3  O   ASP A  23      120.611  14.409 212.928
ATOM      4  H   ASP A  23      121.136  13.887 210.056
ATOM      5  CB  ASP A  23      118.755  13.100 210.973
ATOM      6  CG  ASP A  23      118.050  12.610 209.719
ATOM      7  OD1 ASP A  23      117.430  13.410 209.003
ATOM      8  OD2 ASP A  23      118.095  11.409 209.412
ATOM      0  N   ASN A  24      119.760  16.406 212.287
ATOM      1  CA  ASN A  24      120.404  17.176 213.350
ATOM      2  C   ASN A  24      120.953  16.626 214.646
ATOM      3  O   ASN A  24      120.705  17.203 215.709
ATOM      4  H   ASN A  24      119.168  16.869 211.663
ATOM      5  CB  ASN A  24      119.364  18.230 213.771
ATOM      6  CG  ASN A  24      119.059  19.209 212.649
ATOM      7  OD1 ASN A  24      119.983  19.684 211.972
ATOM      8  ND2 ASN A  24      117.754  19.474 212.496
ATOM      0  N   THR A  25      121.730  15.552 214.558
ATOM      1  CA  THR A  25      122.399  14.946 215.710
ATOM      2  C   THR A  25      123.949  15.102 215.502
ATOM      3  O   THR A  25      124.346  14.995 214.341
ATOM      4  H   THR A  25      121.864  15.146 213.679
ATOM      5  CB  THR A  25      122.031  13.454 215.806
ATOM      6  OG1 THR A  25      120.622  13.309 216.003
ATOM      7  CG2 THR A  25      122.740  12.766 216.962
ATOM      0  N   ARG A  26      124.758  15.337 216.531
ATOM      1  CA  ARG A  26      126.199  15.386 216.364
ATOM      2  C   ARG A  26      126.666  13.935 216.370
ATOM      3  O   ARG A  26      125.869  13.023 216.633
ATOM      4  H   ARG A  26      124.375  15.481 217.419
ATOM      5  CB  ARG A  26      126.850  16.162 217.524
ATOM      6  CG  ARG A  26      128.356  16.280 217.356
ATOM      7  CD  ARG A  26      128.921  17.474 218.108
ATOM      8  NE  ARG A  26      130.357  17.658 217.851
ATOM      9  CZ  ARG A  26      131.023  18.674 218.440
ATOM     10  NH1 ARG A  26      132.332  18.841 218.206
ATOM     11  NH2 ARG A  26      130.376  19.515 219.258
TER
ENDMDL
MODEL        5
ATOM      0  N   ASP A   1      124.422  -9.255 216.179
ATOM      1  CA  ASP A   1      124.050  -9.969 214.969
ATOM      2  C   ASP A   1      123.820  -8.972 213.835
ATOM      3  O   ASP A   1      122.934  -8.125 213.912
ATOM      4  H   ASP A   1      124.614  -9.772 217.037
ATOM      5  CB  ASP A   1      122.756 -10.765 215.221
ATOM      6  CG  ASP A   1      122.982 -11.938 216.160
```

```
ATOM 7 OD1 ASP A 1 124.074 -12.527 216.165
ATOM 8 OD2 ASP A 1 122.076 -12.307 216.921
ATOM 0 N SER A 2 124.631 -9.062 212.787
ATOM 1 CA SER A 2 124.471 -8.188 211.634
ATOM 2 C SER A 2 124.007 -9.016 210.451
ATOM 3 O SER A 2 124.673 -9.969 210.045
ATOM 4 H SER A 2 125.347 -9.727 212.789
ATOM 5 CB SER A 2 125.818 -7.520 211.300
ATOM 6 OG SER A 2 126.733 -8.544 210.902
ATOM 0 N THR A 3 122.852 -8.664 209.908
ATOM 1 CA THR A 3 122.341 -9.380 208.760
ATOM 2 C THR A 3 122.583 -8.515 207.551
ATOM 3 O THR A 3 122.067 -7.402 207.450
ATOM 4 H THR A 3 122.344 -7.918 210.283
ATOM 5 CB THR A 3 120.832 -9.639 208.928
ATOM 6 OG1 THR A 3 120.601 -10.461 210.075
ATOM 7 CG2 THR A 3 120.237 -10.343 207.719
ATOM 0 N VAL A 4 123.359 -9.033 206.616
ATOM 1 CA VAL A 4 123.752 -8.227 205.486
ATOM 2 C VAL A 4 122.709 -7.521 204.684
ATOM 3 O VAL A 4 122.977 -6.386 204.273
ATOM 4 H VAL A 4 123.665 -9.958 206.688
ATOM 5 CB VAL A 4 124.509 -9.154 204.517
ATOM 6 CG1 VAL A 4 124.927 -8.430 203.247
ATOM 7 CG2 VAL A 4 125.772 -9.724 205.143
ATOM 0 N PHE A 5 121.513 -8.065 204.542
ATOM 1 CA PHE A 5 120.464 -7.409 203.767
ATOM 2 C PHE A 5 120.067 -6.265 204.753
ATOM 3 O PHE A 5 119.762 -5.200 204.250
ATOM 4 H PHE A 5 121.326 -8.926 204.965
ATOM 5 CB PHE A 5 119.282 -8.356 203.488
ATOM 6 CG PHE A 5 119.626 -9.403 202.441
ATOM 7 CD1 PHE A 5 120.106 -10.626 202.827
ATOM 8 CE1 PHE A 5 120.416 -11.570 201.883
ATOM 9 CZ PHE A 5 120.246 -11.289 200.553
ATOM 10 CE2 PHE A 5 119.766 -10.066 200.166
ATOM 11 CD2 PHE A 5 119.456 -9.122 201.110
ATOM 0 N GLU A 6 120.046 -6.392 206.081
ATOM 1 CA GLU A 6 119.724 -5.268 206.930
ATOM 2 C GLU A 6 120.696 -4.146 206.723
ATOM 3 O GLU A 6 120.381 -2.982 206.488
ATOM 4 H GLU A 6 120.251 -7.257 206.486
ATOM 5 CB GLU A 6 119.778 -5.714 208.402
ATOM 6 CG GLU A 6 118.703 -6.738 208.727
ATOM 7 CD GLU A 6 118.787 -7.215 210.168
ATOM 8 OE1 GLU A 6 117.913 -7.967 210.624
ATOM 9 OE2 GLU A 6 119.729 -6.852 210.887
ATOM 0 N ASN A 7 121.954 -4.520 206.733
ATOM 1 CA ASN A 7 122.963 -3.504 206.572
ATOM 2 C ASN A 7 122.933 -2.741 205.246
ATOM 3 O ASN A 7 123.068 -1.516 205.190
```

ATOM 4 H ASN A 7 122.200 -5.459 206.845
ATOM 5 CB ASN A 7 124.341 -4.180 206.696
ATOM 6 CG ASN A 7 124.617 -4.655 208.113
ATOM 7 OD1 ASN A 7 124.349 -3.921 209.076
ATOM 8 ND2 ASN A 7 125.149 -5.883 208.183
ATOM 0 N LEU A 8 122.726 -3.487 204.162
ATOM 1 CA LEU A 8 122.678 -2.863 202.844
ATOM 2 C LEU A 8 121.679 -1.769 202.601
ATOM 3 O LEU A 8 121.941 -0.662 202.104
ATOM 4 H LEU A 8 122.606 -4.453 204.249
ATOM 5 CB LEU A 8 122.385 -3.978 201.823
ATOM 6 CG LEU A 8 122.250 -3.435 200.409
ATOM 7 CD1 LEU A 8 123.537 -2.788 199.923
ATOM 8 CD2 LEU A 8 121.892 -4.525 199.412
ATOM 0 N PRO A 9 120.460 -2.130 202.975
ATOM 1 CA PRO A 9 119.339 -1.203 202.898
ATOM 2 C PRO A 9 119.572 0.029 203.744
ATOM 3 O PRO A 9 119.297 1.143 203.339
ATOM 4 CB PRO A 9 118.106 -1.974 203.403
ATOM 5 CG PRO A 9 118.501 -3.454 203.239
ATOM 6 CD PRO A 9 120.017 -3.439 203.451
ATOM 0 N ASP A 10 120.091 -0.115 204.964
ATOM 1 CA ASP A 10 120.386 1.036 205.787
ATOM 2 C ASP A 10 121.475 1.945 205.211
ATOM 3 O ASP A 10 121.337 3.170 205.235
ATOM 4 H ASP A 10 120.276 -1.011 205.307
ATOM 5 CB ASP A 10 120.850 0.540 207.169
ATOM 6 CG ASP A 10 119.723 -0.124 207.943
ATOM 7 OD1 ASP A 10 118.555 0.262 207.792
ATOM 8 OD2 ASP A 10 119.973 -1.053 208.726
ATOM 0 N GLU A 11 122.552 1.357 204.694
ATOM 1 CA GLU A 11 123.637 2.169 204.150
ATOM 2 C GLU A 11 123.056 2.859 202.905
ATOM 3 O GLU A 11 123.384 4.023 202.731
ATOM 4 H GLU A 11 122.617 0.382 204.677
ATOM 5 CB GLU A 11 124.831 1.279 203.760
ATOM 6 CG GLU A 11 125.501 0.660 204.976
ATOM 7 CD GLU A 11 126.650 -0.256 204.589
ATOM 8 OE1 GLU A 11 127.372 -0.752 205.468
ATOM 9 OE2 GLU A 11 126.866 -0.509 203.394
ATOM 0 N ALA A 12 122.207 2.255 202.093
ATOM 1 CA ALA A 12 121.640 3.004 200.957
ATOM 2 C ALA A 12 120.779 4.149 201.428
ATOM 3 O ALA A 12 120.852 5.234 200.873
ATOM 4 H ALA A 12 121.957 1.322 202.244
ATOM 5 CB ALA A 12 120.781 2.051 200.105
ATOM 0 N ASP A 13 119.999 3.964 202.478
ATOM 1 CA ASP A 13 119.204 5.094 202.992
ATOM 2 C ASP A 13 120.042 6.248 203.557
ATOM 3 O ASP A 13 119.744 7.417 203.301
ATOM 4 H ASP A 13 119.949 3.089 202.909

ATOM	5	CB	ASP	A	13	118.288	4.571	204.114
ATOM	6	CG	ASP	A	13	117.214	3.636	203.582
ATOM	7	OD1	ASP	A	13	116.752	3.805	202.444
ATOM	8	OD2	ASP	A	13	116.799	2.704	204.287
ATOM	0	N	ARG	A	14	121.090	5.927	204.314
ATOM	1	CA	ARG	A	14	121.918	6.978	204.896
ATOM	2	C	ARG	A	14	122.616	7.664	203.711
ATOM	3	O	ARG	A	14	122.715	8.879	203.771
ATOM	4	H	ARG	A	14	121.304	4.989	204.480
ATOM	5	CB	ARG	A	14	122.961	6.373	205.854
ATOM	6	CG	ARG	A	14	123.810	7.442	206.522
ATOM	7	CD	ARG	A	14	124.401	6.962	207.837
ATOM	8	NE	ARG	A	14	125.124	8.032	208.540
ATOM	9	CZ	ARG	A	14	125.713	7.781	209.728
ATOM	10	NH1	ARG	A	14	126.372	8.756	210.368
ATOM	11	NH2	ARG	A	14	125.639	6.556	210.267
ATOM	0	N	ASP	A	15	123.040	6.991	202.656
ATOM	1	CA	ASP	A	15	123.640	7.725	201.526
ATOM	2	C	ASP	A	15	122.669	8.662	200.798
ATOM	3	O	ASP	A	15	123.029	9.792	200.461
ATOM	4	H	ASP	A	15	122.958	6.017	202.624
ATOM	5	CB	ASP	A	15	124.172	6.698	200.509
ATOM	6	CG	ASP	A	15	125.357	5.919	201.054
ATOM	7	OD1	ASP	A	15	126.143	6.459	201.846
ATOM	8	OD2	ASP	A	15	125.539	4.742	200.706
ATOM	0	N	LYS	A	16	121.440	8.204	200.562
ATOM	1	CA	LYS	A	16	120.472	9.039	199.858
ATOM	2	C	LYS	A	16	120.103	10.248	200.771
ATOM	3	O	LYS	A	16	119.984	11.344	200.217
ATOM	4	H	LYS	A	16	121.186	7.309	200.860
ATOM	5	CB	LYS	A	16	119.206	8.220	199.547
ATOM	6	CG	LYS	A	16	119.456	7.170	198.476
ATOM	7	CD	LYS	A	16	118.201	6.378	198.151
ATOM	8	CE	LYS	A	16	118.451	5.328	197.080
ATOM	9	NZ	LYS	A	16	117.228	4.557	196.764
ATOM	0	N	TYR	A	17	119.930	10.088	202.082
ATOM	1	CA	TYR	A	17	119.561	11.229	202.915
ATOM	2	C	TYR	A	17	120.777	12.170	202.895
ATOM	3	O	TYR	A	17	120.534	13.365	202.825
ATOM	4	H	TYR	A	17	120.051	9.207	202.487
ATOM	5	CB	TYR	A	17	119.263	10.770	204.354
ATOM	6	CG	TYR	A	17	118.934	11.939	205.268
ATOM	7	CD1	TYR	A	17	117.695	12.521	205.216
ATOM	8	CE1	TYR	A	17	117.398	13.575	206.040
ATOM	9	CZ	TYR	A	17	118.340	14.047	206.915
ATOM	10	CE2	TYR	A	17	119.580	13.466	206.966
ATOM	11	CD2	TYR	A	17	119.876	12.412	206.143
ATOM	12	OH	TYR	A	17	118.041	15.109	207.744
ATOM	0	N	GLU	A	18	122.019	11.720	202.897
ATOM	1	CA	GLU	A	18	123.133	12.681	202.808
ATOM	2	C	GLU	A	18	123.124	13.412	201.490

ATOM	3	O	GLU	A	18	123.344	14.613	201.457
ATOM	4	H	GLU	A	18	122.197	10.761	202.956
ATOM	5	CB	GLU	A	18	124.465	11.920	202.949
ATOM	6	CG	GLU	A	18	124.622	11.296	204.325
ATOM	7	CD	GLU	A	18	125.914	10.504	204.448
ATOM	8	OE1	GLU	A	18	126.245	10.025	205.543
ATOM	9	OE2	GLU	A	18	126.636	10.338	203.454
ATOM	0	N	LEU	A	19	122.820	12.739	200.394
ATOM	1	CA	LEU	A	19	122.739	13.456	199.108
ATOM	2	C	LEU	A	19	121.647	14.469	198.842
ATOM	3	O	LEU	A	19	121.858	15.565	198.322
ATOM	4	H	LEU	A	19	122.649	11.778	200.435
ATOM	5	CB	LEU	A	19	122.609	12.379	198.014
ATOM	6	CG	LEU	A	19	123.827	11.471	197.962
ATOM	7	CD1	LEU	A	19	123.673	10.371	196.924
ATOM	8	CD2	LEU	A	19	125.094	12.237	197.618
ATOM	0	N	LEU	A	20	120.440	14.079	199.238
ATOM	1	CA	LEU	A	20	119.268	14.912	199.006
ATOM	2	C	LEU	A	20	119.239	15.831	200.153
ATOM	3	O	LEU	A	20	118.820	16.962	199.912
ATOM	4	H	LEU	A	20	120.336	13.220	199.692
ATOM	5	CB	LEU	A	20	117.988	14.056	198.965
ATOM	6	CG	LEU	A	20	117.988	13.089	197.792
ATOM	7	CD1	LEU	A	20	116.854	12.082	197.885
ATOM	8	CD2	LEU	A	20	117.841	13.808	196.461
ATOM	0	N	CYS	A	21	119.605	15.445	201.377
ATOM	1	CA	CYS	A	21	119.520	16.368	202.508
ATOM	2	C	CYS	A	21	120.745	16.442	203.380
ATOM	3	O	CYS	A	21	121.444	15.442	203.516
ATOM	4	H	CYS	A	21	119.934	14.535	201.520
ATOM	5	CB	CYS	A	21	118.336	15.930	203.391
ATOM	6	SG	CYS	A	21	116.708	15.994	202.581
ATOM	0	N	PRO	A	22	121.034	17.607	203.945
ATOM	1	CA	PRO	A	22	122.240	17.833	204.751
ATOM	2	C	PRO	A	22	121.924	17.918	206.158
ATOM	3	O	PRO	A	22	122.445	18.902	206.671
ATOM	4	CB	PRO	A	22	122.851	19.158	204.256
ATOM	5	CG	PRO	A	22	122.257	19.335	202.845
ATOM	6	CD	PRO	A	22	120.883	18.669	202.956
ATOM	0	N	ASP	A	23	121.155	17.077	206.830
ATOM	1	CA	ASP	A	23	120.831	17.274	208.235
ATOM	2	C	ASP	A	23	120.967	15.926	209.023
ATOM	3	O	ASP	A	23	120.698	14.894	208.444
ATOM	4	H	ASP	A	23	120.792	16.296	206.368
ATOM	5	CB	ASP	A	23	119.383	17.785	208.356
ATOM	6	CG	ASP	A	23	119.237	19.207	207.839
ATOM	7	OD1	ASP	A	23	120.179	20.006	207.945
ATOM	8	OD2	ASP	A	23	118.175	19.568	207.311
ATOM	0	N	ASN	A	24	121.333	16.013	210.286
ATOM	1	CA	ASN	A	24	121.339	14.844	211.178
ATOM	2	C	ASN	A	24	120.469	13.839	210.755

```
ATOM      3  O    ASN A  24       120.552  13.267 211.845
ATOM      4  H    ASN A  24       121.607  16.881 210.642
ATOM      5  CB   ASN A  24       120.919  15.296 212.589
ATOM      6  CG   ASN A  24       121.991  16.141 213.258
ATOM      7  OD1 ASN A  24       123.181  15.797 213.194
ATOM      8  ND2 ASN A  24       121.518  17.227 213.883
ATOM      0  N    THR A  25       119.473  13.640 209.897
ATOM      1  CA   THR A  25       118.376  12.703 210.141
ATOM      2  C    THR A  25       118.608  11.290 209.759
ATOM      3  O    THR A  25       119.240  10.940 208.787
ATOM      4  H    THR A  25       119.474  14.145 209.060
ATOM      5  CB   THR A  25       117.150  13.202 209.353
ATOM      6  OG1 THR A  25       116.817  14.532 209.760
ATOM      7  CG2 THR A  25       115.931  12.323 209.580
ATOM      0  N    ARG A  26       118.033  10.376 210.566
ATOM      1  CA   ARG A  26       118.206   8.959 210.365
ATOM      2  C    ARG A  26       117.135   8.370 209.463
ATOM      3  O    ARG A  26       115.952   8.548 209.733
ATOM      4  H    ARG A  26       117.481  10.683 211.312
ATOM      5  CB   ARG A  26       118.144   8.252 211.732
ATOM      6  CG   ARG A  26       118.444   6.766 211.618
ATOM      7  CD   ARG A  26       118.955   6.187 212.927
ATOM      8  NE   ARG A  26       119.356   4.780 212.788
ATOM      9  CZ   ARG A  26       119.836   4.105 213.854
ATOM     10  NH1 ARG A  26       120.202   2.822 213.727
ATOM     11  NH2 ARG A  26       119.945   4.719 215.041
TER
ENDMDL
```

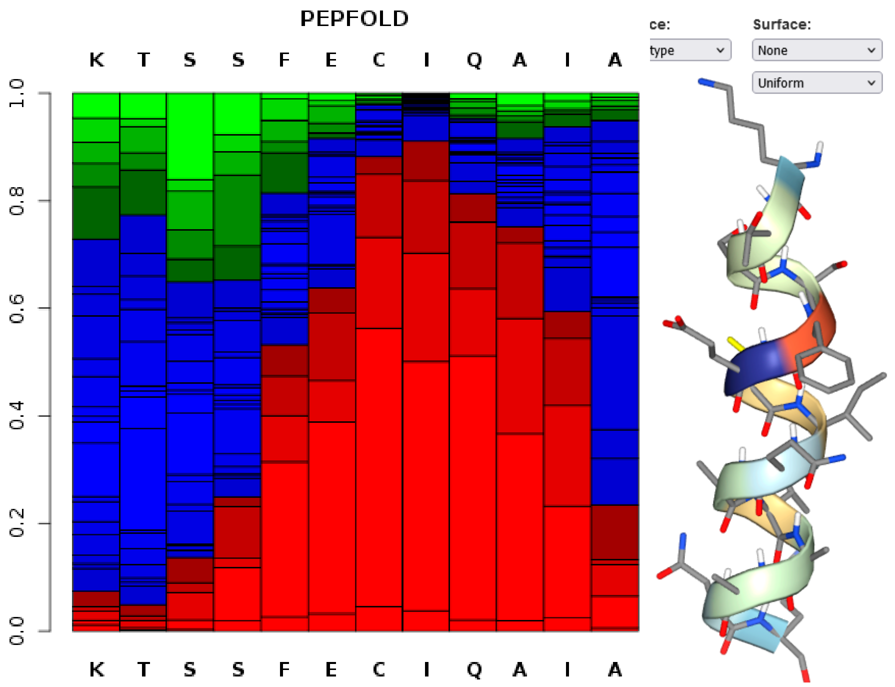

Figure S2 3D structure of peptide and predicted Local Structure Profile of KTSSFECIQAIA

job execution pep-fold2.py --autoFromTo --parallelposttreatment --maxSimSze 40 -s iSeq.fasta -l PEPFOLD --shortcombined --sortKey sOPEP

Table S3 Model 1 (*Protein* KTSSFECIQAIA *_3DStructure)*

```
CRYST1   68.471   68.471   68.471  90.00  90.00  90.00 P 1           1
MODEL        1
ATOM      1  N   LYS     1      37.960  42.810  33.240  1.00  0.00
ATOM      2  H1  LYS     1      38.060  42.990  32.260  1.00  0.00
ATOM      3  H2  LYS     1      37.040  42.360  33.260  1.00  0.00
ATOM      4  CA  LYS     1      39.020  41.870  33.690  1.00  0.00
ATOM      5  CB  LYS     1      40.390  42.280  33.090  1.00  0.00
ATOM      6  CG  LYS     1      40.650  43.770  32.830  1.00  0.00
ATOM      7  CD  LYS     1      41.460  44.560  33.870  1.00  0.00
ATOM      8  CE  LYS     1      40.660  45.090  35.060  1.00  0.00
ATOM      9  NZ  LYS     1      40.610  44.100  36.140  1.00  0.00
ATOM     10  HZ1 LYS     1      39.840  44.280  36.770  1.00  0.00
ATOM     11  HZ2 LYS     1      40.470  43.180  35.770  1.00  0.00
ATOM     12  HZ3 LYS     1      41.460  44.100  36.660  1.00  0.00
ATOM     13  C   LYS     1      39.100  41.530  35.200  1.00  0.00
ATOM     14  O   LYS     1      40.200  41.380  35.730  1.00  0.00
ATOM     15  N   THR     2      37.940  41.580  35.850  1.00  0.00
ATOM     16  H   THR     2      37.100  41.570  35.280  1.00  0.00
ATOM     17  CA  THR     2      37.670  41.380  37.310  1.00  0.00
ATOM     18  CB  THR     2      38.430  42.280  38.310  1.00  0.00
ATOM     19  OG1 THR     2      38.440  43.640  37.850  1.00  0.00
ATOM     20  HG1 THR     2      37.450  43.920  37.860  1.00  0.00
ATOM     21  CG2 THR     2      39.800  41.740  38.730  1.00  0.00
ATOM     22  C   THR     2      36.190  41.600  37.630  1.00  0.00
ATOM     23  O   THR     2      35.770  42.720  37.950  1.00  0.00
ATOM     24  N   SER     3      35.410  40.530  37.530  1.00  0.00
ATOM     25  H   SER     3      35.690  39.750  36.960  1.00  0.00
ATOM     26  CA  SER     3      33.970  40.510  37.900  1.00  0.00
ATOM     27  CB  SER     3      33.090  41.190  36.840  1.00  0.00
ATOM     28  OG  SER     3      33.050  40.420  35.630  1.00  0.00
ATOM     29  HG  SER     3      33.890  40.690  35.090  1.00  0.00
ATOM     30  C   SER     3      33.460  39.080  38.060  1.00  0.00
ATOM     31  O   SER     3      34.010  38.140  37.480  1.00  0.00
ATOM     32  N   SER     4      32.330  38.960  38.760  1.00  0.00
ATOM     33  H   SER     4      31.860  39.760  39.140  1.00  0.00
ATOM     34  CA  SER     4      31.600  37.680  38.940  1.00  0.00
ATOM     35  CB  SER     4      30.310  37.900  39.750  1.00  0.00
ATOM     36  OG  SER     4      29.600  39.010  39.190  1.00  0.00
```

ATOM	37	HG	SER	4	28.720	39.120	39.670	1.00	0.00
ATOM	38	C	SER	4	31.220	37.030	37.600	1.00	0.00
ATOM	39	O	SER	4	31.600	35.880	37.360	1.00	0.00
ATOM	40	N	PHE	5	30.710	37.860	36.690	1.00	0.00
ATOM	41	H	PHE	5	30.410	38.770	36.980	1.00	0.00
ATOM	42	CA	PHE	5	30.380	37.530	35.280	1.00	0.00
ATOM	43	CB	PHE	5	29.930	38.850	34.620	1.00	0.00
ATOM	44	CG	PHE	5	29.400	38.740	33.190	1.00	0.00
ATOM	45	CD1	PHE	5	28.050	38.460	32.980	1.00	0.00
ATOM	46	HD1	PHE	5	27.440	38.080	33.800	1.00	0.00
ATOM	47	CD2	PHE	5	30.170	39.220	32.140	1.00	0.00
ATOM	48	HD2	PHE	5	31.240	39.360	32.270	1.00	0.00
ATOM	49	CE1	PHE	5	27.470	38.690	31.740	1.00	0.00
ATOM	50	HE1	PHE	5	26.400	38.530	31.600	1.00	0.00
ATOM	51	CE2	PHE	5	29.590	39.460	30.900	1.00	0.00
ATOM	52	HE2	PHE	5	30.200	39.870	30.090	1.00	0.00
ATOM	53	CZ	PHE	5	28.240	39.200	30.700	1.00	0.00
ATOM	54	HZ	PHE	5	27.770	39.460	29.740	1.00	0.00
ATOM	55	C	PHE	5	31.570	36.880	34.540	1.00	0.00
ATOM	56	O	PHE	5	31.560	35.660	34.350	1.00	0.00
ATOM	57	N	GLU	6	32.680	37.610	34.460	1.00	0.00
ATOM	58	H	GLU	6	32.700	38.570	34.770	1.00	0.00
ATOM	59	CA	GLU	6	33.940	37.140	33.830	1.00	0.00
ATOM	60	CB	GLU	6	34.980	38.260	33.840	1.00	0.00
ATOM	61	CG	GLU	6	34.550	39.420	32.940	1.00	0.00
ATOM	62	CD	GLU	6	35.210	40.750	33.330	1.00	0.00
ATOM	63	OE1	GLU	6	35.360	41.610	32.450	1.00	0.00
ATOM	64	OE2	GLU	6	35.490	40.960	34.530	1.00	0.00
ATOM	65	C	GLU	6	34.520	35.870	34.470	1.00	0.00
ATOM	66	O	GLU	6	34.770	34.890	33.770	1.00	0.00
ATOM	67	N	CYS	7	34.550	35.830	35.800	1.00	0.00
ATOM	68	H	CYS	7	34.360	36.650	36.350	1.00	0.00
ATOM	69	CA	CYS	7	34.990	34.630	36.560	1.00	0.00
ATOM	70	CB	CYS	7	35.030	34.900	38.070	1.00	0.00
ATOM	71	SG	CYS	7	36.250	36.170	38.550	1.00	0.00
ATOM	72	HG	CYS	7	36.060	36.070	39.860	1.00	0.00
ATOM	73	C	CYS	7	34.160	33.370	36.300	1.00	0.00
ATOM	74	O	CYS	7	34.720	32.340	35.910	1.00	0.00
ATOM	75	N	ILE	8	32.830	33.490	36.340	1.00	0.00
ATOM	76	H	ILE	8	32.380	34.360	36.580	1.00	0.00
ATOM	77	CA	ILE	8	31.900	32.370	36.030	1.00	0.00
ATOM	78	CB	ILE	8	30.450	32.720	36.410	1.00	0.00
ATOM	79	CG1	ILE	8	30.350	32.950	37.920	1.00	0.00

ATOM	80	CG2	ILE	8	29.460	31.620	35.990	1.00	0.00
ATOM	81	CD	ILE	8	29.030	33.620	38.330	1.00	0.00
ATOM	82	C	ILE	8	32.040	31.900	34.570	1.00	0.00
ATOM	83	O	ILE	8	32.250	30.710	34.340	1.00	0.00
ATOM	84	N	GLN	9	32.120	32.850	33.630	1.00	0.00
ATOM	85	H	GLN	9	31.980	33.820	33.850	1.00	0.00
ATOM	86	CA	GLN	9	32.360	32.560	32.200	1.00	0.00
ATOM	87	CB	GLN	9	32.250	33.840	31.380	1.00	0.00
ATOM	88	CG	GLN	9	30.810	34.360	31.400	1.00	0.00
ATOM	89	CD	GLN	9	30.710	35.840	31.050	1.00	0.00
ATOM	90	OE1	GLN	9	31.640	36.630	31.150	1.00	0.00
ATOM	91	NE2	GLN	9	29.530	36.290	30.690	1.00	0.00
ATOM	92	HE21	GLN	9	28.750	35.700	30.600	1.00	0.00
ATOM	93	HE22	GLN	9	29.450	37.290	30.670	1.00	0.00
ATOM	94	C	GLN	9	33.710	31.870	31.930	1.00	0.00
ATOM	95	O	GLN	9	33.740	30.790	31.330	1.00	0.00
ATOM	96	N	ALA	10	34.770	32.350	32.590	1.00	0.00
ATOM	97	H	ALA	10	34.740	33.260	33.020	1.00	0.00
ATOM	98	CA	ALA	10	36.110	31.710	32.570	1.00	0.00
ATOM	99	CB	ALA	10	37.100	32.550	33.360	1.00	0.00
ATOM	100	C	ALA	10	36.110	30.260	33.090	1.00	0.00
ATOM	101	O	ALA	10	36.650	29.370	32.430	1.00	0.00
ATOM	102	N	ILE	11	35.460	30.020	34.230	1.00	0.00
ATOM	103	H	ILE	11	35.130	30.770	34.810	1.00	0.00
ATOM	104	CA	ILE	11	35.280	28.650	34.790	1.00	0.00
ATOM	105	CB	ILE	11	34.750	28.730	36.240	1.00	0.00
ATOM	106	CG1	ILE	11	35.760	29.490	37.120	1.00	0.00
ATOM	107	CG2	ILE	11	34.500	27.340	36.840	1.00	0.00
ATOM	108	CD	ILE	11	35.160	29.900	38.470	1.00	0.00
ATOM	109	C	ILE	11	34.410	27.750	33.890	1.00	0.00
ATOM	110	O	ILE	11	34.730	26.580	33.690	1.00	0.00
ATOM	111	N	ALA	12	33.320	28.300	33.350	1.00	0.00
ATOM	112	H	ALA	12	33.000	29.210	33.640	1.00	0.00
ATOM	113	CA	ALA	12	32.430	27.600	32.390	1.00	0.00
ATOM	114	CB	ALA	12	31.200	28.460	32.100	1.00	0.00
ATOM	115	C	ALA	12	33.120	27.230	31.060	1.00	0.00
ATOM	116	O	ALA	12	32.800	26.190	30.480	1.00	0.00
ATOM	117	N	ALA	13	34.000	28.110	30.580	1.00	0.00
ATOM	118	H	ALA	13	33.960	29.070	30.860	1.00	0.00
ATOM	119	CA	ALA	13	34.900	27.840	29.430	1.00	0.00
ATOM	120	CB	ALA	13	35.580	29.150	29.000	1.00	0.00
ATOM	121	C	ALA	13	35.960	26.760	29.710	1.00	0.00
ATOM	122	O	ALA	13	35.920	25.710	29.080	1.00	0.00

```
ATOM    123  N   ASN    14      36.770  26.960  30.750  1.00  0.00
ATOM    124  H   ASN    14      36.730  27.810  31.280  1.00  0.00
ATOM    125  CA  ASN    14      37.840  26.010  31.170  1.00  0.00
ATOM    126  CB  ASN    14      38.650  26.590  32.340  1.00  0.00
ATOM    127  CG  ASN    14      39.690  27.620  31.880  1.00  0.00
ATOM    128  OD1 ASN    14      40.840  27.320  31.590  1.00  0.00
ATOM    129  ND2 ASN    14      39.330  28.880  31.840  1.00  0.00
ATOM    130 HD21 ASN    14      38.410  29.180  32.100  1.00  0.00
ATOM    131 HD22 ASN    14      40.040  29.500  31.530  1.00  0.00
ATOM    132  C   ASN    14      37.350  24.590  31.530  1.00  0.00
ATOM    133  O   ASN    14      38.090  23.620  31.420  1.00  0.00
ATOM    134  N   LYS    15      36.190  24.540  32.180  1.00  0.00
ATOM    135  H   LYS    15      35.810  25.360  32.630  1.00  0.00
ATOM    136  CA  LYS    15      35.390  23.310  32.300  1.00  0.00
ATOM    137  CB  LYS    15      35.320  22.920  33.780  1.00  0.00
ATOM    138  CG  LYS    15      34.740  21.510  33.970  1.00  0.00
ATOM    139  CD  LYS    15      34.080  21.360  35.350  1.00  0.00
ATOM    140  CE  LYS    15      32.600  21.770  35.370  1.00  0.00
ATOM    141  NZ  LYS    15      32.360  23.180  35.040  1.00  0.00
ATOM    142  HZ1 LYS    15      31.380  23.380  35.110  1.00  0.00
ATOM    143  HZ2 LYS    15      32.880  23.770  35.650  1.00  0.00
ATOM    144  HZ3 LYS    15      32.650  23.360  34.090  1.00  0.00
ATOM    145  C   LYS    15      34.010  23.670  31.720  1.00  0.00
ATOM    146  OT  LYS    15      33.040  23.880  32.460  1.00  0.00
ATOM    147  O   LYS    15      33.850  23.750  30.390  1.00  0.00
ATOM    148  HO  LYS    15      33.160  24.470  30.240  1.00  0.00
TER
ENDMDL
```

Model 2 ***(Protein*** **KTSSFECIQAIA** ***_3DStructure)***

```
CRYST1   65.135   65.135   65.135  90.00  90.00  90.00 P 1           1
MODEL        1
ATOM      1  N   LYS     1      28.120  28.650  40.580  1.00  0.00
ATOM      2  H1  LYS     1      28.600  27.900  41.050  1.00  0.00
ATOM      3  H2  LYS     1      28.760  29.430  40.660  1.00  0.00
ATOM      4  CA  LYS     1      28.030  28.330  39.130  1.00  0.00
ATOM      5  CB  LYS     1      28.030  26.840  38.810  1.00  0.00
ATOM      6  CG  LYS     1      29.490  26.360  38.860  1.00  0.00
ATOM      7  CD  LYS     1      30.220  26.120  37.520  1.00  0.00
ATOM      8  CE  LYS     1      30.190  27.190  36.410  1.00  0.00
ATOM      9  NZ  LYS     1      30.340  28.580  36.860  1.00  0.00
ATOM     10  HZ1 LYS     1      30.560  29.180  36.090  1.00  0.00
ATOM     11  HZ2 LYS     1      29.460  28.880  37.230  1.00  0.00
```

ATOM	12	HZ3	LYS	1	31.010	28.670	37.610	1.00	0.00
ATOM	13	C	LYS	1	27.160	29.160	38.180	1.00	0.00
ATOM	14	O	LYS	1	27.520	29.310	37.010	1.00	0.00
ATOM	15	N	THR	2	26.260	29.910	38.790	1.00	0.00
ATOM	16	H	THR	2	26.160	29.870	39.790	1.00	0.00
ATOM	17	CA	THR	2	25.380	30.890	38.110	1.00	0.00
ATOM	18	CB	THR	2	24.170	31.260	38.980	1.00	0.00
ATOM	19	OG1	THR	2	24.620	31.600	40.290	1.00	0.00
ATOM	20	HG1	THR	2	23.840	31.930	40.830	1.00	0.00
ATOM	21	CG2	THR	2	23.150	30.120	39.010	1.00	0.00
ATOM	22	C	THR	2	26.060	32.200	37.670	1.00	0.00
ATOM	23	O	THR	2	25.540	32.880	36.780	1.00	0.00
ATOM	24	N	SER	3	27.200	32.560	38.260	1.00	0.00
ATOM	25	H	SER	3	27.780	31.900	38.750	1.00	0.00
ATOM	26	CA	SER	3	27.790	33.910	38.070	1.00	0.00
ATOM	27	CB	SER	3	28.750	34.330	39.190	1.00	0.00
ATOM	28	OG	SER	3	30.070	33.820	39.000	1.00	0.00
ATOM	29	HG	SER	3	30.050	32.820	39.260	1.00	0.00
ATOM	30	C	SER	3	28.480	34.130	36.720	1.00	0.00
ATOM	31	O	SER	3	29.220	33.300	36.190	1.00	0.00
ATOM	32	N	SER	4	28.320	35.370	36.260	1.00	0.00
ATOM	33	H	SER	4	27.680	36.000	36.700	1.00	0.00
ATOM	34	CA	SER	4	29.050	35.950	35.110	1.00	0.00
ATOM	35	CB	SER	4	28.630	37.400	34.890	1.00	0.00
ATOM	36	OG	SER	4	28.710	38.110	36.130	1.00	0.00
ATOM	37	HG	SER	4	28.660	39.100	35.970	1.00	0.00
ATOM	38	C	SER	4	30.580	35.900	35.330	1.00	0.00
ATOM	39	O	SER	4	31.280	35.280	34.540	1.00	0.00
ATOM	40	N	PHE	5	31.010	36.370	36.500	1.00	0.00
ATOM	41	H	PHE	5	30.390	36.930	37.050	1.00	0.00
ATOM	42	CA	PHE	5	32.420	36.390	36.950	1.00	0.00
ATOM	43	CB	PHE	5	32.500	36.960	38.380	1.00	0.00
ATOM	44	CG	PHE	5	31.930	38.380	38.460	1.00	0.00
ATOM	45	CD1	PHE	5	30.630	38.570	38.920	1.00	0.00
ATOM	46	HD1	PHE	5	30.110	37.760	39.430	1.00	0.00
ATOM	47	CD2	PHE	5	32.630	39.450	37.920	1.00	0.00
ATOM	48	HD2	PHE	5	33.680	39.320	37.620	1.00	0.00
ATOM	49	CE1	PHE	5	30.020	39.820	38.800	1.00	0.00
ATOM	50	HE1	PHE	5	29.010	39.970	39.170	1.00	0.00
ATOM	51	CE2	PHE	5	32.030	40.700	37.810	1.00	0.00
ATOM	52	HE2	PHE	5	32.590	41.540	37.400	1.00	0.00
ATOM	53	CZ	PHE	5	30.720	40.880	38.240	1.00	0.00
ATOM	54	HZ	PHE	5	30.260	41.860	38.170	1.00	0.00

ATOM	55	C	PHE	5	33.180	35.060	36.870	1.00	0.00
ATOM	56	O	PHE	5	34.350	35.050	36.470	1.00	0.00
ATOM	57	N	GLU	6	32.530	33.950	37.230	1.00	0.00
ATOM	58	H	GLU	6	31.720	33.980	37.830	1.00	0.00
ATOM	59	CA	GLU	6	33.050	32.600	36.920	1.00	0.00
ATOM	60	CB	GLU	6	32.240	31.500	37.600	1.00	0.00
ATOM	61	CG	GLU	6	32.200	31.520	39.120	1.00	0.00
ATOM	62	CD	GLU	6	31.010	30.650	39.530	1.00	0.00
ATOM	63	OE1	GLU	6	31.210	29.430	39.620	1.00	0.00
ATOM	64	OE2	GLU	6	29.880	31.180	39.510	1.00	0.00
ATOM	65	C	GLU	6	33.010	32.320	35.400	1.00	0.00
ATOM	66	O	GLU	6	34.030	32.300	34.710	1.00	0.00
ATOM	67	N	CYS	7	31.790	32.280	34.870	1.00	0.00
ATOM	68	H	CYS	7	30.990	32.630	35.380	1.00	0.00
ATOM	69	CA	CYS	7	31.500	31.780	33.510	1.00	0.00
ATOM	70	CB	CYS	7	29.990	31.620	33.330	1.00	0.00
ATOM	71	SG	CYS	7	29.340	30.360	34.490	1.00	0.00
ATOM	72	HG	CYS	7	28.050	30.650	34.310	1.00	0.00
ATOM	73	C	CYS	7	32.140	32.510	32.330	1.00	0.00
ATOM	74	O	CYS	7	32.530	31.840	31.370	1.00	0.00
ATOM	75	N	ILE	8	32.420	33.810	32.460	1.00	0.00
ATOM	76	H	ILE	8	32.080	34.350	33.250	1.00	0.00
ATOM	77	CA	ILE	8	33.160	34.590	31.430	1.00	0.00
ATOM	78	CB	ILE	8	33.240	36.100	31.720	1.00	0.00
ATOM	79	CG1	ILE	8	33.920	36.410	33.060	1.00	0.00
ATOM	80	CG2	ILE	8	31.850	36.720	31.590	1.00	0.00
ATOM	81	CD	ILE	8	34.180	37.900	33.270	1.00	0.00
ATOM	82	C	ILE	8	34.560	34.030	31.080	1.00	0.00
ATOM	83	O	ILE	8	34.880	33.930	29.900	1.00	0.00
ATOM	84	N	GLN	9	35.250	33.480	32.080	1.00	0.00
ATOM	85	H	GLN	9	34.880	33.470	33.020	1.00	0.00
ATOM	86	CA	GLN	9	36.580	32.850	31.930	1.00	0.00
ATOM	87	CB	GLN	9	37.110	32.440	33.310	1.00	0.00
ATOM	88	CG	GLN	9	37.200	33.630	34.280	1.00	0.00
ATOM	89	CD	GLN	9	37.540	33.170	35.700	1.00	0.00
ATOM	90	OE1	GLN	9	38.470	32.430	35.960	1.00	0.00
ATOM	91	NE2	GLN	9	36.770	33.610	36.670	1.00	0.00
ATOM	92	HE21	GLN	9	35.970	34.190	36.500	1.00	0.00
ATOM	93	HE22	GLN	9	37.040	33.310	37.580	1.00	0.00
ATOM	94	C	GLN	9	36.510	31.610	31.010	1.00	0.00
ATOM	95	O	GLN	9	37.150	31.580	29.960	1.00	0.00
ATOM	96	N	ALA	10	35.540	30.750	31.310	1.00	0.00
ATOM	97	H	ALA	10	35.020	30.850	32.160	1.00	0.00

ATOM	98	CA	ALA	10	35.190	29.560	30.500	1.00	0.00
ATOM	99	CB	ALA	10	34.080	28.780	31.190	1.00	0.00
ATOM	100	C	ALA	10	34.770	29.920	29.060	1.00	0.00
ATOM	101	O	ALA	10	35.490	29.570	28.130	1.00	0.00
ATOM	102	N	ILE	11	33.830	30.860	28.930	1.00	0.00
ATOM	103	H	ILE	11	33.350	31.230	29.730	1.00	0.00
ATOM	104	CA	ILE	11	33.360	31.370	27.610	1.00	0.00
ATOM	105	CB	ILE	11	32.160	32.330	27.800	1.00	0.00
ATOM	106	CG1	ILE	11	31.000	31.600	28.470	1.00	0.00
ATOM	107	CG2	ILE	11	31.690	32.930	26.470	1.00	0.00
ATOM	108	CD	ILE	11	29.920	32.550	28.990	1.00	0.00
ATOM	109	C	ILE	11	34.490	31.990	26.760	1.00	0.00
ATOM	110	O	ILE	11	34.590	31.680	25.570	1.00	0.00
ATOM	111	N	ALA	12	35.380	32.760	27.390	1.00	0.00
ATOM	112	H	ALA	12	35.240	33.050	28.340	1.00	0.00
ATOM	113	CA	ALA	12	36.590	33.310	26.730	1.00	0.00
ATOM	114	CB	ALA	12	37.340	34.220	27.700	1.00	0.00
ATOM	115	C	ALA	12	37.540	32.230	26.180	1.00	0.00
ATOM	116	O	ALA	12	37.960	32.330	25.030	1.00	0.00
ATOM	117	N	ALA	13	37.790	31.200	26.980	1.00	0.00
ATOM	118	H	ALA	13	37.500	31.200	27.940	1.00	0.00
ATOM	119	CA	ALA	13	38.580	30.010	26.580	1.00	0.00
ATOM	120	CB	ALA	13	38.910	29.180	27.820	1.00	0.00
ATOM	121	C	ALA	13	37.880	29.120	25.530	1.00	0.00
ATOM	122	O	ALA	13	38.530	28.650	24.600	1.00	0.00
ATOM	123	N	ASN	14	36.580	28.880	25.700	1.00	0.00
ATOM	124	H	ASN	14	36.140	29.090	26.580	1.00	0.00
ATOM	125	CA	ASN	14	35.730	28.150	24.720	1.00	0.00
ATOM	126	CB	ASN	14	34.310	27.940	25.280	1.00	0.00
ATOM	127	CG	ASN	14	34.250	26.830	26.330	1.00	0.00
ATOM	128	OD1	ASN	14	34.120	27.040	27.520	1.00	0.00
ATOM	129	ND2	ASN	14	34.250	25.600	25.880	1.00	0.00
ATOM	130	HD21	ASN	14	34.290	25.410	24.900	1.00	0.00
ATOM	131	HD22	ASN	14	34.200	24.880	26.570	1.00	0.00
ATOM	132	C	ASN	14	35.620	28.800	23.330	1.00	0.00
ATOM	133	O	ASN	14	35.280	28.120	22.360	1.00	0.00
ATOM	134	N	LYS	15	35.660	30.130	23.300	1.00	0.00
ATOM	135	H	LYS	15	35.550	30.660	24.150	1.00	0.00
ATOM	136	CA	LYS	15	35.750	30.940	22.070	1.00	0.00
ATOM	137	CB	LYS	15	35.360	32.360	22.470	1.00	0.00
ATOM	138	CG	LYS	15	34.980	33.250	21.280	1.00	0.00
ATOM	139	CD	LYS	15	35.130	34.710	21.700	1.00	0.00
ATOM	140	CE	LYS	15	35.060	35.630	20.480	1.00	0.00

```
ATOM    141  NZ  LYS    15      35.480  36.980  20.860  1.00  0.00
ATOM    142  HZ1 LYS    15      35.430  37.580  20.070  1.00  0.00
ATOM    143  HZ2 LYS    15      34.890  37.320  21.590  1.00  0.00
ATOM    144  HZ3 LYS    15      36.430  36.940  21.190  1.00  0.00
ATOM    145  C   LYS    15      37.170  30.880  21.460  1.00  0.00
ATOM    146  OT  LYS    15      37.640  31.800  20.790  1.00  0.00
ATOM    147  O   LYS    15      37.950  29.790  21.620  1.00  0.00
ATOM    148  HO  LYS    15      38.800  30.040  21.180  1.00  0.00
TER
ENDMDL
```

Model 3 (KTSSFECIQAIAANK *_3DStructure)*

```
CRYST1   66.539   66.539   66.539  90.00  90.00  90.00 P 1           1
MODEL        1
ATOM      1  N   LYS     1      32.160  29.240  42.560  1.00  0.00
ATOM      2  H1  LYS     1      32.630  28.510  43.070  1.00  0.00
ATOM      3  H2  LYS     1      32.510  30.130  42.930  1.00  0.00
ATOM      4  CA  LYS     1      32.420  29.110  41.110  1.00  0.00
ATOM      5  CB  LYS     1      31.820  27.790  40.610  1.00  0.00
ATOM      6  CG  LYS     1      32.200  27.460  39.160  1.00  0.00
ATOM      7  CD  LYS     1      31.690  26.070  38.790  1.00  0.00
ATOM      8  CE  LYS     1      32.020  25.680  37.340  1.00  0.00
ATOM      9  NZ  LYS     1      33.460  25.640  37.070  1.00  0.00
ATOM     10  HZ1 LYS     1      33.810  26.590  36.990  1.00  0.00
ATOM     11  HZ2 LYS     1      33.960  25.200  37.820  1.00  0.00
ATOM     12  HZ3 LYS     1      33.660  25.190  36.210  1.00  0.00
ATOM     13  C   LYS     1      31.910  30.330  40.300  1.00  0.00
ATOM     14  O   LYS     1      32.710  31.090  39.750  1.00  0.00
ATOM     15  N   THR     2      30.630  30.610  40.490  1.00  0.00
ATOM     16  H   THR     2      30.070  30.020  41.070  1.00  0.00
ATOM     17  CA  THR     2      29.940  31.830  40.000  1.00  0.00
ATOM     18  CB  THR     2      28.430  31.760  40.260  1.00  0.00
ATOM     19  OG1 THR     2      28.220  31.360  41.620  1.00  0.00
ATOM     20  HG1 THR     2      27.250  31.480  41.850  1.00  0.00
ATOM     21  CG2 THR     2      27.740  30.800  39.280  1.00  0.00
ATOM     22  C   THR     2      30.470  33.120  40.650  1.00  0.00
ATOM     23  O   THR     2      30.630  34.150  40.000  1.00  0.00
ATOM     24  N   SER     3      30.930  32.950  41.890  1.00  0.00
ATOM     25  H   SER     3      30.760  32.070  42.320  1.00  0.00
ATOM     26  CA  SER     3      31.680  33.890  42.760  1.00  0.00
ATOM     27  CB  SER     3      32.210  33.080  43.950  1.00  0.00
ATOM     28  OG  SER     3      32.870  31.880  43.490  1.00  0.00
ATOM     29  HG  SER     3      33.690  32.120  42.910  1.00  0.00
```

ATOM	30	C	SER	3	32.930	34.580	42.190	1.00	0.00
ATOM	31	O	SER	3	33.500	35.460	42.830	1.00	0.00
ATOM	32	N	SER	4	33.300	34.210	40.970	1.00	0.00
ATOM	33	H	SER	4	32.600	34.030	40.270	1.00	0.00
ATOM	34	CA	SER	4	34.710	34.230	40.510	1.00	0.00
ATOM	35	CB	SER	4	35.560	33.230	41.320	1.00	0.00
ATOM	36	OG	SER	4	34.810	32.030	41.570	1.00	0.00
ATOM	37	HG	SER	4	34.480	31.670	40.680	1.00	0.00
ATOM	38	C	SER	4	34.830	33.860	39.020	1.00	0.00
ATOM	39	O	SER	4	34.760	32.700	38.650	1.00	0.00
ATOM	40	N	PHE	5	34.970	34.900	38.200	1.00	0.00
ATOM	41	H	PHE	5	34.900	35.840	38.540	1.00	0.00
ATOM	42	CA	PHE	5	35.030	34.800	36.730	1.00	0.00
ATOM	43	CB	PHE	5	34.970	36.180	36.070	1.00	0.00
ATOM	44	CG	PHE	5	33.750	37.000	36.510	1.00	0.00
ATOM	45	CD1	PHE	5	33.940	38.110	37.320	1.00	0.00
ATOM	46	HD1	PHE	5	34.950	38.490	37.510	1.00	0.00
ATOM	47	CD2	PHE	5	32.460	36.570	36.210	1.00	0.00
ATOM	48	HD2	PHE	5	32.300	35.680	35.600	1.00	0.00
ATOM	49	CE1	PHE	5	32.850	38.780	37.850	1.00	0.00
ATOM	50	HE1	PHE	5	33.000	39.650	38.490	1.00	0.00
ATOM	51	CE2	PHE	5	31.370	37.250	36.730	1.00	0.00
ATOM	52	HE2	PHE	5	30.360	36.890	36.520	1.00	0.00
ATOM	53	CZ	PHE	5	31.560	38.350	37.550	1.00	0.00
ATOM	54	HZ	PHE	5	30.700	38.880	37.970	1.00	0.00
ATOM	55	C	PHE	5	36.200	33.980	36.160	1.00	0.00
ATOM	56	O	PHE	5	37.310	34.470	35.970	1.00	0.00
ATOM	57	N	GLU	6	35.940	32.690	36.030	1.00	0.00
ATOM	58	H	GLU	6	35.180	32.290	36.560	1.00	0.00
ATOM	59	CA	GLU	6	36.820	31.720	35.340	1.00	0.00
ATOM	60	CB	GLU	6	36.180	30.340	35.320	1.00	0.00
ATOM	61	CG	GLU	6	35.980	29.680	36.690	1.00	0.00
ATOM	62	CD	GLU	6	35.150	28.410	36.470	1.00	0.00
ATOM	63	OE1	GLU	6	33.910	28.520	36.450	1.00	0.00
ATOM	64	OE2	GLU	6	35.750	27.340	36.250	1.00	0.00
ATOM	65	C	GLU	6	37.030	32.090	33.860	1.00	0.00
ATOM	66	O	GLU	6	36.130	32.620	33.200	1.00	0.00
ATOM	67	N	CYS	7	38.120	31.560	33.300	1.00	0.00
ATOM	68	H	CYS	7	38.910	31.310	33.870	1.00	0.00
ATOM	69	CA	CYS	7	38.410	31.620	31.850	1.00	0.00
ATOM	70	CB	CYS	7	39.640	30.760	31.510	1.00	0.00
ATOM	71	SG	CYS	7	41.070	31.100	32.600	1.00	0.00
ATOM	72	HG	CYS	7	41.940	30.320	31.960	1.00	0.00

ATOM	73	C	CYS	7	37.240	31.140	30.980	1.00	0.00
ATOM	74	O	CYS	7	36.770	31.890	30.120	1.00	0.00
ATOM	75	N	ILE	8	36.620	30.020	31.380	1.00	0.00
ATOM	76	H	ILE	8	36.980	29.470	32.120	1.00	0.00
ATOM	77	CA	ILE	8	35.420	29.470	30.700	1.00	0.00
ATOM	78	CB	ILE	8	35.130	28.020	31.180	1.00	0.00
ATOM	79	CG1	ILE	8	34.010	27.360	30.370	1.00	0.00
ATOM	80	CG2	ILE	8	34.840	27.930	32.680	1.00	0.00
ATOM	81	CD	ILE	8	34.390	27.140	28.900	1.00	0.00
ATOM	82	C	ILE	8	34.180	30.400	30.760	1.00	0.00
ATOM	83	O	ILE	8	33.630	30.710	29.700	1.00	0.00
ATOM	84	N	GLN	9	33.930	31.020	31.920	1.00	0.00
ATOM	85	H	GLN	9	34.510	30.870	32.720	1.00	0.00
ATOM	86	CA	GLN	9	32.850	32.010	32.110	1.00	0.00
ATOM	87	CB	GLN	9	32.760	32.470	33.570	1.00	0.00
ATOM	88	CG	GLN	9	32.370	31.350	34.540	1.00	0.00
ATOM	89	CD	GLN	9	32.350	31.860	35.980	1.00	0.00
ATOM	90	OE1	GLN	9	31.980	32.980	36.290	1.00	0.00
ATOM	91	NE2	GLN	9	32.800	31.050	36.900	1.00	0.00
ATOM	92	HE21	GLN	9	33.160	30.120	36.680	1.00	0.00
ATOM	93	HE22	GLN	9	32.830	31.380	37.850	1.00	0.00
ATOM	94	C	GLN	9	33.040	33.250	31.220	1.00	0.00
ATOM	95	O	GLN	9	32.160	33.570	30.420	1.00	0.00
ATOM	96	N	ALA	10	34.270	33.770	31.200	1.00	0.00
ATOM	97	H	ALA	10	34.970	33.470	31.870	1.00	0.00
ATOM	98	CA	ALA	10	34.680	34.900	30.330	1.00	0.00
ATOM	99	CB	ALA	10	36.130	35.290	30.640	1.00	0.00
ATOM	100	C	ALA	10	34.510	34.610	28.830	1.00	0.00
ATOM	101	O	ALA	10	33.770	35.310	28.150	1.00	0.00
ATOM	102	N	ILE	11	35.040	33.470	28.370	1.00	0.00
ATOM	103	H	ILE	11	35.630	32.900	28.960	1.00	0.00
ATOM	104	CA	ILE	11	34.890	32.990	26.970	1.00	0.00
ATOM	105	CB	ILE	11	35.790	31.760	26.720	1.00	0.00
ATOM	106	CG1	ILE	11	37.260	32.150	26.920	1.00	0.00
ATOM	107	CG2	ILE	11	35.590	31.170	25.320	1.00	0.00
ATOM	108	CD	ILE	11	38.180	30.930	27.020	1.00	0.00
ATOM	109	C	ILE	11	33.410	32.740	26.580	1.00	0.00
ATOM	110	O	ILE	11	32.990	33.140	25.500	1.00	0.00
ATOM	111	N	ALA	12	32.630	32.160	27.490	1.00	0.00
ATOM	112	H	ALA	12	33.020	31.730	28.320	1.00	0.00
ATOM	113	CA	ALA	12	31.170	31.980	27.320	1.00	0.00
ATOM	114	CB	ALA	12	30.610	31.120	28.460	1.00	0.00
ATOM	115	C	ALA	12	30.390	33.310	27.240	1.00	0.00

ATOM 116 O ALA 12 29.750 33.560 26.220 1.00 0.00
ATOM 117 N ALA 13 30.640 34.210 28.190 1.00 0.00
ATOM 118 H ALA 13 31.240 33.990 28.970 1.00 0.00
ATOM 119 CA ALA 13 30.010 35.550 28.250 1.00 0.00
ATOM 120 CB ALA 13 30.220 36.150 29.650 1.00 0.00
ATOM 121 C ALA 13 30.430 36.530 27.140 1.00 0.00
ATOM 122 O ALA 13 29.810 36.550 26.080 1.00 0.00
ATOM 123 N ASN 14 31.630 37.100 27.270 1.00 0.00
ATOM 124 H ASN 14 32.280 36.780 27.960 1.00 0.00
ATOM 125 CA ASN 14 32.140 38.260 26.490 1.00 0.00
ATOM 126 CB ASN 14 33.620 38.500 26.810 1.00 0.00
ATOM 127 CG ASN 14 33.890 38.830 28.290 1.00 0.00
ATOM 128 OD1 ASN 14 33.330 38.290 29.220 1.00 0.00
ATOM 129 ND2 ASN 14 34.850 39.690 28.540 1.00 0.00
ATOM 130 HD21 ASN 14 35.390 40.110 27.820 1.00 0.00
ATOM 131 HD22 ASN 14 35.010 39.870 29.500 1.00 0.00
ATOM 132 C ASN 14 31.970 38.190 24.960 1.00 0.00
ATOM 133 O ASN 14 32.540 37.310 24.310 1.00 0.00
ATOM 134 N LYS 15 30.870 38.800 24.530 1.00 0.00
ATOM 135 H LYS 15 30.130 38.970 25.170 1.00 0.00
ATOM 136 CA LYS 15 30.540 39.090 23.110 1.00 0.00
ATOM 137 CB LYS 15 29.540 38.050 22.590 1.00 0.00
ATOM 138 CG LYS 15 30.290 36.800 22.120 1.00 0.00
ATOM 139 CD LYS 15 29.520 35.520 22.450 1.00 0.00
ATOM 140 CE LYS 15 30.510 34.420 22.850 1.00 0.00
ATOM 141 NZ LYS 15 31.190 34.780 24.100 1.00 0.00
ATOM 142 HZ1 LYS 15 31.870 34.080 24.360 1.00 0.00
ATOM 143 HZ2 LYS 15 31.650 35.670 24.020 1.00 0.00
ATOM 144 HZ3 LYS 15 30.510 34.830 24.840 1.00 0.00
ATOM 145 C LYS 15 29.990 40.520 22.910 1.00 0.00
ATOM 146 OT LYS 15 29.270 40.800 21.960 1.00 0.00
ATOM 147 O LYS 15 30.310 41.520 23.760 1.00 0.00
ATOM 148 HO LYS 15 29.830 42.290 23.350 1.00 0.00
TER
ENDMDL

Model 4 (KTSSFECIQAIAANK_*3DStructure)*
CRYST1 67.863 67.863 67.863 90.00 90.00 90.00 P 1 1
MODEL 1
ATOM 1 N LYS 1 33.060 26.460 45.740 1.00 0.00
ATOM 2 H1 LYS 1 33.840 25.830 45.780 1.00 0.00
ATOM 3 H2 LYS 1 33.270 27.270 46.300 1.00 0.00
ATOM 4 CA LYS 1 32.770 26.830 44.340 1.00 0.00

ATOM	5	CB	LYS	1	32.680	25.540	43.500	1.00	0.00
ATOM	6	CG	LYS	1	33.120	25.730	42.040	1.00	0.00
ATOM	7	CD	LYS	1	32.070	26.450	41.180	1.00	0.00
ATOM	8	CE	LYS	1	32.720	27.140	39.980	1.00	0.00
ATOM	9	NZ	LYS	1	31.800	28.150	39.450	1.00	0.00
ATOM	10	HZ1	LYS	1	32.350	28.930	39.110	1.00	0.00
ATOM	11	HZ2	LYS	1	31.240	27.800	38.710	1.00	0.00
ATOM	12	HZ3	LYS	1	31.220	28.510	40.190	1.00	0.00
ATOM	13	C	LYS	1	31.510	27.710	44.170	1.00	0.00
ATOM	14	O	LYS	1	30.370	27.270	44.300	1.00	0.00
ATOM	15	N	THR	2	31.770	28.960	43.800	1.00	0.00
ATOM	16	H	THR	2	32.720	29.270	43.670	1.00	0.00
ATOM	17	CA	THR	2	30.780	30.020	43.550	1.00	0.00
ATOM	18	CB	THR	2	31.460	31.340	43.130	1.00	0.00
ATOM	19	OG1	THR	2	32.890	31.180	43.070	1.00	0.00
ATOM	20	HG1	THR	2	33.160	30.970	42.090	1.00	0.00
ATOM	21	CG2	THR	2	31.080	32.470	44.090	1.00	0.00
ATOM	22	C	THR	2	29.760	29.680	42.460	1.00	0.00
ATOM	23	O	THR	2	30.100	29.160	41.400	1.00	0.00
ATOM	24	N	SER	3	28.500	30.030	42.720	1.00	0.00
ATOM	25	H	SER	3	28.240	30.410	43.610	1.00	0.00
ATOM	26	CA	SER	3	27.380	29.890	41.760	1.00	0.00
ATOM	27	CB	SER	3	26.040	30.190	42.450	1.00	0.00
ATOM	28	OG	SER	3	26.140	31.430	43.160	1.00	0.00
ATOM	29	HG	SER	3	25.230	31.680	43.520	1.00	0.00
ATOM	30	C	SER	3	27.520	30.810	40.530	1.00	0.00
ATOM	31	O	SER	3	27.370	30.360	39.400	1.00	0.00
ATOM	32	N	SER	4	27.750	32.090	40.800	1.00	0.00
ATOM	33	H	SER	4	27.460	32.470	41.680	1.00	0.00
ATOM	34	CA	SER	4	28.110	33.120	39.790	1.00	0.00
ATOM	35	CB	SER	4	28.410	34.450	40.480	1.00	0.00
ATOM	36	OG	SER	4	27.330	34.800	41.350	1.00	0.00
ATOM	37	HG	SER	4	27.480	35.720	41.710	1.00	0.00
ATOM	38	C	SER	4	29.360	32.730	39.000	1.00	0.00
ATOM	39	O	SER	4	30.420	32.490	39.570	1.00	0.00
ATOM	40	N	PHE	5	29.170	32.570	37.690	1.00	0.00
ATOM	41	H	PHE	5	28.240	32.560	37.320	1.00	0.00
ATOM	42	CA	PHE	5	30.220	32.180	36.730	1.00	0.00
ATOM	43	CB	PHE	5	29.550	31.800	35.410	1.00	0.00
ATOM	44	CG	PHE	5	30.440	31.140	34.350	1.00	0.00
ATOM	45	CD1	PHE	5	31.330	30.100	34.660	1.00	0.00
ATOM	46	HD1	PHE	5	31.520	29.820	35.690	1.00	0.00
ATOM	47	CD2	PHE	5	30.260	31.500	33.020	1.00	0.00

ATOM	48	HD2	PHE	5	29.530	32.270	32.770	1.00	0.00
ATOM	49	CE1	PHE	5	31.990	29.430	33.640	1.00	0.00
ATOM	50	HE1	PHE	5	32.680	28.620	33.890	1.00	0.00
ATOM	51	CE2	PHE	5	30.930	30.830	32.000	1.00	0.00
ATOM	52	HE2	PHE	5	30.770	31.120	30.970	1.00	0.00
ATOM	53	CZ	PHE	5	31.800	29.790	32.310	1.00	0.00
ATOM	54	HZ	PHE	5	32.310	29.250	31.520	1.00	0.00
ATOM	55	C	PHE	5	31.350	33.220	36.560	1.00	0.00
ATOM	56	O	PHE	5	31.380	34.000	35.610	1.00	0.00
ATOM	57	N	GLU	6	32.390	33.000	37.350	1.00	0.00
ATOM	58	H	GLU	6	32.430	32.170	37.920	1.00	0.00
ATOM	59	CA	GLU	6	33.610	33.840	37.490	1.00	0.00
ATOM	60	CB	GLU	6	34.610	33.130	38.430	1.00	0.00
ATOM	61	CG	GLU	6	34.010	32.500	39.700	1.00	0.00
ATOM	62	CD	GLU	6	33.550	31.040	39.560	1.00	0.00
ATOM	63	OE1	GLU	6	33.170	30.600	38.450	1.00	0.00
ATOM	64	OE2	GLU	6	33.530	30.360	40.600	1.00	0.00
ATOM	65	C	GLU	6	34.340	34.050	36.150	1.00	0.00
ATOM	66	O	GLU	6	34.620	35.180	35.750	1.00	0.00
ATOM	67	N	CYS	7	34.400	32.960	35.390	1.00	0.00
ATOM	68	H	CYS	7	34.170	32.070	35.790	1.00	0.00
ATOM	69	CA	CYS	7	35.020	32.890	34.050	1.00	0.00
ATOM	70	CB	CYS	7	34.850	31.500	33.430	1.00	0.00
ATOM	71	SG	CYS	7	35.480	30.150	34.500	1.00	0.00
ATOM	72	HG	CYS	7	35.390	29.190	33.580	1.00	0.00
ATOM	73	C	CYS	7	34.570	33.930	33.010	1.00	0.00
ATOM	74	O	CYS	7	35.350	34.210	32.110	1.00	0.00
ATOM	75	N	ILE	8	33.400	34.550	33.180	1.00	0.00
ATOM	76	H	ILE	8	32.790	34.320	33.950	1.00	0.00
ATOM	77	CA	ILE	8	32.920	35.660	32.310	1.00	0.00
ATOM	78	CB	ILE	8	31.570	36.210	32.800	1.00	0.00
ATOM	79	CG1	ILE	8	30.490	35.120	32.670	1.00	0.00
ATOM	80	CG2	ILE	8	31.130	37.450	32.010	1.00	0.00
ATOM	81	CD	ILE	8	29.190	35.480	33.380	1.00	0.00
ATOM	82	C	ILE	8	33.980	36.780	32.150	1.00	0.00
ATOM	83	O	ILE	8	34.310	37.110	31.010	1.00	0.00
ATOM	84	N	GLN	9	34.660	37.130	33.240	1.00	0.00
ATOM	85	H	GLN	9	34.480	36.710	34.130	1.00	0.00
ATOM	86	CA	GLN	9	35.760	38.130	33.230	1.00	0.00
ATOM	87	CB	GLN	9	36.290	38.330	34.650	1.00	0.00
ATOM	88	CG	GLN	9	35.250	38.950	35.590	1.00	0.00
ATOM	89	CD	GLN	9	35.790	39.040	37.030	1.00	0.00
ATOM	90	OE1	GLN	9	36.480	38.170	37.540	1.00	0.00

ATOM	91	NE2	GLN	9	35.450	40.080	37.730	1.00	0.00
ATOM	92	HE21	GLN	9	34.870	40.810	37.370	1.00	0.00
ATOM	93	HE22	GLN	9	35.820	40.100	38.660	1.00	0.00
ATOM	94	C	GLN	9	36.920	37.730	32.300	1.00	0.00
ATOM	95	O	GLN	9	37.180	38.410	31.300	1.00	0.00
ATOM	96	N	ALA	10	37.430	36.520	32.510	1.00	0.00
ATOM	97	H	ALA	10	37.160	35.990	33.310	1.00	0.00
ATOM	98	CA	ALA	10	38.490	35.900	31.690	1.00	0.00
ATOM	99	CB	ALA	10	38.870	34.540	32.280	1.00	0.00
ATOM	100	C	ALA	10	38.100	35.730	30.210	1.00	0.00
ATOM	101	O	ALA	10	38.800	36.220	29.330	1.00	0.00
ATOM	102	N	ILE	11	36.900	35.200	29.950	1.00	0.00
ATOM	103	H	ILE	11	36.310	34.860	30.700	1.00	0.00
ATOM	104	CA	ILE	11	36.350	35.040	28.580	1.00	0.00
ATOM	105	CB	ILE	11	35.040	34.230	28.590	1.00	0.00
ATOM	106	CG1	ILE	11	35.290	32.830	29.150	1.00	0.00
ATOM	107	CG2	ILE	11	34.420	34.120	27.190	1.00	0.00
ATOM	108	CD	ILE	11	33.990	32.090	29.480	1.00	0.00
ATOM	109	C	ILE	11	36.190	36.400	27.860	1.00	0.00
ATOM	110	O	ILE	11	36.660	36.540	26.730	1.00	0.00
ATOM	111	N	ALA	12	35.660	37.410	28.550	1.00	0.00
ATOM	112	H	ALA	12	35.280	37.270	29.480	1.00	0.00
ATOM	113	CA	ALA	12	35.510	38.780	28.020	1.00	0.00
ATOM	114	CB	ALA	12	34.780	39.660	29.030	1.00	0.00
ATOM	115	C	ALA	12	36.850	39.440	27.640	1.00	0.00
ATOM	116	O	ALA	12	36.940	40.060	26.580	1.00	0.00
ATOM	117	N	ALA	13	37.870	39.260	28.490	1.00	0.00
ATOM	118	H	ALA	13	37.710	38.890	29.410	1.00	0.00
ATOM	119	CA	ALA	13	39.250	39.710	28.230	1.00	0.00
ATOM	120	CB	ALA	13	40.070	39.580	29.510	1.00	0.00
ATOM	121	C	ALA	13	39.950	38.970	27.070	1.00	0.00
ATOM	122	O	ALA	13	40.420	39.590	26.120	1.00	0.00
ATOM	123	N	ASN	14	39.940	37.630	27.130	1.00	0.00
ATOM	124	H	ASN	14	39.640	37.160	27.970	1.00	0.00
ATOM	125	CA	ASN	14	40.570	36.750	26.130	1.00	0.00
ATOM	126	CB	ASN	14	40.600	35.300	26.620	1.00	0.00
ATOM	127	CG	ASN	14	41.420	35.080	27.900	1.00	0.00
ATOM	128	OD1	ASN	14	42.110	35.930	28.440	1.00	0.00
ATOM	129	ND2	ASN	14	41.310	33.910	28.480	1.00	0.00
ATOM	130	HD21	ASN	14	40.730	33.200	28.110	1.00	0.00
ATOM	131	HD22	ASN	14	41.860	33.800	29.300	1.00	0.00
ATOM	132	C	ASN	14	39.970	36.790	24.700	1.00	0.00
ATOM	133	O	ASN	14	40.620	36.350	23.750	1.00	0.00

```
ATOM   134  N   LYS   15     38.680  37.100  24.610  1.00  0.00
ATOM   135  H   LYS   15     38.110  37.190  25.430  1.00  0.00
ATOM   136  CA  LYS   15     37.980  37.280  23.320  1.00  0.00
ATOM   137  CB  LYS   15     36.480  37.140  23.600  1.00  0.00
ATOM   138  CG  LYS   15     35.620  37.060  22.330  1.00  0.00
ATOM   139  CD  LYS   15     34.170  37.350  22.710  1.00  0.00
ATOM   140  CE  LYS   15     33.320  37.530  21.460  1.00  0.00
ATOM   141  NZ  LYS   15     31.990  38.030  21.840  1.00  0.00
ATOM   142  HZ1 LYS   15     31.430  38.140  21.030  1.00  0.00
ATOM   143  HZ2 LYS   15     31.560  37.380  22.470  1.00  0.00
ATOM   144  HZ3 LYS   15     32.100  38.910  22.300  1.00  0.00
ATOM   145  C   LYS   15     38.340  38.650  22.690  1.00  0.00
ATOM   146  OT  LYS   15     37.520  39.560  22.580  1.00  0.00
ATOM   147  O   LYS   15     39.560  38.840  22.160  1.00  0.00
ATOM   148  HO  LYS   15     39.560  39.810  21.940  1.00  0.00
TER
ENDMDL
```

Model 5 (KTSSFECIQAIAANK *_3DStructure)*

```
CRYST1   62.972   62.972   62.972  90.00  90.00  90.00 P 1           1
MODEL        1
ATOM      1  N   LYS    1     33.200  24.570  32.660  1.00  0.00
ATOM      2  H1  LYS    1     33.600  25.160  31.960  1.00  0.00
ATOM      3  H2  LYS    1     32.710  25.190  33.310  1.00  0.00
ATOM      4  CA  LYS    1     32.270  23.600  32.040  1.00  0.00
ATOM      5  CB  LYS    1     32.930  22.900  30.850  1.00  0.00
ATOM      6  CG  LYS    1     32.440  23.500  29.530  1.00  0.00
ATOM      7  CD  LYS    1     30.970  23.140  29.300  1.00  0.00
ATOM      8  CE  LYS    1     30.380  23.850  28.070  1.00  0.00
ATOM      9  NZ  LYS    1     28.980  23.440  27.890  1.00  0.00
ATOM     10  HZ1 LYS    1     28.560  23.930  27.130  1.00  0.00
ATOM     11  HZ2 LYS    1     28.890  22.460  27.780  1.00  0.00
ATOM     12  HZ3 LYS    1     28.430  23.720  28.700  1.00  0.00
ATOM     13  C   LYS    1     31.560  22.570  32.950  1.00  0.00
ATOM     14  O   LYS    1     30.510  22.040  32.610  1.00  0.00
ATOM     15  N   THR    2     32.260  22.210  34.020  1.00  0.00
ATOM     16  H   THR    2     33.250  22.350  34.040  1.00  0.00
ATOM     17  CA  THR    2     31.660  21.590  35.230  1.00  0.00
ATOM     18  CB  THR    2     32.750  21.040  36.170  1.00  0.00
ATOM     19  OG1 THR    2     33.750  22.050  36.370  1.00  0.00
ATOM     20  HG1 THR    2     34.400  21.710  37.060  1.00  0.00
ATOM     21  CG2 THR    2     33.350  19.750  35.620  1.00  0.00
ATOM     22  C   THR    2     30.820  22.610  36.020  1.00  0.00
```

ATOM	23	O	THR	2	29.960	22.260	36.830	1.00	0.00
ATOM	24	N	SER	3	31.280	23.850	35.940	1.00	0.00
ATOM	25	H	SER	3	32.210	23.980	35.600	1.00	0.00
ATOM	26	CA	SER	3	30.590	25.100	36.360	1.00	0.00
ATOM	27	CB	SER	3	31.500	26.280	36.020	1.00	0.00
ATOM	28	OG	SER	3	31.810	26.260	34.610	1.00	0.00
ATOM	29	HG	SER	3	30.910	26.210	34.130	1.00	0.00
ATOM	30	C	SER	3	29.260	25.310	35.610	1.00	0.00
ATOM	31	O	SER	3	29.270	25.440	34.390	1.00	0.00
ATOM	32	N	SER	4	28.260	25.730	36.380	1.00	0.00
ATOM	33	H	SER	4	28.370	25.820	37.370	1.00	0.00
ATOM	34	CA	SER	4	26.890	26.060	35.900	1.00	0.00
ATOM	35	CB	SER	4	26.030	26.550	37.060	1.00	0.00
ATOM	36	OG	SER	4	26.730	27.600	37.750	1.00	0.00
ATOM	37	HG	SER	4	26.080	28.080	38.340	1.00	0.00
ATOM	38	C	SER	4	26.820	27.120	34.790	1.00	0.00
ATOM	39	O	SER	4	25.920	27.120	33.960	1.00	0.00
ATOM	40	N	PHE	5	27.670	28.140	34.910	1.00	0.00
ATOM	41	H	PHE	5	28.040	28.350	35.810	1.00	0.00
ATOM	42	CA	PHE	5	27.860	29.190	33.890	1.00	0.00
ATOM	43	CB	PHE	5	28.770	30.300	34.440	1.00	0.00
ATOM	44	CG	PHE	5	28.150	31.050	35.620	1.00	0.00
ATOM	45	CD1	PHE	5	28.480	30.700	36.920	1.00	0.00
ATOM	46	HD1	PHE	5	29.250	29.960	37.110	1.00	0.00
ATOM	47	CD2	PHE	5	27.230	32.060	35.380	1.00	0.00
ATOM	48	HD2	PHE	5	26.990	32.360	34.360	1.00	0.00
ATOM	49	CE1	PHE	5	27.870	31.350	37.990	1.00	0.00
ATOM	50	HE1	PHE	5	28.140	31.080	39.010	1.00	0.00
ATOM	51	CE2	PHE	5	26.620	32.720	36.450	1.00	0.00
ATOM	52	HE2	PHE	5	25.900	33.510	36.260	1.00	0.00
ATOM	53	CZ	PHE	5	26.940	32.360	37.750	1.00	0.00
ATOM	54	HZ	PHE	5	26.470	32.880	38.590	1.00	0.00
ATOM	55	C	PHE	5	28.370	28.680	32.530	1.00	0.00
ATOM	56	O	PHE	5	29.560	28.720	32.220	1.00	0.00
ATOM	57	N	GLU	6	27.410	28.210	31.730	1.00	0.00
ATOM	58	H	GLU	6	26.580	27.830	32.160	1.00	0.00
ATOM	59	CA	GLU	6	27.590	27.800	30.320	1.00	0.00
ATOM	60	CB	GLU	6	26.210	27.490	29.730	1.00	0.00
ATOM	61	CG	GLU	6	25.720	26.080	30.050	1.00	0.00
ATOM	62	CD	GLU	6	26.460	25.030	29.210	1.00	0.00
ATOM	63	OE1	GLU	6	26.640	25.250	27.990	1.00	0.00
ATOM	64	OE2	GLU	6	26.850	24.000	29.800	1.00	0.00
ATOM	65	C	GLU	6	28.290	28.820	29.390	1.00	0.00

ATOM	66	O	GLU	6	29.220	28.480	28.650	1.00	0.00
ATOM	67	N	CYS	7	27.870	30.080	29.490	1.00	0.00
ATOM	68	H	CYS	7	27.190	30.330	30.170	1.00	0.00
ATOM	69	CA	CYS	7	28.360	31.170	28.610	1.00	0.00
ATOM	70	CB	CYS	7	27.410	32.370	28.600	1.00	0.00
ATOM	71	SG	CYS	7	27.890	33.640	27.380	1.00	0.00
ATOM	72	HG	CYS	7	26.750	34.330	27.440	1.00	0.00
ATOM	73	C	CYS	7	29.810	31.610	28.880	1.00	0.00
ATOM	74	O	CYS	7	30.100	32.620	29.510	1.00	0.00
ATOM	75	N	ILE	8	30.710	30.860	28.240	1.00	0.00
ATOM	76	H	ILE	8	30.440	29.920	28.000	1.00	0.00
ATOM	77	CA	ILE	8	32.160	31.130	28.190	1.00	0.00
ATOM	78	CB	ILE	8	32.880	30.030	27.390	1.00	0.00
ATOM	79	CG1	ILE	8	32.590	28.620	27.930	1.00	0.00
ATOM	80	CG2	ILE	8	34.400	30.260	27.300	1.00	0.00
ATOM	81	CD	ILE	8	32.990	28.410	29.400	1.00	0.00
ATOM	82	C	ILE	8	32.490	32.550	27.660	1.00	0.00
ATOM	83	O	ILE	8	33.320	33.240	28.250	1.00	0.00
ATOM	84	N	GLN	9	31.700	33.020	26.700	1.00	0.00
ATOM	85	H	GLN	9	30.990	32.440	26.290	1.00	0.00
ATOM	86	CA	GLN	9	31.810	34.380	26.120	1.00	0.00
ATOM	87	CB	GLN	9	30.770	34.550	25.000	1.00	0.00
ATOM	88	CG	GLN	9	31.040	33.580	23.850	1.00	0.00
ATOM	89	CD	GLN	9	29.850	33.550	22.880	1.00	0.00
ATOM	90	OE1	GLN	9	28.830	32.940	23.130	1.00	0.00
ATOM	91	NE2	GLN	9	29.950	34.230	21.770	1.00	0.00
ATOM	92	HE21	GLN	9	30.760	34.760	21.540	1.00	0.00
ATOM	93	HE22	GLN	9	29.150	34.190	21.170	1.00	0.00
ATOM	94	C	GLN	9	31.650	35.500	27.160	1.00	0.00
ATOM	95	O	GLN	9	32.540	36.340	27.290	1.00	0.00
ATOM	96	N	ALA	10	30.620	35.390	28.010	1.00	0.00
ATOM	97	H	ALA	10	29.890	34.720	27.860	1.00	0.00
ATOM	98	CA	ALA	10	30.400	36.300	29.150	1.00	0.00
ATOM	99	CB	ALA	10	29.070	35.980	29.840	1.00	0.00
ATOM	100	C	ALA	10	31.540	36.260	30.180	1.00	0.00
ATOM	101	O	ALA	10	32.220	37.260	30.360	1.00	0.00
ATOM	102	N	ILE	11	31.920	35.050	30.600	1.00	0.00
ATOM	103	H	ILE	11	31.420	34.230	30.330	1.00	0.00
ATOM	104	CA	ILE	11	33.040	34.820	31.560	1.00	0.00
ATOM	105	CB	ILE	11	33.180	33.320	31.880	1.00	0.00
ATOM	106	CG1	ILE	11	31.850	32.740	32.390	1.00	0.00
ATOM	107	CG2	ILE	11	34.280	33.060	32.910	1.00	0.00
ATOM	108	CD	ILE	11	31.860	31.210	32.400	1.00	0.00

```
ATOM   109  C   ILE    11      34.380  35.400  31.040  1.00  0.00
ATOM   110  O   ILE    11      35.170  35.930  31.830  1.00  0.00
ATOM   111  N   ALA    12      34.630  35.280  29.740  1.00  0.00
ATOM   112  H   ALA    12      34.090  34.650  29.170  1.00  0.00
ATOM   113  CA  ALA    12      35.760  35.940  29.050  1.00  0.00
ATOM   114  CB  ALA    12      35.880  35.410  27.620  1.00  0.00
ATOM   115  C   ALA    12      35.660  37.480  29.050  1.00  0.00
ATOM   116  O   ALA    12      36.490  38.140  29.670  1.00  0.00
ATOM   117  N   ALA    13      34.560  38.010  28.510  1.00  0.00
ATOM   118  H   ALA    13      33.820  37.430  28.150  1.00  0.00
ATOM   119  CA  ALA    13      34.310  39.470  28.390  1.00  0.00
ATOM   120  CB  ALA    13      33.000  39.700  27.640  1.00  0.00
ATOM   121  C   ALA    13      34.280  40.240  29.720  1.00  0.00
ATOM   122  O   ALA    13      34.920  41.280  29.850  1.00  0.00
ATOM   123  N   ASN    14      33.620  39.660  30.720  1.00  0.00
ATOM   124  H   ASN    14      33.000  38.880  30.550  1.00  0.00
ATOM   125  CA  ASN    14      33.550  40.180  32.110  1.00  0.00
ATOM   126  CB  ASN    14      32.500  39.400  32.910  1.00  0.00
ATOM   127  CG  ASN    14      31.070  39.710  32.470  1.00  0.00
ATOM   128  OD1 ASN    14      30.630  39.490  31.350  1.00  0.00
ATOM   129  ND2 ASN    14      30.270  40.220  33.380  1.00  0.00
ATOM   130 HD21 ASN    14      30.590  40.390  34.310  1.00  0.00
ATOM   131 HD22 ASN    14      29.350  40.430  33.070  1.00  0.00
ATOM   132  C   ASN    14      34.880  40.210  32.900  1.00  0.00
ATOM   133  O   ASN    14      34.930  40.810  33.980  1.00  0.00
ATOM   134  N   LYS    15      35.900  39.510  32.410  1.00  0.00
ATOM   135  H   LYS    15      35.840  39.060  31.520  1.00  0.00
ATOM   136  CA  LYS    15      37.210  39.420  33.070  1.00  0.00
ATOM   137  CB  LYS    15      37.700  37.980  32.900  1.00  0.00
ATOM   138  CG  LYS    15      38.950  37.660  33.720  1.00  0.00
ATOM   139  CD  LYS    15      39.620  36.420  33.140  1.00  0.00
ATOM   140  CE  LYS    15      41.000  36.210  33.760  1.00  0.00
ATOM   141  NZ  LYS    15      41.720  35.170  33.020  1.00  0.00
ATOM   142  HZ1 LYS    15      42.620  35.020  33.430  1.00  0.00
ATOM   143  HZ2 LYS    15      41.190  34.320  33.040  1.00  0.00
ATOM   144  HZ3 LYS    15      41.820  35.460  32.070  1.00  0.00
ATOM   145  C   LYS    15      38.180  40.450  32.460  1.00  0.00
ATOM   146  OT  LYS    15      39.130  40.130  31.760  1.00  0.00
ATOM   147  O   LYS    15      38.030  41.760  32.750  1.00  0.00
ATOM   148  HO  LYS    15      38.700  42.190  32.160  1.00  0.00
TER
ENDMDL
```

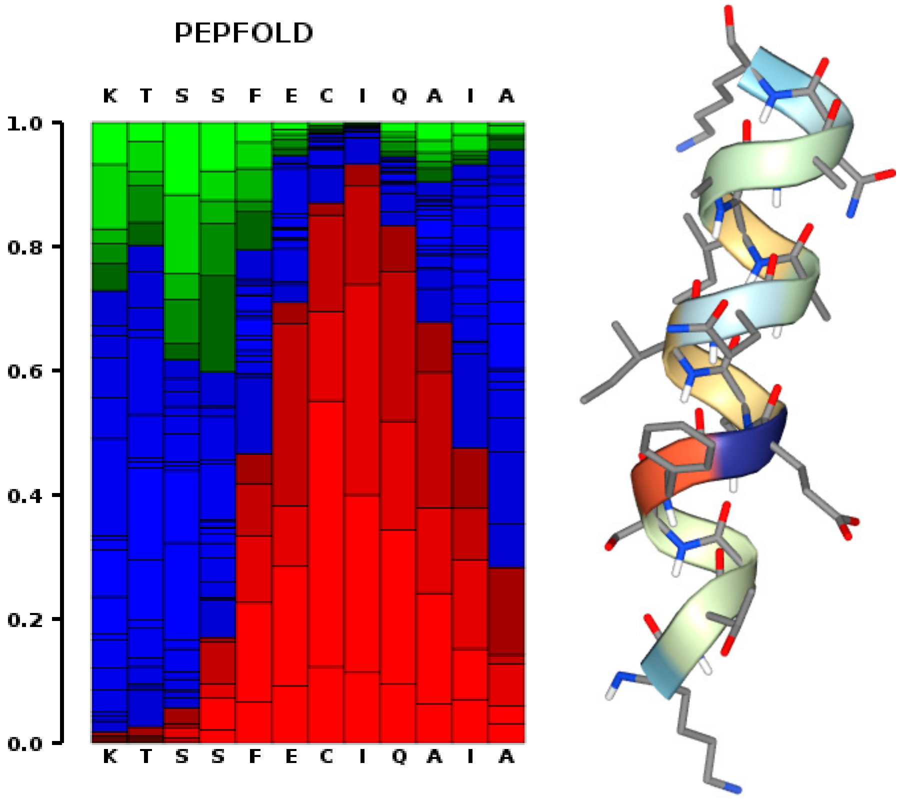

Figure S3 3D structure of peptide and Predicted Local Structure Profile KTSSFECIQAIAANK job execution pep-fold4.py --maxSimSze 50 --maxSeqSze 50 --noMQA --sortKey sOPEP -s iSeq.fasta -l PEPFOLD --generator fbt --nRuns 100 --mcSteps 30000 --mcT 370 --seed 1 --user_pKa pKa.data
Solvent pH 7.5, Ionic strength (150mM)

Table S4 KTSSFECIQAIAANK PEPFOLD-5 bestmodels.pdb

MODEL 1

ATOM	0	N	LYS	A	1	-8.167	1.833	6.282
ATOM	1	CA	LYS	A	1	-9.190	1.093	7.078
ATOM	2	C	LYS	A	1	-10.415	1.981	7.307
ATOM	3	O	LYS	A	1	-10.441	3.135	6.927
ATOM	4	H	LYS	A	1	-7.285	1.382	6.040
ATOM	5	CB	LYS	A	1	-8.590	0.692	8.438
ATOM	6	CG	LYS	A	1	-7.529	-0.387	8.296
ATOM	7	CD	LYS	A	1	-6.953	-0.800	9.641
ATOM	8	CE	LYS	A	1	-5.892	-1.879	9.500
ATOM	9	NZ	LYS	A	1	-5.332	-2.281	10.809
ATOM	0	N	THR	A	2	-11.432	1.448	7.930
ATOM	1	CA	THR	A	2	-12.657	2.256	8.188
ATOM	2	C	THR	A	2	-12.329	3.367	9.187
ATOM	3	O	THR	A	2	-12.981	4.391	9.228
ATOM	4	H	THR	A	2	-11.391	0.518	8.227

ATOM 5 CB THR A 2 -13.758 1.352 8.771
ATOM 6 OG1 THR A 2 -14.092 0.323 7.836
ATOM 7 CG2 THR A 2 -15.026 2.129 9.084
ATOM 0 N SER A 3 -11.318 3.176 9.989
ATOM 1 CA SER A 3 -10.947 4.224 10.979
ATOM 2 C SER A 3 -10.854 5.571 10.266
ATOM 3 O SER A 3 -11.290 6.587 10.770
ATOM 4 H SER A 3 -10.804 2.346 9.941
ATOM 5 CB SER A 3 -9.585 3.881 11.611
ATOM 6 OG SER A 3 -8.594 3.935 10.581
ATOM 0 N SER A 4 -10.296 5.586 9.090
ATOM 1 CA SER A 4 -10.190 6.871 8.349
ATOM 2 C SER A 4 -11.472 7.641 8.095
ATOM 3 O SER A 4 -11.505 8.865 8.007
ATOM 4 H SER A 4 -9.949 4.761 8.697
ATOM 5 CB SER A 4 -9.559 6.564 6.978
ATOM 6 OG SER A 4 -10.563 5.964 6.154
ATOM 0 N PHE A 5 -12.549 6.870 7.963
ATOM 1 CA PHE A 5 -13.877 7.432 7.745
ATOM 2 C PHE A 5 -14.606 7.975 8.950
ATOM 3 O PHE A 5 -15.180 9.065 8.966
ATOM 4 H PHE A 5 -12.447 5.899 8.015
ATOM 5 CB PHE A 5 -14.756 6.323 7.137
ATOM 6 CG PHE A 5 -16.188 6.784 6.924
ATOM 7 CD1 PHE A 5 -16.498 7.587 5.858
ATOM 8 CE1 PHE A 5 -17.789 8.003 5.666
ATOM 9 CZ PHE A 5 -18.771 7.615 6.539
ATOM 10 CE2 PHE A 5 -18.461 6.811 7.604
ATOM 11 CD2 PHE A 5 -17.170 6.396 7.797
ATOM 0 N GLU A 6 -14.555 7.171 10.006
ATOM 1 CA GLU A 6 -15.251 7.507 11.241
ATOM 2 C GLU A 6 -14.426 8.608 11.728
ATOM 3 O GLU A 6 -15.070 9.502 12.256
ATOM 4 H GLU A 6 -14.043 6.340 9.955
ATOM 5 CB GLU A 6 -15.258 6.326 12.229
ATOM 6 CG GLU A 6 -16.154 5.194 11.756
ATOM 7 CD GLU A 6 -16.124 4.008 12.707
ATOM 8 OE1 GLU A 6 -16.885 3.046 12.523
ATOM 9 OE2 GLU A 6 -15.337 4.002 13.665
ATOM 0 N CYS A 7 -13.117 8.702 11.576
ATOM 1 CA CYS A 7 -12.422 9.913 12.048
ATOM 2 C CYS A 7 -13.019 11.140 11.320
ATOM 3 O CYS A 7 -13.298 12.120 12.018

```
ATOM    4  H   CYS A   7     -12.615  7.977  11.154
ATOM    5  CB  CYS A   7     -10.918  9.806  11.734
ATOM    6  SG  CYS A   7      -9.960  8.784  12.896
ATOM    0  N   ILE A   8     -13.216 11.099  10.005
ATOM    1  CA  ILE A   8     -13.746 12.229   9.246
ATOM    2  C   ILE A   8     -15.053 12.650   9.852
ATOM    3  O   ILE A   8     -15.314 13.796  10.210
ATOM    4  H   ILE A   8     -12.997 10.276   9.525
ATOM    5  CB  ILE A   8     -13.968 11.811   7.780
ATOM    6  CG1 ILE A   8     -12.669 11.417   7.097
ATOM    7  CD  ILE A   8     -12.959 10.840   5.721
ATOM    8  CG2 ILE A   8     -14.586 12.960   7.000
ATOM    0  N   GLN A   9     -15.898 11.666  10.055
ATOM    1  CA  GLN A   9     -17.190 11.988  10.607
ATOM    2  C   GLN A   9     -17.105 12.606  11.974
ATOM    3  O   GLN A   9     -17.787 13.568  12.290
ATOM    4  H   GLN A   9     -15.660 10.744   9.839
ATOM    5  CB  GLN A   9     -18.020 10.695  10.704
ATOM    6  CG  GLN A   9     -18.273 10.076   9.339
ATOM    7  CD  GLN A   9     -19.085 10.994   8.440
ATOM    8  OE1 GLN A   9     -20.159 11.464   8.842
ATOM    9  NE2 GLN A   9     -18.639 11.294   7.212
ATOM    0  N   ALA A  10     -16.201 12.103  12.796
ATOM    1  CA  ALA A  10     -16.011 12.722  14.121
ATOM    2  C   ALA A  10     -15.474 14.157  14.074
ATOM    3  O   ALA A  10     -15.946 15.023  14.815
ATOM    4  H   ALA A  10     -15.665 11.331  12.530
ATOM    5  CB  ALA A  10     -15.013 11.861  14.919
ATOM    0  N   ILE A  11     -14.498 14.416  13.205
ATOM    1  CA  ILE A  11     -13.931 15.758  13.122
ATOM    2  C   ILE A  11     -15.070 16.666  12.669
ATOM    3  O   ILE A  11     -15.307 17.724  13.247
ATOM    4  H   ILE A  11     -14.161 13.708  12.622
ATOM    5  CB  ILE A  11     -12.780 15.793  12.099
ATOM    6  CG1 ILE A  11     -11.618 14.909  12.519
ATOM    7  CD  ILE A  11     -10.713 14.639  11.328
ATOM    8  CG2 ILE A  11     -12.264 17.215  11.945
ATOM    0  N   ALA A  12     -15.833 16.243  11.688
ATOM    1  CA  ALA A  12     -16.890 17.109  11.231
ATOM    2  C   ALA A  12     -17.903 17.410  12.300
ATOM    3  O   ALA A  12     -18.345 18.535  12.470
ATOM    4  H   ALA A  12     -15.689 15.367  11.279
ATOM    5  CB  ALA A  12     -17.609 16.430  10.051
```

```
ATOM      0  N   ALA A  13     -18.238  16.411  13.096
ATOM      1  CA  ALA A  13     -19.163  16.663  14.217
ATOM      2  C   ALA A  13     -18.559  17.608  15.225
ATOM      3  O   ALA A  13     -19.242  18.495  15.713
ATOM      4  H   ALA A  13     -17.878  15.515  12.946
ATOM      5  CB  ALA A  13     -19.484  15.327  14.914
ATOM      0  N   ASN A  14     -17.277  17.488  15.517
ATOM      1  CA  ASN A  14     -16.657  18.448  16.448
ATOM      2  C   ASN A  14     -16.480  19.903  16.071
ATOM      3  O   ASN A  14     -16.747  20.833  16.833
ATOM      4  H   ASN A  14     -16.744  16.771  15.121
ATOM      5  CB  ASN A  14     -15.251  17.907  16.765
ATOM      6  CG  ASN A  14     -15.305  16.618  17.569
ATOM      7  OD1 ASN A  14     -16.170  16.469  18.444
ATOM      8  ND2 ASN A  14     -14.363  15.726  17.232
ATOM      0  N   LYS A  15     -16.028  20.090  14.836
ATOM      1  CA  LYS A  15     -15.749  21.428  14.332
ATOM      2  C   LYS A  15     -16.808  22.074  13.451
ATOM      3  O   LYS A  15     -17.067  23.271  13.578
ATOM      4  H   LYS A  15     -15.880  19.318  14.255
ATOM      5  CB  LYS A  15     -14.448  21.353  13.511
ATOM      6  CG  LYS A  15     -13.243  21.036  14.381
ATOM      7  CD  LYS A  15     -11.955  20.987  13.575
ATOM      8  CE  LYS A  15     -10.750  20.670  14.445
ATOM      9  NZ  LYS A  15      -9.496  20.622  13.660
TER
ENDMDL
```

MODEL 2 KTSSFECIQAIAANK

```
ATOM      0  N   LYS A   1     -15.829   0.820   8.146
ATOM      1  CA  LYS A   1     -15.228   2.150   8.143
ATOM      2  C   LYS A   1     -13.852   2.008   8.779
ATOM      3  O   LYS A   1     -13.723   1.426   9.851
ATOM      4  H   LYS A   1     -16.764   0.682   7.762
ATOM      5  CB  LYS A   1     -16.091   3.127   8.962
ATOM      6  CG  LYS A   1     -17.393   3.468   8.254
ATOM      7  CD  LYS A   1     -18.234   4.451   9.051
ATOM      8  CE  LYS A   1     -19.535   4.792   8.344
ATOM      9  NZ  LYS A   1     -20.354   5.749   9.120
ATOM      0  N   THR A   2     -12.833   2.502   8.086
ATOM      1  CA  THR A   2     -11.483   2.538   8.625
ATOM      2  C   THR A   2     -11.240   3.840   9.377
ATOM      3  O   THR A   2     -11.961   4.820   9.174
ATOM      4  H   THR A   2     -12.993   2.852   7.188
```

ATOM	5	CB	THR	A	2	-10.471	2.425	7.470
ATOM	6	OG1	THR	A	2	-10.658	1.190	6.774
ATOM	7	CG2	THR	A	2	-9.034	2.473	7.964
ATOM	0	N	SER	A	3	-10.210	3.845	10.234
ATOM	1	CA	SER	A	3	-9.952	4.974	11.128
ATOM	2	C	SER	A	3	-9.717	6.278	10.370
ATOM	3	O	SER	A	3	-9.915	7.369	10.912
ATOM	4	H	SER	A	3	-9.612	3.072	10.263
ATOM	5	CB	SER	A	3	-8.702	4.663	11.972
ATOM	6	OG	SER	A	3	-7.576	4.616	11.093
ATOM	0	N	SER	A	4	-9.338	6.169	9.101
ATOM	1	CA	SER	A	4	-9.098	7.361	8.305
ATOM	2	C	SER	A	4	-10.426	7.977	7.869
ATOM	3	O	SER	A	4	-10.491	9.179	7.624
ATOM	4	H	SER	A	4	-9.221	5.285	8.700
ATOM	5	CB	SER	A	4	-8.279	6.985	7.056
ATOM	6	OG	SER	A	4	-9.082	6.126	6.243
ATOM	0	N	PHE	A	5	-11.471	7.157	7.787
ATOM	1	CA	PHE	A	5	-12.803	7.639	7.437
ATOM	2	C	PHE	A	5	-13.627	7.999	8.659
ATOM	3	O	PHE	A	5	-14.356	8.990	8.672
ATOM	4	H	PHE	A	5	-11.343	6.205	7.966
ATOM	5	CB	PHE	A	5	-13.539	6.536	6.653
ATOM	6	CG	PHE	A	5	-12.915	6.295	5.288
ATOM	7	CD1	PHE	A	5	-11.958	5.327	5.134
ATOM	8	CE1	PHE	A	5	-11.396	5.110	3.903
ATOM	9	CZ	PHE	A	5	-11.791	5.860	2.827
ATOM	10	CE2	PHE	A	5	-12.748	6.828	2.982
ATOM	11	CD2	PHE	A	5	-13.310	7.045	4.212
ATOM	0	N	GLU	A	6	-13.525	7.167	9.698
ATOM	1	CA	GLU	A	6	-14.291	7.402	10.920
ATOM	2	C	GLU	A	6	-13.595	8.554	11.489
ATOM	3	O	GLU	A	6	-14.335	9.367	12.020
ATOM	4	H	GLU	A	6	-12.931	6.392	9.641
ATOM	5	CB	GLU	A	6	-14.230	6.187	11.865
ATOM	6	CG	GLU	A	6	-14.955	4.981	11.291
ATOM	7	CD	GLU	A	6	-14.842	3.765	12.196
ATOM	8	OE1	GLU	A	6	-14.031	3.763	13.134
ATOM	9	OE2	GLU	A	6	-15.561	2.775	11.996
ATOM	0	N	CYS	A	7	-12.293	8.763	11.403
ATOM	1	CA	CYS	A	7	-11.725	10.006	11.955
ATOM	2	C	CYS	A	7	-12.392	11.210	11.249
ATOM	3	O	CYS	A	7	-12.784	12.131	11.971

ATOM	4	H	CYS	A	7	-11.713	8.101	10.977
ATOM	5	CB	CYS	A	7	-10.205	10.038	11.709
ATOM	6	SG	CYS	A	7	-9.256	8.758	12.588
ATOM	0	N	ILE	A	8	-12.526	11.209	9.925
ATOM	1	CA	ILE	A	8	-13.116	12.324	9.188
ATOM	2	C	ILE	A	8	-14.480	12.608	9.748
ATOM	3	O	ILE	A	8	-14.853	13.712	10.138
ATOM	4	H	ILE	A	8	-12.217	10.430	9.424
ATOM	5	CB	ILE	A	8	-13.238	11.953	7.698
ATOM	6	CG1	ILE	A	8	-13.675	13.137	6.850
ATOM	7	CD	ILE	A	8	-13.485	12.819	5.376
ATOM	8	CG2	ILE	A	8	-14.257	10.840	7.520
ATOM	0	N	GLN	A	9	-15.247	11.550	9.870
ATOM	1	CA	GLN	A	9	-16.584	11.738	10.373
ATOM	2	C	GLN	A	9	-16.612	12.301	11.766
ATOM	3	O	GLN	A	9	-17.387	13.188	12.087
ATOM	4	H	GLN	A	9	-14.921	10.660	9.629
ATOM	5	CB	GLN	A	9	-17.305	10.378	10.379
ATOM	6	CG	GLN	A	9	-17.552	9.858	8.973
ATOM	7	CD	GLN	A	9	-18.209	8.487	8.980
ATOM	8	OE1	GLN	A	9	-18.349	7.872	10.047
ATOM	9	NE2	GLN	A	9	-18.636	7.943	7.832
ATOM	0	N	ALA	A	10	-15.706	11.840	12.610
ATOM	1	CA	ALA	A	10	-15.627	12.414	13.965
ATOM	2	C	ALA	A	10	-15.327	13.883	13.921
ATOM	3	O	ALA	A	10	-15.969	14.748	14.512
ATOM	4	H	ALA	A	10	-15.095	11.127	12.339
ATOM	5	CB	ALA	A	10	-14.511	11.699	14.750
ATOM	0	N	ILE	A	11	-14.344	14.202	13.111
ATOM	1	CA	ILE	A	11	-13.966	15.589	13.020
ATOM	2	C	ILE	A	11	-15.076	16.466	12.516
ATOM	3	O	ILE	A	11	-15.321	17.550	13.022
ATOM	4	H	ILE	A	11	-13.883	13.519	12.585
ATOM	5	CB	ILE	A	11	-12.769	15.712	12.059
ATOM	6	CG1	ILE	A	11	-11.550	14.962	12.569
ATOM	7	CD	ILE	A	11	-10.461	14.956	11.507
ATOM	8	CG2	ILE	A	11	-12.390	17.173	11.885
ATOM	0	N	ALA	A	12	-15.824	15.976	11.543
ATOM	1	CA	ALA	A	12	-16.982	16.753	11.064
ATOM	2	C	ALA	A	12	-17.975	16.990	12.163
ATOM	3	O	ALA	A	12	-18.439	18.087	12.464
ATOM	4	H	ALA	A	12	-15.614	15.109	11.146
ATOM	5	CB	ALA	A	12	-17.671	15.978	9.926

ATOM 0 N ALA A 13 -18.261 15.917 12.863
ATOM 1 CA ALA A 13 -19.226 16.041 13.927
ATOM 2 C ALA A 13 -18.845 16.993 15.062
ATOM 3 O ALA A 13 -19.649 17.793 15.546
ATOM 4 H ALA A 13 -17.833 15.060 12.670
ATOM 5 CB ALA A 13 -19.442 14.643 14.536
ATOM 0 N ASN A 14 -17.580 16.915 15.473
ATOM 1 CA ASN A 14 -17.108 17.779 16.550
ATOM 2 C ASN A 14 -17.101 19.252 16.040
ATOM 3 O ASN A 14 -17.483 20.115 16.834
ATOM 4 H ASN A 14 -16.967 16.281 15.052
ATOM 5 CB ASN A 14 -15.682 17.367 16.961
ATOM 6 CG ASN A 14 -15.667 16.054 17.727
ATOM 7 OD1 ASN A 14 -16.506 15.846 18.616
ATOM 8 ND2 ASN A 14 -14.699 15.211 17.345
ATOM 0 N LYS A 15 -16.701 19.546 14.804
ATOM 1 CA LYS A 15 -16.680 20.934 14.353
ATOM 2 C LYS A 15 -18.082 21.464 14.062
ATOM 3 O LYS A 15 -18.239 22.628 13.683
ATOM 4 H LYS A 15 -16.422 18.833 14.197
ATOM 5 CB LYS A 15 -15.838 21.029 13.068
ATOM 6 CG LYS A 15 -14.362 20.782 13.334
ATOM 7 CD LYS A 15 -13.527 20.899 12.070
ATOM 8 CE LYS A 15 -12.051 20.653 12.336
ATOM 9 NZ LYS A 15 -11.238 20.767 11.105
TER
ENDMDL
MODEL 3 KTSSFECIQAIAANK
ATOM 0 N LYS A 1 -12.701 -1.012 9.537
ATOM 1 CA LYS A 1 -11.252 -0.924 9.447
ATOM 2 C LYS A 1 -10.830 0.166 8.468
ATOM 3 O LYS A 1 -9.681 0.193 8.025
ATOM 4 H LYS A 1 -13.133 -1.698 10.155
ATOM 5 CB LYS A 1 -10.693 -2.276 8.965
ATOM 6 CG LYS A 1 -10.844 -3.362 10.017
ATOM 7 CD LYS A 1 -10.271 -4.691 9.551
ATOM 8 CE LYS A 1 -10.422 -5.777 10.603
ATOM 9 NZ LYS A 1 -9.864 -7.070 10.149
ATOM 0 N THR A 2 -11.767 1.043 8.114
ATOM 1 CA THR A 2 -11.469 2.222 7.282
ATOM 2 C THR A 2 -11.494 3.444 8.199
ATOM 3 O THR A 2 -12.481 4.187 8.257
ATOM 4 H THR A 2 -12.685 0.901 8.417

ATOM	5	CB	THR	A	2	-12.531	2.372	6.177
ATOM	6	OG1	THR	A	2	-12.506	1.230	5.317
ATOM	7	CG2	THR	A	2	-12.295	3.607	5.324
ATOM	0	N	SER	A	3	-10.376	3.667	8.886
ATOM	1	CA	SER	A	3	-10.295	4.692	9.930
ATOM	2	C	SER	A	3	-10.606	6.096	9.382
ATOM	3	O	SER	A	3	-11.320	6.874	10.000
ATOM	4	H	SER	A	3	-9.583	3.131	8.688
ATOM	5	CB	SER	A	3	-8.872	4.693	10.519
ATOM	6	OG	SER	A	3	-7.971	5.122	9.496
ATOM	0	N	SER	A	4	-10.066	6.404	8.211
ATOM	1	CA	SER	A	4	-10.188	7.730	7.643
ATOM	2	C	SER	A	4	-11.605	8.293	7.717
ATOM	3	O	SER	A	4	-11.763	9.426	8.166
ATOM	4	H	SER	A	4	-9.573	5.719	7.718
ATOM	5	CB	SER	A	4	-9.766	7.670	6.163
ATOM	6	OG	SER	A	4	-10.726	6.876	5.461
ATOM	0	N	PHE	A	5	-12.617	7.537	7.307
ATOM	1	CA	PHE	A	5	-13.978	8.044	7.369
ATOM	2	C	PHE	A	5	-14.555	8.302	8.770
ATOM	3	O	PHE	A	5	-15.210	9.326	8.970
ATOM	4	H	PHE	A	5	-12.448	6.638	6.964
ATOM	5	CB	PHE	A	5	-14.891	7.020	6.671
ATOM	6	CG	PHE	A	5	-16.355	7.427	6.733
ATOM	7	CD1	PHE	A	5	-16.832	8.398	5.893
ATOM	8	CE1	PHE	A	5	-18.151	8.764	5.949
ATOM	9	CZ	PHE	A	5	-18.992	8.160	6.845
ATOM	10	CE2	PHE	A	5	-18.515	7.189	7.686
ATOM	11	CD2	PHE	A	5	-17.196	6.822	7.630
ATOM	0	N	GLU	A	6	-14.313	7.404	9.719
ATOM	1	CA	GLU	A	6	-14.882	7.548	11.052
ATOM	2	C	GLU	A	6	-14.098	8.667	11.564
ATOM	3	O	GLU	A	6	-14.745	9.438	12.255
ATOM	4	H	GLU	A	6	-13.744	6.635	9.520
ATOM	5	CB	GLU	A	6	-14.674	6.278	11.898
ATOM	6	CG	GLU	A	6	-15.525	5.119	11.403
ATOM	7	CD	GLU	A	6	-15.284	3.852	12.208
ATOM	8	OE1	GLU	A	6	-15.981	2.847	12.004
ATOM	9	OE2	GLU	A	6	-14.392	3.824	13.068
ATOM	0	N	CYS	A	7	-12.829	8.898	11.281
ATOM	1	CA	CYS	A	7	-12.183	10.115	11.805
ATOM	2	C	CYS	A	7	-12.761	11.422	11.251
ATOM	3	O	CYS	A	7	-12.966	12.380	12.001

ATOM	4	H	CYS	A	7	-12.322	8.269	10.729
ATOM	5	CB	CYS	A	7	-10.686	10.062	11.449
ATOM	6	SG	CYS	A	7	-9.739	8.761	12.299
ATOM	0	N	ILE	A	8	-13.032	11.466	9.947
ATOM	1	CA	ILE	A	8	-13.568	12.687	9.353
ATOM	2	C	ILE	A	8	-14.975	12.856	9.947
ATOM	3	O	ILE	A	8	-15.305	13.995	10.238
ATOM	4	H	ILE	A	8	-12.872	10.681	9.388
ATOM	5	CB	ILE	A	8	-13.646	12.550	7.821
ATOM	6	CG1	ILE	A	8	-12.272	12.389	7.192
ATOM	7	CD	ILE	A	8	-12.407	11.847	5.779
ATOM	8	CG2	ILE	A	8	-14.301	13.784	7.222
ATOM	0	N	GLN	A	9	-15.770	11.828	10.185
ATOM	1	CA	GLN	A	9	-17.080	12.062	10.819
ATOM	2	C	GLN	A	9	-16.924	12.599	12.220
ATOM	3	O	GLN	A	9	-17.644	13.506	12.608
ATOM	4	H	GLN	A	9	-15.494	10.923	9.943
ATOM	5	CB	GLN	A	9	-17.855	10.733	10.877
ATOM	6	CG	GLN	A	9	-18.353	10.304	9.507
ATOM	7	CD	GLN	A	9	-19.508	11.166	9.025
ATOM	8	OE1	GLN	A	9	-20.574	11.186	9.659
ATOM	9	NE2	GLN	A	9	-19.375	11.905	7.915
ATOM	0	N	ALA	A	10	-15.964	12.109	12.983
ATOM	1	CA	ALA	A	10	-15.755	12.672	14.329
ATOM	2	C	ALA	A	10	-15.298	14.135	14.335
ATOM	3	O	ALA	A	10	-15.786	14.937	15.135
ATOM	4	H	ALA	A	10	-15.398	11.381	12.659
ATOM	5	CB	ALA	A	10	-14.683	11.830	15.045
ATOM	0	N	ILE	A	11	-14.372	14.489	13.445
ATOM	1	CA	ILE	A	11	-13.880	15.863	13.409
ATOM	2	C	ILE	A	11	-15.116	16.671	12.860
ATOM	3	O	ILE	A	11	-15.371	17.723	13.444
ATOM	4	H	ILE	A	11	-14.021	13.829	12.815
ATOM	5	CB	ILE	A	11	-12.672	15.995	12.464
ATOM	6	CG1	ILE	A	11	-11.471	15.207	12.962
ATOM	7	CD	ILE	A	11	-10.470	15.015	11.834
ATOM	8	CG2	ILE	A	11	-12.264	17.454	12.341
ATOM	0	N	ALA	A	12	-15.790	16.207	11.818
ATOM	1	CA	ALA	A	12	-16.921	16.939	11.274
ATOM	2	C	ALA	A	12	-17.978	17.140	12.339
ATOM	3	O	ALA	A	12	-18.511	18.232	12.458
ATOM	4	H	ALA	A	12	-15.524	15.361	11.409
ATOM	5	CB	ALA	A	12	-17.527	16.144	10.103

ATOM 0 N ALA A 13 -18.260 16.144 13.160
ATOM 1 CA ALA A 13 -19.234 16.359 14.246
ATOM 2 C ALA A 13 -18.839 17.390 15.248
ATOM 3 O ALA A 13 -19.584 18.237 15.708
ATOM 4 H ALA A 13 -17.827 15.274 13.050
ATOM 5 CB ALA A 13 -19.429 15.023 14.987
ATOM 0 N ASN A 14 -17.567 17.352 15.645
ATOM 1 CA ASN A 14 -17.046 18.336 16.567
ATOM 2 C ASN A 14 -16.912 19.784 16.160
ATOM 3 O ASN A 14 -17.264 20.720 16.879
ATOM 4 H ASN A 14 -16.977 16.650 15.308
ATOM 5 CB ASN A 14 -15.637 17.862 16.968
ATOM 6 CG ASN A 14 -15.683 16.659 17.896
ATOM 7 OD1 ASN A 14 -14.703 15.905 17.983
ATOM 8 ND2 ASN A 14 -16.839 16.528 18.561
ATOM 0 N LYS A 15 -16.392 19.960 14.950
ATOM 1 CA LYS A 15 -16.138 21.296 14.428
ATOM 2 C LYS A 15 -17.167 21.874 13.467
ATOM 3 O LYS A 15 -17.483 23.062 13.547
ATOM 4 H LYS A 15 -16.177 19.181 14.400
ATOM 5 CB LYS A 15 -14.787 21.258 13.690
ATOM 6 CG LYS A 15 -13.626 21.014 14.640
ATOM 7 CD LYS A 15 -12.290 21.001 13.916
ATOM 8 CE LYS A 15 -11.129 20.757 14.866
ATOM 9 NZ LYS A 15 -9.828 20.744 14.160
TER
ENDMDL
MODEL 4 KTSSFECIQAIAANK
ATOM 0 N LYS A 1 -8.337 -0.652 12.896
ATOM 1 CA LYS A 1 -8.309 -0.029 11.578
ATOM 2 C LYS A 1 -9.188 1.201 11.546
ATOM 3 O LYS A 1 -10.310 1.165 12.030
ATOM 4 H LYS A 1 -7.791 -1.495 13.073
ATOM 5 CB LYS A 1 -8.815 -1.037 10.530
ATOM 6 CG LYS A 1 -7.836 -2.180 10.319
ATOM 7 CD LYS A 1 -8.324 -3.162 9.267
ATOM 8 CE LYS A 1 -7.344 -4.305 9.057
ATOM 9 NZ LYS A 1 -7.820 -5.262 8.032
ATOM 0 N THR A 2 -8.651 2.282 10.990
ATOM 1 CA THR A 2 -9.466 3.480 10.741
ATOM 2 C THR A 2 -10.170 3.472 9.380
ATOM 3 O THR A 2 -9.623 2.989 8.377
ATOM 4 H THR A 2 -7.705 2.282 10.743

ATOM	5	CB	THR	A	2	-8.550	4.716	10.806
ATOM	6	OG1	THR	A	2	-7.928	4.796	12.091
ATOM	7	CG2	THR	A	2	-9.318	6.007	10.576
ATOM	0	N	SER	A	3	-11.404	3.970	9.392
ATOM	1	CA	SER	A	3	-12.218	4.158	8.195
ATOM	2	C	SER	A	3	-12.263	5.664	7.988
ATOM	3	O	SER	A	3	-12.580	6.423	8.897
ATOM	4	H	SER	A	3	-11.788	4.226	10.253
ATOM	5	CB	SER	A	3	-13.637	3.599	8.410
ATOM	6	OG	SER	A	3	-13.527	2.192	8.640
ATOM	0	N	SER	A	4	-11.916	6.084	6.785
ATOM	1	CA	SER	A	4	-11.731	7.496	6.488
ATOM	2	C	SER	A	4	-12.976	8.306	6.608
ATOM	3	O	SER	A	4	-12.866	9.444	7.079
ATOM	4	H	SER	A	4	-11.780	5.428	6.073
ATOM	5	CB	SER	A	4	-11.207	7.626	5.046
ATOM	6	OG	SER	A	4	-12.244	7.198	4.159
ATOM	0	N	PHE	A	5	-14.146	7.771	6.308
ATOM	1	CA	PHE	A	5	-15.382	8.545	6.388
ATOM	2	C	PHE	A	5	-15.752	8.600	7.821
ATOM	3	O	PHE	A	5	-16.238	9.665	8.205
ATOM	4	H	PHE	A	5	-14.187	6.836	6.027
ATOM	5	CB	PHE	A	5	-16.496	7.855	5.579
ATOM	6	CG	PHE	A	5	-16.249	7.942	4.081
ATOM	7	CD1	PHE	A	5	-15.589	6.930	3.436
ATOM	8	CE1	PHE	A	5	-15.366	7.008	2.087
ATOM	9	CZ	PHE	A	5	-15.805	8.099	1.382
ATOM	10	CE2	PHE	A	5	-16.465	9.111	2.028
ATOM	11	CD2	PHE	A	5	-16.688	9.032	3.377
ATOM	0	N	GLU	A	6	-15.567	7.560	8.626
ATOM	1	CA	GLU	A	6	-16.003	7.598	10.015
ATOM	2	C	GLU	A	6	-15.031	8.526	10.582
ATOM	3	O	GLU	A	6	-15.506	9.277	11.420
ATOM	4	H	GLU	A	6	-15.130	6.756	8.282
ATOM	5	CB	GLU	A	6	-15.912	6.210	10.677
ATOM	6	CG	GLU	A	6	-16.954	5.249	10.130
ATOM	7	CD	GLU	A	6	-16.834	3.865	10.748
ATOM	8	OE1	GLU	A	6	-17.679	2.996	10.491
ATOM	9	OE2	GLU	A	6	-15.889	3.607	11.509
ATOM	0	N	CYS	A	7	-13.768	8.625	10.208
ATOM	1	CA	CYS	A	7	-12.917	9.667	10.812
ATOM	2	C	CYS	A	7	-13.346	11.101	10.478
ATOM	3	O	CYS	A	7	-13.354	11.969	11.355

ATOM	4	H	CYS	A	7	-13.400	8.016	9.539
ATOM	5	CB	CYS	A	7	-11.475	9.467	10.311
ATOM	6	SG	CYS	A	7	-10.252	10.622	11.005
ATOM	0	N	ILE	A	8	-13.708	11.353	9.222
ATOM	1	CA	ILE	A	8	-14.111	12.701	8.834
ATOM	2	C	ILE	A	8	-15.430	12.972	9.576
ATOM	3	O	ILE	A	8	-15.571	14.096	10.033
ATOM	4	H	ILE	A	8	-13.706	10.635	8.559
ATOM	5	CB	ILE	A	8	-14.328	12.781	7.311
ATOM	6	CG1	ILE	A	8	-13.043	12.527	6.539
ATOM	7	CD	ILE	A	8	-13.364	12.200	5.090
ATOM	8	CG2	ILE	A	8	-14.846	14.158	6.931
ATOM	0	N	GLN	A	9	-16.341	12.034	9.762
ATOM	1	CA	GLN	A	9	-17.551	12.351	10.543
ATOM	2	C	GLN	A	9	-17.211	12.672	11.977
ATOM	3	O	GLN	A	9	-17.763	13.604	12.539
ATOM	4	H	GLN	A	9	-16.215	11.141	9.387
ATOM	5	CB	GLN	A	9	-18.500	11.138	10.513
ATOM	6	CG	GLN	A	9	-19.066	10.892	9.124
ATOM	7	CD	GLN	A	9	-19.954	9.659	9.082
ATOM	8	OE1	GLN	A	9	-20.029	8.915	10.071
ATOM	9	NE2	GLN	A	9	-20.657	9.377	7.976
ATOM	0	N	ALA	A	10	-16.274	11.963	12.579
ATOM	1	CA	ALA	A	10	-15.882	12.310	13.958
ATOM	2	C	ALA	A	10	-15.223	13.687	14.097
ATOM	3	O	ALA	A	10	-15.529	14.432	15.031
ATOM	4	H	ALA	A	10	-15.843	11.217	12.117
ATOM	5	CB	ALA	A	10	-14.888	11.247	14.461
ATOM	0	N	ILE	A	11	-14.329	14.032	13.172
ATOM	1	CA	ILE	A	11	-13.652	15.323	13.256
ATOM	2	C	ILE	A	11	-14.800	16.351	12.930
ATOM	3	O	ILE	A	11	-14.857	17.340	13.658
ATOM	4	H	ILE	A	11	-14.126	13.422	12.437
ATOM	5	CB	ILE	A	11	-12.515	15.420	12.223
ATOM	6	CG1	ILE	A	11	-11.403	14.423	12.505
ATOM	7	CD	ILE	A	11	-10.531	14.254	11.271
ATOM	8	CG2	ILE	A	11	-11.916	16.817	12.240
ATOM	0	N	ALA	A	12	-15.613	16.122	11.909
ATOM	1	CA	ALA	A	12	-16.668	17.061	11.568
ATOM	2	C	ALA	A	12	-17.535	17.326	12.770
ATOM	3	O	ALA	A	12	-17.793	18.446	13.204
ATOM	4	H	ALA	A	12	-15.502	15.310	11.377
ATOM	5	CB	ALA	A	12	-17.532	16.470	10.439

ATOM 0 N ALA A 13 -17.939 16.245 13.397
ATOM 1 CA ALA A 13 -18.791 16.410 14.547
ATOM 2 C ALA A 13 -18.133 17.180 15.656
ATOM 3 O ALA A 13 -18.727 18.046 16.279
ATOM 4 H ALA A 13 -17.670 15.356 13.093
ATOM 5 CB ALA A 13 -19.175 15.017 15.079
ATOM 0 N ASN A 14 -16.856 16.927 15.879
ATOM 1 CA ASN A 14 -16.133 17.711 16.896
ATOM 2 C ASN A 14 -15.851 19.185 16.697
ATOM 3 O ASN A 14 -16.019 20.027 17.580
ATOM 4 H ASN A 14 -16.395 16.229 15.374
ATOM 5 CB ASN A 14 -14.765 17.029 17.085
ATOM 6 CG ASN A 14 -14.885 15.710 17.832
ATOM 7 OD1 ASN A 14 -14.001 14.848 17.714
ATOM 8 ND2 ASN A 14 -15.991 15.607 18.582
ATOM 0 N LYS A 15 -15.424 19.493 15.478
ATOM 1 CA LYS A 15 -15.055 20.860 15.134
ATOM 2 C LYS A 15 -16.086 21.691 14.385
ATOM 3 O LYS A 15 -16.244 22.878 14.671
ATOM 4 H LYS A 15 -15.357 18.793 14.799
ATOM 5 CB LYS A 15 -13.790 20.793 14.258
ATOM 6 CG LYS A 15 -12.588 20.277 15.031
ATOM 7 CD LYS A 15 -11.334 20.234 14.173
ATOM 8 CE LYS A 15 -10.132 19.718 14.947
ATOM 9 NZ LYS A 15 -8.911 19.676 14.111
TER
ENDMDL
MODEL 5 KTSSFECIQAIAANK
ATOM 0 N LYS A 1 -7.981 -0.210 12.131
ATOM 1 CA LYS A 1 -7.912 1.218 11.875
ATOM 2 C LYS A 1 -9.308 1.780 11.654
ATOM 3 O LYS A 1 -10.137 1.107 11.065
ATOM 4 H LYS A 1 -7.126 -0.740 12.300
ATOM 5 CB LYS A 1 -7.059 1.471 10.619
ATOM 6 CG LYS A 1 -5.589 1.167 10.859
ATOM 7 CD LYS A 1 -4.742 1.440 9.626
ATOM 8 CE LYS A 1 -3.272 1.136 9.866
ATOM 9 NZ LYS A 1 -2.448 1.402 8.665
ATOM 0 N THR A 2 -9.579 3.031 12.104
ATOM 1 CA THR A 2 -10.803 3.653 11.659
ATOM 2 C THR A 2 -10.798 3.834 10.145
ATOM 3 O THR A 2 -9.745 4.033 9.542
ATOM 4 H THR A 2 -8.967 3.496 12.708

```
ATOM      5  CB  THR A   2     -10.944   5.032  12.331
ATOM      6  OG1 THR A   2     -10.991   4.881  13.752
ATOM      7  CG2 THR A   2     -12.210   5.751  11.894
ATOM      0  N   SER A   3     -11.980   3.728   9.567
ATOM      1  CA  SER A   3     -12.208   4.043   8.167
ATOM      2  C   SER A   3     -12.110   5.533   7.980
ATOM      3  O   SER A   3     -12.528   6.302   8.844
ATOM      4  H   SER A   3     -12.738   3.425  10.106
ATOM      5  CB  SER A   3     -13.609   3.561   7.748
ATOM      6  OG  SER A   3     -13.650   2.140   7.895
ATOM      0  N   SER A   4     -11.615   5.944   6.823
ATOM      1  CA  SER A   4     -11.510   7.354   6.479
ATOM      2  C   SER A   4     -12.765   8.147   6.452
ATOM      3  O   SER A   4     -12.688   9.362   6.646
ATOM      4  H   SER A   4     -11.309   5.278   6.177
ATOM      5  CB  SER A   4     -10.880   7.443   5.076
ATOM      6  OG  SER A   4     -11.825   6.930   4.134
ATOM      0  N   PHE A   5     -13.916   7.473   6.377
ATOM      1  CA  PHE A   5     -15.217   8.182   6.354
ATOM      2  C   PHE A   5     -15.629   8.468   7.788
ATOM      3  O   PHE A   5     -16.112   9.555   8.106
ATOM      4  H   PHE A   5     -13.901   6.497   6.336
ATOM      5  CB  PHE A   5     -16.280   7.300   5.674
ATOM      6  CG  PHE A   5     -16.029   7.153   4.182
ATOM      7  CD1 PHE A   5     -15.303   6.091   3.711
ATOM      8  CE1 PHE A   5     -15.076   5.959   2.366
ATOM      9  CZ  PHE A   5     -15.577   6.888   1.493
ATOM     10  CE2 PHE A   5     -16.303   7.950   1.964
ATOM     11  CD2 PHE A   5     -16.529   8.082   3.309
ATOM      0  N   GLU A   6     -15.429   7.485   8.659
ATOM      1  CA  GLU A   6     -15.846   7.615  10.049
ATOM      2  C   GLU A   6     -14.868   8.582  10.538
ATOM      3  O   GLU A   6     -15.334   9.386  11.330
ATOM      4  H   GLU A   6     -14.995   6.660   8.363
ATOM      5  CB  GLU A   6     -15.742   6.276  10.801
ATOM      6  CG  GLU A   6     -16.789   5.278  10.335
ATOM      7  CD  GLU A   6     -16.656   3.939  11.043
ATOM      8  OE1 GLU A   6     -17.503   3.053  10.857
ATOM      9  OE2 GLU A   6     -15.701   3.734  11.806
ATOM      0  N   CYS A   7     -13.611   8.657  10.140
ATOM      1  CA  CYS A   7     -12.755   9.739  10.660
ATOM      2  C   CYS A   7     -13.192  11.146  10.237
ATOM      3  O   CYS A   7     -13.190  12.071  11.053
```

ATOM	4	H	CYS	A	7	-13.252	8.004	9.508
ATOM	5	CB	CYS	A	7	-11.320	9.508	10.153
ATOM	6	SG	CYS	A	7	-10.089	10.709	10.750
ATOM	0	N	ILE	A	8	-13.573	11.312	8.971
ATOM	1	CA	ILE	A	8	-13.985	12.631	8.499
ATOM	2	C	ILE	A	8	-15.323	13.014	9.201
ATOM	3	O	ILE	A	8	-15.440	14.186	9.567
ATOM	4	H	ILE	A	8	-13.578	10.551	8.358
ATOM	5	CB	ILE	A	8	-14.192	12.596	6.973
ATOM	6	CG1	ILE	A	8	-12.907	12.266	6.231
ATOM	7	CD	ILE	A	8	-13.198	12.061	4.753
ATOM	8	CG2	ILE	A	8	-14.689	13.947	6.485
ATOM	0	N	GLN	A	9	-16.281	12.108	9.390
ATOM	1	CA	GLN	A	9	-17.535	12.494	10.029
ATOM	2	C	GLN	A	9	-17.110	12.764	11.521
ATOM	3	O	GLN	A	9	-17.571	13.781	12.034
ATOM	4	H	GLN	A	9	-16.144	11.184	9.102
ATOM	5	CB	GLN	A	9	-18.571	11.357	9.948
ATOM	6	CG	GLN	A	9	-19.001	11.080	8.516
ATOM	7	CD	GLN	A	9	-19.986	9.925	8.432
ATOM	8	OE1	GLN	A	9	-20.299	9.300	9.456
ATOM	9	NE2	GLN	A	9	-20.517	9.583	7.250
ATOM	0	N	ALA	A	10	-16.317	11.902	12.141
ATOM	1	CA	ALA	A	10	-15.924	12.111	13.524
ATOM	2	C	ALA	A	10	-15.333	13.543	13.626
ATOM	3	O	ALA	A	10	-15.757	14.246	14.548
ATOM	4	H	ALA	A	10	-15.992	11.115	11.662
ATOM	5	CB	ALA	A	10	-14.862	11.073	13.934
ATOM	0	N	ILE	A	11	-14.429	13.960	12.743
ATOM	1	CA	ILE	A	11	-13.817	15.285	12.801
ATOM	2	C	ILE	A	11	-14.862	16.403	12.708
ATOM	3	O	ILE	A	11	-14.806	17.363	13.480
ATOM	4	H	ILE	A	11	-14.164	13.355	12.023
ATOM	5	CB	ILE	A	11	-12.829	15.430	11.629
ATOM	6	CG1	ILE	A	11	-12.017	16.712	11.724
ATOM	7	CD	ILE	A	11	-10.859	16.668	10.740
ATOM	8	CG2	ILE	A	11	-13.585	15.445	10.310
ATOM	0	N	ALA	A	12	-15.811	16.288	11.780
ATOM	1	CA	ALA	A	12	-16.819	17.334	11.632
ATOM	2	C	ALA	A	12	-17.662	17.285	12.917
ATOM	3	O	ALA	A	12	-17.996	18.362	13.385
ATOM	4	H	ALA	A	12	-15.833	15.503	11.198
ATOM	5	CB	ALA	A	12	-17.702	17.059	10.401

```
ATOM     0  N   ALA A  13     -17.966  16.149  13.519
ATOM     1  CA  ALA A  13     -18.721  16.185  14.785
ATOM     2  C   ALA A  13     -17.973  16.937  15.847
ATOM     3  O   ALA A  13     -18.444  17.847  16.524
ATOM     4  H   ALA A  13     -17.694  15.295  13.131
ATOM     5  CB  ALA A  13     -18.960  14.743  15.269
ATOM     0  N   ASN A  14     -16.708  16.600  15.948
ATOM     1  CA  ASN A  14     -15.914  17.251  16.958
ATOM     2  C   ASN A  14     -15.612  18.736  16.933
ATOM     3  O   ASN A  14     -15.700  19.491  17.902
ATOM     4  H   ASN A  14     -16.317  15.928  15.356
ATOM     5  CB  ASN A  14     -14.548  16.540  16.952
ATOM     6  CG  ASN A  14     -14.650  15.106  17.444
ATOM     7  OD1 ASN A  14     -15.422  14.822  18.372
ATOM     8  ND2 ASN A  14     -13.855  14.247  16.792
ATOM     0  N   LYS A  15     -15.270  19.157  15.720
ATOM     1  CA  LYS A  15     -14.905  20.547  15.482
ATOM     2  C   LYS A  15     -15.974  21.456  14.895
ATOM     3  O   LYS A  15     -16.092  22.611  15.305
ATOM     4  H   LYS A  15     -15.265  18.525  14.975
ATOM     5  CB  LYS A  15     -13.709  20.553  14.511
ATOM     6  CG  LYS A  15     -12.462  19.953  15.140
ATOM     7  CD  LYS A  15     -11.276  19.982  14.190
ATOM     8  CE  LYS A  15     -10.028  19.382  14.819
ATOM     9  NZ  LYS A  15      -8.873  19.410  13.894
TER
ENDMDL
```

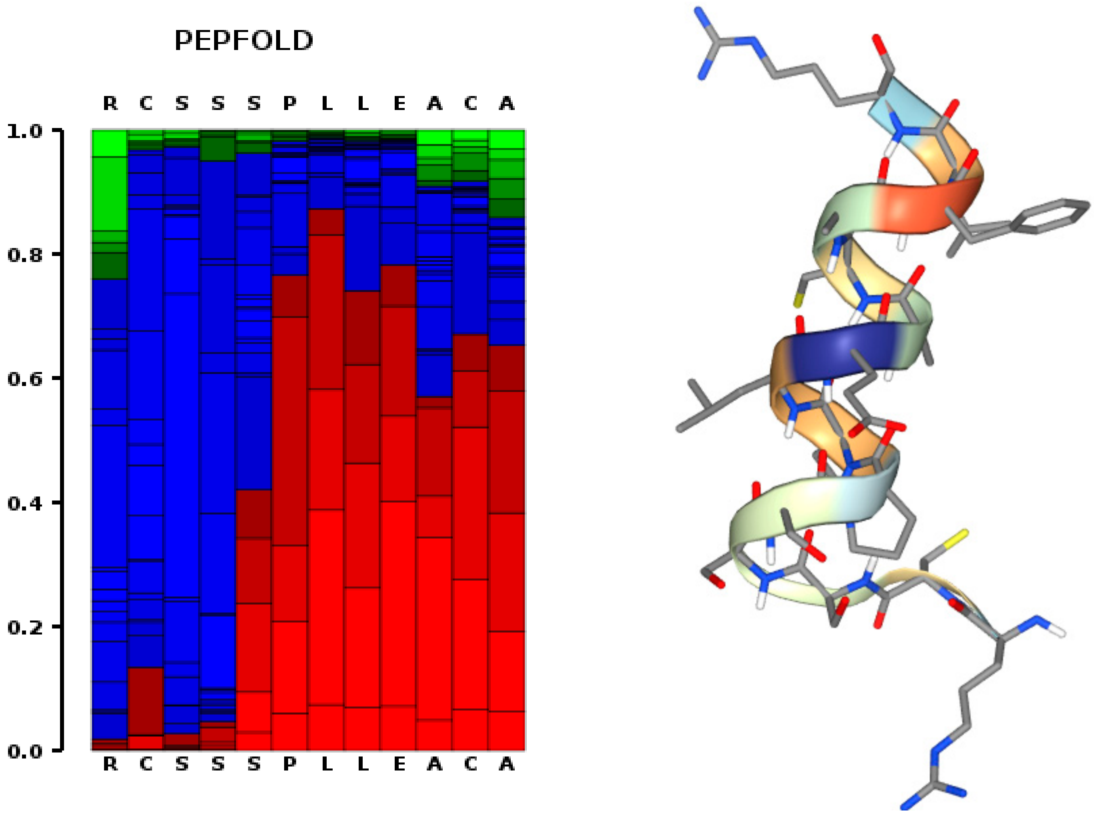

Figure S4 3D structure of peptide and predicted local structure profile RCSSSPLLEACAFLR job execution pep-fold4.py --maxSimSze 50 --maxSeqSze 50 --noMQA --sortKey sOPEP -s iSeq.fasta -l PEPFOLD --generator fbt --nRuns 100 --mcSteps 30000 --mcT 370 --seed 1 Debye-Huckel formalism Solvent pH 7.5 Ionic strength (mM) 150

Table S5 5 best Models *(Protein _3DStructure)* RCSSSPLLEACAFLR

MODEL 1

ATOM	0	N	ARG	A	1	-38.691	-5.794	16.002
ATOM	1	CA	ARG	A	1	-39.460	-5.461	14.807
ATOM	2	C	ARG	A	1	-39.114	-6.401	13.658
ATOM	3	O	ARG	A	1	-38.209	-6.129	12.870
ATOM	4	H	ARG	A	1	-38.815	-5.250	16.856
ATOM	5	CB	ARG	A	1	-39.139	-4.014	14.387
ATOM	6	CG	ARG	A	1	-39.990	-3.561	13.212
ATOM	7	CD	ARG	A	1	-40.128	-2.048	13.158
ATOM	8	NE	ARG	A	1	-41.044	-1.616	12.094
ATOM	9	CZ	ARG	A	1	-41.285	-0.302	11.902
ATOM	10	NH1	ARG	A	1	-42.121	0.092	10.932
ATOM	11	NH2	ARG	A	1	-40.688	0.609	12.683
ATOM	0	N	CYS	A	2	-39.841	-7.510	13.569
ATOM	1	CA	CYS	A	2	-39.611	-8.492	12.516
ATOM	2	C	CYS	A	2	-40.124	-7.980	11.173
ATOM	3	O	CYS	A	2	-41.301	-7.650	11.031

ATOM	4	H	CYS	A	2	-40.548	-7.672	14.224
ATOM	5	CB	CYS	A	2	-40.347	-9.798	12.871
ATOM	6	SG	CYS	A	2	-39.742	-10.638	14.368
ATOM	0	N	SER	A	3	-39.232	-7.920	10.189
ATOM	1	CA	SER	A	3	-39.591	-7.446	8.858
ATOM	2	C	SER	A	3	-38.841	-8.225	7.782
ATOM	3	O	SER	A	3	-38.031	-9.100	8.086
ATOM	4	H	SER	A	3	-38.312	-8.201	10.362
ATOM	5	CB	SER	A	3	-39.234	-5.953	8.739
ATOM	6	OG	SER	A	3	-37.812	-5.831	8.822
ATOM	0	N	SER	A	4	-39.119	-7.901	6.523
ATOM	1	CA	SER	A	4	-38.471	-8.571	5.402
ATOM	2	C	SER	A	4	-36.919	-8.773	5.892
ATOM	3	O	SER	A	4	-36.311	-9.842	5.902
ATOM	4	H	SER	A	4	-39.772	-7.196	6.344
ATOM	5	CB	SER	A	4	-38.532	-7.699	4.134
ATOM	6	OG	SER	A	4	-37.807	-6.494	4.392
ATOM	0	N	SER	A	5	-36.307	-7.602	6.059
ATOM	1	CA	SER	A	5	-34.870	-7.540	6.242
ATOM	2	C	SER	A	5	-34.320	-8.110	7.550
ATOM	3	O	SER	A	5	-33.307	-8.811	7.547
ATOM	4	H	SER	A	5	-36.831	-6.777	6.060
ATOM	5	CB	SER	A	5	-34.452	-6.059	6.167
ATOM	6	OG	SER	A	5	-34.978	-5.393	7.318
ATOM	0	N	PRO	A	6	-34.994	-7.821	8.658
ATOM	1	CA	PRO	A	6	-34.522	-8.265	9.962
ATOM	2	C	PRO	A	6	-34.761	-9.799	9.924
ATOM	3	O	PRO	A	6	-33.922	-10.505	10.454
ATOM	4	CB	PRO	A	6	-35.366	-7.569	11.046
ATOM	5	CG	PRO	A	6	-35.871	-6.288	10.357
ATOM	6	CD	PRO	A	6	-35.998	-6.705	8.889
ATOM	0	N	LEU	A	7	-35.823	-10.365	9.348
ATOM	1	CA	LEU	A	7	-35.960	-11.803	9.292
ATOM	2	C	LEU	A	7	-34.884	-12.495	8.453
ATOM	3	O	LEU	A	7	-34.348	-13.528	8.860
ATOM	4	H	LEU	A	7	-36.518	-9.800	8.957
ATOM	5	CB	LEU	A	7	-37.336	-12.137	8.687
ATOM	6	CG	LEU	A	7	-38.473	-11.802	9.638
ATOM	7	CD1	LEU	A	7	-39.829	-11.883	8.955
ATOM	8	CD2	LEU	A	7	-38.516	-12.746	10.829
ATOM	0	N	LEU	A	8	-34.562	-11.934	7.288
ATOM	1	CA	LEU	A	8	-33.560	-12.558	6.430
ATOM	2	C	LEU	A	8	-32.172	-12.456	7.133

ATOM 3 O LEU A 8 -31.437 -13.444 7.053
ATOM 4 H LEU A 8 -34.997 -11.105 7.008
ATOM 5 CB LEU A 8 -33.508 -11.828 5.075
ATOM 6 CG LEU A 8 -34.792 -12.009 4.281
ATOM 7 CD1 LEU A 8 -34.805 -11.164 3.018
ATOM 8 CD2 LEU A 8 -34.999 -13.454 3.855
ATOM 0 N GLU A 9 -31.814 -11.351 7.785
ATOM 1 CA GLU A 9 -30.500 -11.268 8.416
ATOM 2 C GLU A 9 -30.617 -12.276 9.620
ATOM 3 O GLU A 9 -29.660 -13.029 9.785
ATOM 4 H GLU A 9 -32.430 -10.595 7.841
ATOM 5 CB GLU A 9 -30.215 -9.840 8.917
ATOM 6 CG GLU A 9 -30.020 -8.863 7.769
ATOM 7 CD GLU A 9 -29.787 -7.444 8.263
ATOM 8 OE1 GLU A 9 -29.500 -6.546 7.457
ATOM 9 OE2 GLU A 9 -29.884 -7.185 9.472
ATOM 0 N ALA A 10 -31.697 -12.254 10.389
ATOM 1 CA ALA A 10 -31.826 -13.155 11.521
ATOM 2 C ALA A 10 -31.578 -14.595 10.997
ATOM 3 O ALA A 10 -30.787 -15.285 11.646
ATOM 4 H ALA A 10 -32.417 -11.623 10.192
ATOM 5 CB ALA A 10 -33.241 -13.051 12.121
ATOM 0 N CYS A 11 -32.192 -15.024 9.897
ATOM 1 CA CYS A 11 -32.021 -16.375 9.369
ATOM 2 C CYS A 11 -30.557 -16.633 9.160
ATOM 3 O CYS A 11 -29.949 -17.596 9.621
ATOM 4 H CYS A 11 -32.783 -14.409 9.421
ATOM 5 CB CYS A 11 -32.764 -16.506 8.027
ATOM 6 SG CYS A 11 -34.558 -16.781 8.165
ATOM 0 N ALA A 12 -29.930 -15.689 8.496
ATOM 1 CA ALA A 12 -28.527 -15.871 8.225
ATOM 2 C ALA A 12 -27.676 -16.048 9.574
ATOM 3 O ALA A 12 -26.889 -16.964 9.797
ATOM 4 H ALA A 12 -30.401 -14.887 8.195
ATOM 5 CB ALA A 12 -28.001 -14.640 7.464
ATOM 0 N PHE A 13 -27.970 -15.165 10.500
ATOM 1 CA PHE A 13 -27.257 -15.233 11.750
ATOM 2 C PHE A 13 -27.478 -16.528 12.479
ATOM 3 O PHE A 13 -26.561 -17.130 13.015
ATOM 4 H PHE A 13 -28.652 -14.482 10.347
ATOM 5 CB PHE A 13 -27.729 -14.076 12.650
ATOM 6 CG PHE A 13 -27.067 -14.113 14.018
ATOM 7 CD1 PHE A 13 -25.776 -13.677 14.165

ATOM 8 CE1 PHE A 13 -25.179 -13.709 15.398
ATOM 9 CZ PHE A 13 -25.872 -14.178 16.483
ATOM 10 CE2 PHE A 13 -27.162 -14.614 16.335
ATOM 11 CD2 PHE A 13 -27.760 -14.582 15.103
ATOM 0 N LEU A 14 -28.703 -17.023 12.451
ATOM 1 CA LEU A 14 -28.965 -18.332 13.076
ATOM 2 C LEU A 14 -28.250 -19.507 12.398
ATOM 3 O LEU A 14 -27.706 -20.381 13.077
ATOM 4 H LEU A 14 -29.427 -16.528 12.021
ATOM 5 CB LEU A 14 -30.481 -18.598 13.026
ATOM 6 CG LEU A 14 -31.270 -17.533 13.770
ATOM 7 CD1 LEU A 14 -32.764 -17.654 13.520
ATOM 8 CD2 LEU A 14 -31.059 -17.613 15.273
ATOM 0 N ARG A 15 -28.240 -19.529 11.066
ATOM 1 CA ARG A 15 -27.592 -20.629 10.358
ATOM 2 C ARG A 15 -26.069 -20.520 10.401
ATOM 3 O ARG A 15 -25.370 -21.380 9.859
ATOM 4 H ARG A 15 -28.667 -18.809 10.561
ATOM 5 CB ARG A 15 -28.048 -20.614 8.888
ATOM 6 CG ARG A 15 -27.511 -21.806 8.112
ATOM 7 CD ARG A 15 -28.371 -22.133 6.903
ATOM 8 NE ARG A 15 -27.920 -23.354 6.220
ATOM 9 CZ ARG A 15 -28.572 -23.794 5.123
ATOM 10 NH1 ARG A 15 -28.161 -24.908 4.501
ATOM 11 NH2 ARG A 15 -29.630 -23.117 4.655
TER
ENDMDL
MODEL 2 **RCSSSPLLEACAFLR**
ATOM 0 N ARG A 1 -39.453 -1.599 12.284
ATOM 1 CA ARG A 1 -40.735 -2.253 12.533
ATOM 2 C ARG A 1 -41.097 -3.283 11.486
ATOM 3 O ARG A 1 -42.243 -3.758 11.388
ATOM 4 H ARG A 1 -39.111 -0.887 12.928
ATOM 5 CB ARG A 1 -41.837 -1.178 12.559
ATOM 6 CG ARG A 1 -43.187 -1.755 12.950
ATOM 7 CD ARG A 1 -44.109 -0.699 13.538
ATOM 8 NE ARG A 1 -45.370 -1.278 14.024
ATOM 9 CZ ARG A 1 -46.308 -0.482 14.579
ATOM 10 NH1 ARG A 1 -47.458 -1.010 15.022
ATOM 11 NH2 ARG A 1 -46.091 0.836 14.686
ATOM 0 N CYS A 2 -40.105 -3.680 10.695
ATOM 1 CA CYS A 2 -40.365 -4.621 9.619
ATOM 2 C CYS A 2 -39.803 -5.995 9.967

ATOM	3	O	CYS	A	2	-38.624	-6.132	10.378
ATOM	4	H	CYS	A	2	-39.200	-3.340	10.837
ATOM	5	CB	CYS	A	2	-39.698	-4.114	8.327
ATOM	6	SG	CYS	A	2	-40.368	-2.551	7.677
ATOM	0	N	SER	A	3	-40.663	-6.992	9.803
ATOM	1	CA	SER	A	3	-40.310	-8.370	10.121
ATOM	2	C	SER	A	3	-39.299	-8.933	9.104
ATOM	3	O	SER	A	3	-38.410	-9.676	9.505
ATOM	4	H	SER	A	3	-41.557	-6.798	9.459
ATOM	5	CB	SER	A	3	-41.584	-9.236	10.093
ATOM	6	OG	SER	A	3	-42.070	-9.263	8.749
ATOM	0	N	SER	A	4	-39.452	-8.612	7.826
ATOM	1	CA	SER	A	4	-38.606	-9.209	6.808
ATOM	2	C	SER	A	4	-37.120	-9.123	7.138
ATOM	3	O	SER	A	4	-36.434	-10.136	7.017
ATOM	4	H	SER	A	4	-40.139	-7.967	7.566
ATOM	5	CB	SER	A	4	-38.853	-8.481	5.474
ATOM	6	OG	SER	A	4	-38.380	-7.138	5.609
ATOM	0	N	SER	A	5	-36.619	-7.963	7.549
ATOM	1	CA	SER	A	5	-35.206	-7.851	7.868
ATOM	2	C	SER	A	5	-34.705	-8.656	9.078
ATOM	3	O	SER	A	5	-33.633	-9.256	8.997
ATOM	4	H	SER	A	5	-37.202	-7.184	7.638
ATOM	5	CB	SER	A	5	-34.904	-6.366	8.140
ATOM	6	OG	SER	A	5	-35.564	-5.996	9.353
ATOM	0	N	PRO	A	6	-35.464	-8.677	10.169
ATOM	1	CA	PRO	A	6	-35.025	-9.364	11.376
ATOM	2	C	PRO	A	6	-35.117	-10.864	10.985
ATOM	3	O	PRO	A	6	-34.247	-11.602	11.408
ATOM	4	CB	PRO	A	6	-35.988	-8.998	12.520
ATOM	5	CG	PRO	A	6	-36.573	-7.639	12.092
ATOM	6	CD	PRO	A	6	-36.578	-7.723	10.563
ATOM	0	N	LEU	A	7	-36.088	-11.368	10.221
ATOM	1	CA	LEU	A	7	-36.088	-12.762	9.842
ATOM	2	C	LEU	A	7	-34.851	-13.107	9.069
ATOM	3	O	LEU	A	7	-34.102	-14.045	9.329
ATOM	4	H	LEU	A	7	-36.810	-10.786	9.913
ATOM	5	CB	LEU	A	7	-37.323	-13.049	8.968
ATOM	6	CG	LEU	A	7	-38.621	-12.786	9.714
ATOM	7	CD1	LEU	A	7	-39.827	-12.832	8.790
ATOM	8	CD2	LEU	A	7	-38.862	-13.806	10.815
ATOM	0	N	LEU	A	8	-34.569	-12.259	8.107
ATOM	1	CA	LEU	A	8	-33.414	-12.525	7.289

```
ATOM      2  C   LEU A   8      -32.070 -12.539   8.020
ATOM      3  O   LEU A   8      -31.216 -13.403   7.809
ATOM      4  H   LEU A   8      -35.126 -11.471   7.951
ATOM      5  CB  LEU A   8      -33.346 -11.441   6.197
ATOM      6  CG  LEU A   8      -32.120 -11.599   5.313
ATOM      7  CD1 LEU A   8      -32.131 -12.917   4.555
ATOM      8  CD2 LEU A   8      -32.015 -10.489   4.280
ATOM      0  N   GLU A   9      -31.898 -11.568   8.916
ATOM      1  CA  GLU A   9      -30.652 -11.486   9.673
ATOM      2  C   GLU A   9      -30.583 -12.764  10.504
ATOM      3  O   GLU A   9      -29.562 -13.446  10.534
ATOM      4  H   GLU A   9      -32.605 -10.911   9.068
ATOM      5  CB  GLU A   9      -30.663 -10.250  10.592
ATOM      6  CG  GLU A   9      -30.622  -8.952   9.802
ATOM      7  CD  GLU A   9      -30.682  -7.733  10.707
ATOM      8  OE1 GLU A   9      -30.546  -6.597  10.227
ATOM      9  OE2 GLU A   9      -30.866  -7.873  11.925
ATOM      0  N   ALA A  10      -31.674 -13.146  11.126
ATOM      1  CA  ALA A  10      -31.608 -14.332  11.941
ATOM      2  C   ALA A  10      -31.257 -15.563  11.153
ATOM      3  O   ALA A  10      -30.454 -16.385  11.567
ATOM      4  H   ALA A  10      -32.507 -12.641  11.040
ATOM      5  CB  ALA A  10      -32.979 -14.551  12.606
ATOM      0  N   CYS A  11      -31.804 -15.678   9.957
ATOM      1  CA  CYS A  11      -31.433 -16.820   9.101
ATOM      2  C   CYS A  11      -29.976 -16.767   8.717
ATOM      3  O   CYS A  11      -29.305 -17.787   8.731
ATOM      4  H   CYS A  11      -32.448 -15.014   9.641
ATOM      5  CB  CYS A  11      -32.289 -16.789   7.822
ATOM      6  SG  CYS A  11      -34.063 -17.110   8.075
ATOM      0  N   ALA A  12      -29.440 -15.596   8.424
ATOM      1  CA  ALA A  12      -28.001 -15.519   8.113
ATOM      2  C   ALA A  12      -27.161 -15.899   9.307
ATOM      3  O   ALA A  12      -26.180 -16.612   9.159
ATOM      4  H   ALA A  12      -29.991 -14.789   8.413
ATOM      5  CB  ALA A  12      -27.653 -14.079   7.694
ATOM      0  N   PHE A  13      -27.541 -15.491  10.504
ATOM      1  CA  PHE A  13      -26.769 -15.912  11.687
ATOM      2  C   PHE A  13      -26.837 -17.405  11.886
ATOM      3  O   PHE A  13      -25.832 -18.026  12.193
ATOM      4  H   PHE A  13      -28.326 -14.918  10.606
ATOM      5  CB  PHE A  13      -27.340 -15.215  12.936
ATOM      6  CG  PHE A  13      -26.629 -15.649  14.207
```

```
ATOM      7  CD1 PHE A  13     -25.395 -15.139  14.510
ATOM      8  CE1 PHE A  13     -24.754 -15.530  15.655
ATOM      9  CZ  PHE A  13     -25.347 -16.433  16.498
ATOM     10  CE2 PHE A  13     -26.582 -16.944  16.195
ATOM     11  CD2 PHE A  13     -27.223 -16.552  15.049
ATOM      0  N   LEU A  14     -27.985 -18.021  11.666
ATOM      1  CA  LEU A  14     -28.051 -19.489  11.789
ATOM      2  C   LEU A  14     -27.208 -20.245  10.755
ATOM      3  O   LEU A  14     -26.531 -21.218  11.096
ATOM      4  H   LEU A  14     -28.783 -17.510  11.424
ATOM      5  CB  LEU A  14     -29.519 -19.924  11.624
ATOM      6  CG  LEU A  14     -30.419 -19.302  12.679
ATOM      7  CD1 LEU A  14     -31.891 -19.530  12.377
ATOM      8  CD2 LEU A  14     -30.152 -19.872  14.062
ATOM      0  N   ARG A  15     -27.237 -19.800   9.500
ATOM      1  CA  ARG A  15     -26.471 -20.485   8.464
ATOM      2  C   ARG A  15     -24.976 -20.192   8.568
ATOM      3  O   ARG A  15     -24.186 -20.706   7.772
ATOM      4  H   ARG A  15     -27.772 -19.015   9.272
ATOM      5  CB  ARG A  15     -26.971 -20.021   7.084
ATOM      6  CG  ARG A  15     -26.305 -20.783   5.950
ATOM      7  CD  ARG A  15     -27.152 -20.782   4.688
ATOM      8  NE  ARG A  15     -26.564 -21.616   3.630
ATOM      9  CZ  ARG A  15     -27.186 -21.730   2.437
ATOM     10  NH1 ARG A  15     -26.650 -22.490   1.472
ATOM     11  NH2 ARG A  15     -28.338 -21.082   2.217
TER
ENDMDL
MODEL        3      RCSSSPLLEACAFLR
ATOM      0  N   ARG A   1     -35.712  -9.088  18.026
ATOM      1  CA  ARG A   1     -36.605  -8.612  16.975
ATOM      2  C   ARG A   1     -35.991  -8.838  15.597
ATOM      3  O   ARG A   1     -34.773  -8.794  15.433
ATOM      4  H   ARG A   1     -35.986  -9.004  19.005
ATOM      5  CB  ARG A   1     -36.861  -7.106  17.171
ATOM      6  CG  ARG A   1     -37.665  -6.824  18.430
ATOM      7  CD  ARG A   1     -37.970  -5.344  18.591
ATOM      8  NE  ARG A   1     -38.697  -5.064  19.838
ATOM      9  CZ  ARG A   1     -39.005  -3.792  20.169
ATOM     10  NH1 ARG A   1     -39.668  -3.537  21.305
ATOM     11  NH2 ARG A   1     -38.649  -2.785  19.360
ATOM      0  N   CYS A   2     -36.846  -9.078  14.609
ATOM      1  CA  CYS A   2     -36.390  -9.314  13.244
```

ATOM 2 C CYS A 2 -37.116 -8.400 12.261
ATOM 3 O CYS A 2 -38.347 -8.350 12.233
ATOM 4 H CYS A 2 -37.804 -9.096 14.800
ATOM 5 CB CYS A 2 -36.668 -10.781 12.865
ATOM 6 SG CYS A 2 -35.935 -12.016 13.982
ATOM 0 N SER A 3 -36.345 -7.678 11.454
ATOM 1 CA SER A 3 -36.913 -6.765 10.470
ATOM 2 C SER A 3 -37.874 -7.500 9.539
ATOM 3 O SER A 3 -38.156 -8.681 9.730
ATOM 4 H SER A 3 -35.374 -7.761 11.522
ATOM 5 CB SER A 3 -35.775 -6.150 9.634
ATOM 6 OG SER A 3 -34.938 -5.395 10.515
ATOM 0 N SER A 4 -38.373 -6.788 8.531
ATOM 1 CA SER A 4 -39.302 -7.372 7.572
ATOM 2 C SER A 4 -38.478 -8.006 6.449
ATOM 3 O SER A 4 -38.953 -8.901 5.745
ATOM 4 H SER A 4 -38.110 -5.852 8.431
ATOM 5 CB SER A 4 -40.218 -6.278 6.994
ATOM 6 OG SER A 4 -39.402 -5.359 6.263
ATOM 0 N SER A 5 -37.225 -7.582 6.319
ATOM 1 CA SER A 5 -36.367 -8.144 5.289
ATOM 2 C SER A 5 -36.138 -9.684 5.198
ATOM 3 O SER A 5 -36.075 -10.286 6.276
ATOM 4 H SER A 5 -36.879 -6.891 6.917
ATOM 5 CB SER A 5 -34.979 -7.504 5.474
ATOM 6 OG SER A 5 -34.411 -8.021 6.681
ATOM 0 N PRO A 6 -36.001 -10.305 4.022
ATOM 1 CA PRO A 6 -35.868 -11.779 4.048
ATOM 2 C PRO A 6 -34.427 -12.199 4.410
ATOM 3 O PRO A 6 -34.288 -13.207 5.111
ATOM 4 CB PRO A 6 -36.237 -12.275 2.637
ATOM 5 CG PRO A 6 -37.072 -11.124 2.046
ATOM 6 CD PRO A 6 -36.477 -9.880 2.712
ATOM 0 N LEU A 7 -33.400 -11.490 3.973
ATOM 1 CA LEU A 7 -32.024 -11.933 4.180
ATOM 2 C LEU A 7 -31.968 -11.547 5.608
ATOM 3 O LEU A 7 -31.387 -12.374 6.309
ATOM 4 H LEU A 7 -33.565 -10.650 3.502
ATOM 5 CB LEU A 7 -31.010 -11.164 3.313
ATOM 6 CG LEU A 7 -31.158 -11.493 1.836
ATOM 7 CD1 LEU A 7 -30.333 -10.565 0.960
ATOM 8 CD2 LEU A 7 -30.717 -12.914 1.523
ATOM 0 N LEU A 8 -32.476 -10.407 6.056

ATOM	1	CA	LEU	A	8	-32.378	-10.045	7.459
ATOM	2	C	LEU	A	8	-33.030	-11.105	8.321
ATOM	3	O	LEU	A	8	-32.464	-11.488	9.333
ATOM	4	H	LEU	A	8	-32.923	-9.801	5.433
ATOM	5	CB	LEU	A	8	-33.087	-8.698	7.689
ATOM	6	CG	LEU	A	8	-32.442	-7.572	6.897
ATOM	7	CD1	LEU	A	8	-33.277	-6.303	6.927
ATOM	8	CD2	LEU	A	8	-31.065	-7.215	7.433
ATOM	0	N	GLU	A	9	-34.172	-11.639	7.928
ATOM	1	CA	GLU	A	9	-34.773	-12.724	8.726
ATOM	2	C	GLU	A	9	-33.859	-13.911	8.810
ATOM	3	O	GLU	A	9	-33.534	-14.473	9.853
ATOM	4	H	GLU	A	9	-34.615	-11.320	7.118
ATOM	5	CB	GLU	A	9	-36.097	-13.157	8.070
ATOM	6	CG	GLU	A	9	-37.283	-12.374	8.610
ATOM	7	CD	GLU	A	9	-38.427	-12.313	7.611
ATOM	8	OE1	GLU	A	9	-38.187	-12.234	6.397
ATOM	9	OE2	GLU	A	9	-39.600	-12.342	8.010
ATOM	0	N	ALA	A	10	-33.346	-14.271	7.656
ATOM	1	CA	ALA	A	10	-32.475	-15.418	7.633
ATOM	2	C	ALA	A	10	-31.251	-15.339	8.478
ATOM	3	O	ALA	A	10	-30.805	-16.210	9.203
ATOM	4	H	ALA	A	10	-33.548	-13.776	6.838
ATOM	5	CB	ALA	A	10	-32.024	-15.636	6.176
ATOM	0	N	CYS	A	11	-30.626	-14.163	8.408
ATOM	1	CA	CYS	A	11	-29.466	-13.888	9.224
ATOM	2	C	CYS	A	11	-29.797	-13.997	10.681
ATOM	3	O	CYS	A	11	-29.166	-14.669	11.494
ATOM	4	H	CYS	A	11	-30.958	-13.476	7.797
ATOM	5	CB	CYS	A	11	-28.966	-12.462	8.927
ATOM	6	SG	CYS	A	11	-28.279	-12.219	7.259
ATOM	0	N	ALA	A	12	-30.894	-13.365	11.028
ATOM	1	CA	ALA	A	12	-31.277	-13.388	12.416
ATOM	2	C	ALA	A	12	-31.548	-14.776	12.926
ATOM	3	O	ALA	A	12	-31.143	-15.153	14.014
ATOM	4	H	ALA	A	12	-31.436	-12.894	10.365
ATOM	5	CB	ALA	A	12	-32.554	-12.545	12.590
ATOM	0	N	PHE	A	13	-32.184	-15.594	12.107
ATOM	1	CA	PHE	A	13	-32.404	-16.994	12.516
ATOM	2	C	PHE	A	13	-31.118	-17.814	12.671
ATOM	3	O	PHE	A	13	-30.978	-18.575	13.632
ATOM	4	H	PHE	A	13	-32.505	-15.277	11.240
ATOM	5	CB	PHE	A	13	-33.284	-17.676	11.452

ATOM 6 CG PHE A 13 -33.514 -19.147 11.761
ATOM 7 CD1 PHE A 13 -34.422 -19.511 12.720
ATOM 8 CE1 PHE A 13 -34.630 -20.837 12.998
ATOM 9 CZ PHE A 13 -33.928 -21.797 12.318
ATOM 10 CE2 PHE A 13 -33.020 -21.433 11.360
ATOM 11 CD2 PHE A 13 -32.813 -20.107 11.081
ATOM 0 N LEU A 14 -30.179 -17.658 11.740
ATOM 1 CA LEU A 14 -28.937 -18.422 11.820
ATOM 2 C LEU A 14 -28.203 -17.895 13.064
ATOM 3 O LEU A 14 -27.639 -18.731 13.752
ATOM 4 H LEU A 14 -30.319 -17.032 11.003
ATOM 5 CB LEU A 14 -28.084 -18.195 10.559
ATOM 6 CG LEU A 14 -28.771 -18.711 9.305
ATOM 7 CD1 LEU A 14 -28.051 -18.275 8.040
ATOM 8 CD2 LEU A 14 -28.843 -20.229 9.279
ATOM 0 N ARG A 15 -28.222 -16.620 13.408
ATOM 1 CA ARG A 15 -27.552 -16.200 14.652
ATOM 2 C ARG A 15 -28.141 -16.928 15.875
ATOM 3 O ARG A 15 -27.429 -17.339 16.799
ATOM 4 H ARG A 15 -28.676 -15.962 12.845
ATOM 5 CB ARG A 15 -27.736 -14.682 14.836
ATOM 6 CG ARG A 15 -26.973 -14.157 16.042
ATOM 7 CD ARG A 15 -26.657 -12.676 15.915
ATOM 8 NE ARG A 15 -25.819 -12.195 17.023
ATOM 9 CZ ARG A 15 -25.441 -10.900 17.071
ATOM 10 NH1 ARG A 15 -24.677 -10.462 18.081
ATOM 11 NH2 ARG A 15 -25.830 -10.053 16.108
TER
ENDMDL
MODEL 4 **RCSSSPLLEACAFLR**
ATOM 0 N ARG A 1 -41.049 -11.104 14.527
ATOM 1 CA ARG A 1 -40.397 -9.808 14.393
ATOM 2 C ARG A 1 -39.027 -9.928 13.737
ATOM 3 O ARG A 1 -38.323 -8.931 13.578
ATOM 4 H ARG A 1 -41.974 -11.169 14.950
ATOM 5 CB ARG A 1 -40.226 -9.187 15.792
ATOM 6 CG ARG A 1 -39.662 -7.778 15.725
ATOM 7 CD ARG A 1 -40.021 -6.961 16.955
ATOM 8 NE ARG A 1 -39.577 -5.565 16.838
ATOM 9 CZ ARG A 1 -39.814 -4.695 17.843
ATOM 10 NH1 ARG A 1 -39.409 -3.422 17.737
ATOM 11 NH2 ARG A 1 -40.453 -5.104 18.947
ATOM 0 N CYS A 2 -38.642 -11.145 13.364

```
ATOM      1  CA  CYS A   2     -37.288 -11.380  12.851
ATOM      2  C   CYS A   2     -37.231 -11.549  11.347
ATOM      3  O   CYS A   2     -36.168 -11.830  10.800
ATOM      4  H   CYS A   2     -39.268 -11.892  13.432
ATOM      5  CB  CYS A   2     -36.734 -12.660  13.504
ATOM      6  SG  CYS A   2     -36.534 -12.577  15.311
ATOM      0  N   SER A   3     -38.362 -11.372  10.676
ATOM      1  CA  SER A   3     -38.458 -11.699   9.252
ATOM      2  C   SER A   3     -38.226 -10.518   8.312
ATOM      3  O   SER A   3     -38.309 -10.671   7.087
ATOM      4  H   SER A   3     -39.145 -11.016  11.140
ATOM      5  CB  SER A   3     -39.867 -12.259   8.979
ATOM      6  OG  SER A   3     -40.808 -11.199   9.172
ATOM      0  N   SER A   4     -37.972  -9.337   8.868
ATOM      1  CA  SER A   4     -37.916  -8.130   8.043
ATOM      2  C   SER A   4     -37.107  -7.806   6.591
ATOM      3  O   SER A   4     -37.231  -8.292   5.468
ATOM      4  H   SER A   4     -37.821  -9.274   9.831
ATOM      5  CB  SER A   4     -37.386  -7.051   9.005
ATOM      6  OG  SER A   4     -35.988  -7.280   9.199
ATOM      0  N   SER A   5     -36.127  -6.991   6.952
ATOM      1  CA  SER A   5     -35.092  -6.602   6.010
ATOM      2  C   SER A   5     -34.016  -7.560   5.726
ATOM      3  O   SER A   5     -34.068  -8.689   6.203
ATOM      4  H   SER A   5     -36.098  -6.646   7.866
ATOM      5  CB  SER A   5     -34.432  -5.323   6.557
ATOM      6  OG  SER A   5     -34.130  -5.538   7.938
ATOM      0  N   PRO A   6     -33.003  -7.149   4.994
ATOM      1  CA  PRO A   6     -31.881  -8.019   4.708
ATOM      2  C   PRO A   6     -31.285  -8.583   6.031
ATOM      3  O   PRO A   6     -31.065  -9.781   6.202
ATOM      4  CB  PRO A   6     -30.839  -7.164   3.963
ATOM      5  CG  PRO A   6     -31.661  -5.994   3.391
ATOM      6  CD  PRO A   6     -32.778  -5.810   4.421
ATOM      0  N   LEU A   7     -30.960  -7.662   6.936
ATOM      1  CA  LEU A   7     -30.300  -8.040   8.171
ATOM      2  C   LEU A   7     -31.255  -8.914   9.032
ATOM      3  O   LEU A   7     -30.772  -9.892   9.608
ATOM      4  H   LEU A   7     -31.168  -6.722   6.768
ATOM      5  CB  LEU A   7     -29.921  -6.772   8.959
ATOM      6  CG  LEU A   7     -28.942  -5.898   8.193
ATOM      7  CD1 LEU A   7     -28.754  -4.541   8.852
ATOM      8  CD2 LEU A   7     -27.567  -6.539   8.088
```

ATOM	0	N	LEU	A	8	-32.546	-8.598	9.120
ATOM	1	CA	LEU	A	8	-33.441	-9.407	9.942
ATOM	2	C	LEU	A	8	-33.546	-10.795	9.346
ATOM	3	O	LEU	A	8	-33.562	-11.786	10.053
ATOM	4	H	LEU	A	8	-32.895	-7.826	8.634
ATOM	5	CB	LEU	A	8	-34.836	-8.757	9.979
ATOM	6	CG	LEU	A	8	-34.805	-7.379	10.620
ATOM	7	CD1	LEU	A	8	-36.171	-6.712	10.602
ATOM	8	CD2	LEU	A	8	-34.354	-7.436	12.070
ATOM	0	N	GLU	A	9	-33.628	-10.936	8.022
ATOM	1	CA	GLU	A	9	-33.674	-12.246	7.415
ATOM	2	C	GLU	A	9	-32.469	-13.084	7.662
ATOM	3	O	GLU	A	9	-32.489	-14.265	7.957
ATOM	4	H	GLU	A	9	-33.656	-10.142	7.453
ATOM	5	CB	GLU	A	9	-33.831	-12.069	5.893
ATOM	6	CG	GLU	A	9	-35.267	-11.764	5.500
ATOM	7	CD	GLU	A	9	-35.370	-11.244	4.075
ATOM	8	OE1	GLU	A	9	-34.395	-10.691	3.546
ATOM	9	OE2	GLU	A	9	-36.428	-11.374	3.443
ATOM	0	N	ALA	A	10	-31.296	-12.461	7.539
ATOM	1	CA	ALA	A	10	-30.055	-13.151	7.807
ATOM	2	C	ALA	A	10	-29.972	-13.566	9.252
ATOM	3	O	ALA	A	10	-29.552	-14.676	9.542
ATOM	4	H	ALA	A	10	-31.274	-11.523	7.264
ATOM	5	CB	ALA	A	10	-28.876	-12.215	7.484
ATOM	0	N	CYS	A	11	-30.406	-12.731	10.178
ATOM	1	CA	CYS	A	11	-30.388	-13.151	11.592
ATOM	2	C	CYS	A	11	-31.248	-14.328	11.908
ATOM	3	O	CYS	A	11	-30.923	-15.257	12.623
ATOM	4	H	CYS	A	11	-30.736	-11.845	9.931
ATOM	5	CB	CYS	A	11	-30.867	-11.966	12.451
ATOM	6	SG	CYS	A	11	-29.674	-10.600	12.610
ATOM	0	N	ALA	A	12	-32.458	-14.327	11.346
ATOM	1	CA	ALA	A	12	-33.362	-15.439	11.526
ATOM	2	C	ALA	A	12	-32.859	-16.750	10.917
ATOM	3	O	ALA	A	12	-32.969	-17.808	11.541
ATOM	4	H	ALA	A	12	-32.737	-13.563	10.804
ATOM	5	CB	ALA	A	12	-34.708	-15.084	10.867
ATOM	0	N	PHE	A	13	-32.306	-16.689	9.706
ATOM	1	CA	PHE	A	13	-31.827	-17.908	9.060
ATOM	2	C	PHE	A	13	-30.568	-18.298	9.921
ATOM	3	O	PHE	A	13	-30.472	-19.487	10.222
ATOM	4	H	PHE	A	13	-32.220	-15.829	9.250

ATOM 5 CB PHE A 13 -31.433 -17.640 7.596
ATOM 6 CG PHE A 13 -30.874 -18.882 6.921
ATOM 7 CD1 PHE A 13 -31.721 -19.869 6.492
ATOM 8 CE1 PHE A 13 -31.217 -20.988 5.884
ATOM 9 CZ PHE A 13 -29.866 -21.120 5.704
ATOM 10 CE2 PHE A 13 -29.018 -20.133 6.132
ATOM 11 CD2 PHE A 13 -29.522 -19.014 6.741
ATOM 0 N LEU A 14 -29.688 -17.365 10.255
ATOM 1 CA LEU A 14 -28.505 -17.697 11.030
ATOM 2 C LEU A 14 -28.832 -18.328 12.394
ATOM 3 O LEU A 14 -28.210 -19.322 12.776
ATOM 4 H LEU A 14 -29.837 -16.440 9.976
ATOM 5 CB LEU A 14 -27.697 -16.407 11.269
ATOM 6 CG LEU A 14 -27.250 -15.765 9.966
ATOM 7 CD1 LEU A 14 -26.670 -14.378 10.185
ATOM 8 CD2 LEU A 14 -26.186 -16.590 9.260
ATOM 0 N ARG A 15 -29.802 -17.771 13.118
ATOM 1 CA ARG A 15 -30.145 -18.322 14.426
ATOM 2 C ARG A 15 -30.933 -19.625 14.310
ATOM 3 O ARG A 15 -31.290 -20.225 15.328
ATOM 4 H ARG A 15 -30.285 -16.994 12.774
ATOM 5 CB ARG A 15 -30.998 -17.293 15.191
ATOM 6 CG ARG A 15 -31.293 -17.743 16.613
ATOM 7 CD ARG A 15 -31.595 -16.569 17.531
ATOM 8 NE ARG A 15 -31.764 -16.992 18.928
ATOM 9 CZ ARG A 15 -32.037 -16.077 19.882
ATOM 10 NH1 ARG A 15 -32.191 -16.463 21.156
ATOM 11 NH2 ARG A 15 -32.155 -14.783 19.555
TER
ENDMDL
MODEL 5 **RCSSSPLLEACAFLR**
ATOM 0 N ARG A 1 -36.434 -8.941 15.808
ATOM 1 CA ARG A 1 -35.311 -9.699 15.303
ATOM 2 C ARG A 1 -35.621 -10.205 13.908
ATOM 3 O ARG A 1 -34.708 -10.373 13.130
ATOM 4 H ARG A 1 -36.392 -8.526 16.739
ATOM 5 CB ARG A 1 -35.042 -10.897 16.233
ATOM 6 CG ARG A 1 -34.541 -10.457 17.599
ATOM 7 CD ARG A 1 -34.217 -11.641 18.495
ATOM 8 NE ARG A 1 -33.789 -11.216 19.835
ATOM 9 CZ ARG A 1 -33.510 -12.140 20.779
ATOM 10 NH1 ARG A 1 -33.120 -11.753 22.001
ATOM 11 NH2 ARG A 1 -33.624 -13.445 20.494

ATOM	0	N	CYS	A	2	-36.891	-10.531	13.659
ATOM	1	CA	CYS	A	2	-37.372	-11.016	12.360
ATOM	2	C	CYS	A	2	-38.108	-9.918	11.583
ATOM	3	O	CYS	A	2	-38.950	-10.202	10.798
ATOM	4	H	CYS	A	2	-37.540	-10.442	14.384
ATOM	5	CB	CYS	A	2	-38.337	-12.194	12.591
ATOM	6	SG	CYS	A	2	-37.584	-13.667	13.349
ATOM	0	N	SER	A	3	-37.797	-8.656	11.815
ATOM	1	CA	SER	A	3	-38.444	-7.572	11.079
ATOM	2	C	SER	A	3	-38.355	-7.769	9.559
ATOM	3	O	SER	A	3	-37.334	-8.206	9.020
ATOM	4	H	SER	A	3	-37.123	-8.442	12.489
ATOM	5	CB	SER	A	3	-37.758	-6.243	11.446
ATOM	6	OG	SER	A	3	-36.417	-6.287	10.953
ATOM	0	N	SER	A	4	-39.443	-7.460	8.869
ATOM	1	CA	SER	A	4	-39.505	-7.728	7.439
ATOM	2	C	SER	A	4	-38.503	-7.586	6.320
ATOM	3	O	SER	A	4	-38.737	-8.181	5.267
ATOM	4	H	SER	A	4	-40.207	-7.050	9.320
ATOM	5	CB	SER	A	4	-40.688	-6.863	6.966
ATOM	6	OG	SER	A	4	-40.252	-5.502	6.927
ATOM	0	N	SER	A	5	-37.287	-7.183	6.673
ATOM	1	CA	SER	A	5	-36.111	-7.408	5.832
ATOM	2	C	SER	A	5	-35.662	-8.711	5.464
ATOM	3	O	SER	A	5	-35.524	-9.413	6.473
ATOM	4	H	SER	A	5	-37.173	-6.719	7.526
ATOM	5	CB	SER	A	5	-34.929	-6.744	6.564
ATOM	6	OG	SER	A	5	-34.630	-7.527	7.722
ATOM	0	N	PRO	A	6	-35.419	-9.168	4.239
ATOM	1	CA	PRO	A	6	-34.956	-10.540	4.015
ATOM	2	C	PRO	A	6	-33.558	-10.886	4.603
ATOM	3	O	PRO	A	6	-33.433	-11.994	5.136
ATOM	4	CB	PRO	A	6	-34.925	-10.711	2.485
ATOM	5	CG	PRO	A	6	-35.835	-9.582	1.967
ATOM	6	CD	PRO	A	6	-35.647	-8.473	3.006
ATOM	0	N	LEU	A	7	-32.565	-10.016	4.531
ATOM	1	CA	LEU	A	7	-31.213	-10.364	4.959
ATOM	2	C	LEU	A	7	-31.373	-10.325	6.413
ATOM	3	O	LEU	A	7	-30.714	-11.186	7.002
ATOM	4	H	LEU	A	7	-32.739	-9.119	4.183
ATOM	5	CB	LEU	A	7	-30.179	-9.331	4.474
ATOM	6	CG	LEU	A	7	-30.042	-9.329	2.961
ATOM	7	CD1	LEU	A	7	-29.212	-8.156	2.465

ATOM	8	CD2	LEU	A	7	-29.377	-10.596	2.448
ATOM	0	N	LEU	A	8	-32.151	-9.457	7.057
ATOM	1	CA	LEU	A	8	-32.227	-9.485	8.514
ATOM	2	C	LEU	A	8	-32.935	-10.805	8.949
ATOM	3	O	LEU	A	8	-32.473	-11.381	9.936
ATOM	4	H	LEU	A	8	-32.672	-8.800	6.554
ATOM	5	CB	LEU	A	8	-33.035	-8.274	9.017
ATOM	6	CG	LEU	A	8	-32.369	-6.956	8.657
ATOM	7	CD1	LEU	A	8	-33.275	-5.767	8.933
ATOM	8	CD2	LEU	A	8	-31.086	-6.734	9.442
ATOM	0	N	GLU	A	9	-33.984	-11.273	8.274
ATOM	1	CA	GLU	A	9	-34.646	-12.498	8.712
ATOM	2	C	GLU	A	9	-33.628	-13.627	8.485
ATOM	3	O	GLU	A	9	-33.572	-14.486	9.351
ATOM	4	H	GLU	A	9	-34.310	-10.797	7.485
ATOM	5	CB	GLU	A	9	-35.918	-12.748	7.881
ATOM	6	CG	GLU	A	9	-36.994	-11.711	8.158
ATOM	7	CD	GLU	A	9	-38.230	-11.930	7.301
ATOM	8	OE1	GLU	A	9	-39.242	-11.237	7.484
ATOM	9	OE2	GLU	A	9	-38.227	-12.801	6.419
ATOM	0	N	ALA	A	10	-32.816	-13.646	7.443
ATOM	1	CA	ALA	A	10	-31.819	-14.726	7.328
ATOM	2	C	ALA	A	10	-30.871	-14.722	8.491
ATOM	3	O	ALA	A	10	-30.604	-15.703	9.181
ATOM	4	H	ALA	A	10	-32.875	-12.954	6.757
ATOM	5	CB	ALA	A	10	-31.016	-14.532	6.028
ATOM	0	N	CYS	A	11	-30.392	-13.535	8.784
ATOM	1	CA	CYS	A	11	-29.453	-13.434	9.872
ATOM	2	C	CYS	A	11	-30.035	-13.864	11.189
ATOM	3	O	CYS	A	11	-29.412	-14.566	11.970
ATOM	4	H	CYS	A	11	-30.665	-12.743	8.281
ATOM	5	CB	CYS	A	11	-28.995	-11.969	9.994
ATOM	6	SG	CYS	A	11	-27.866	-11.405	8.683
ATOM	0	N	ALA	A	12	-31.284	-13.508	11.430
ATOM	1	CA	ALA	A	12	-31.939	-13.972	12.667
ATOM	2	C	ALA	A	12	-32.090	-15.472	12.680
ATOM	3	O	ALA	A	12	-31.858	-16.099	13.701
ATOM	4	H	ALA	A	12	-31.769	-12.943	10.797
ATOM	5	CB	ALA	A	12	-33.335	-13.328	12.768
ATOM	0	N	PHE	A	13	-32.420	-16.084	11.557
ATOM	1	CA	PHE	A	13	-32.504	-17.556	11.537
ATOM	2	C	PHE	A	13	-31.157	-18.186	11.791
ATOM	3	O	PHE	A	13	-31.068	-19.154	12.531

```
ATOM     4  H   PHE A  13     -32.606 -15.569  10.748
ATOM     5  CB  PHE A  13     -33.016 -18.012  10.158
ATOM     6  CG  PHE A  13     -33.070 -19.527  10.044
ATOM     7  CD1 PHE A  13     -34.093 -20.223  10.633
ATOM     8  CE1 PHE A  13     -34.141 -21.588  10.530
ATOM     9  CZ  PHE A  13     -33.168 -22.257   9.837
ATOM    10  CE2 PHE A  13     -32.145 -21.562   9.247
ATOM    11  CD2 PHE A  13     -32.097 -20.197   9.350
ATOM     0  N   LEU A  14     -30.088 -17.636  11.246
ATOM     1  CA  LEU A  14     -28.758 -18.199  11.542
ATOM     2  C   LEU A  14     -28.411 -18.052  13.001
ATOM     3  O   LEU A  14     -27.881 -18.976  13.598
ATOM     4  H   LEU A  14     -30.176 -16.864  10.653
ATOM     5  CB  LEU A  14     -27.700 -17.462  10.699
ATOM     6  CG  LEU A  14     -27.943 -17.627   9.208
ATOM     7  CD1 LEU A  14     -27.057 -16.710   8.381
ATOM     8  CD2 LEU A  14     -27.675 -19.049   8.742
ATOM     0  N   ARG A  15     -28.743 -16.933  13.621
ATOM     1  CA  ARG A  15     -28.472 -16.802  15.065
ATOM     2  C   ARG A  15     -29.189 -17.904  15.867
ATOM     3  O   ARG A  15     -28.651 -18.472  16.825
ATOM     4  H   ARG A  15     -29.163 -16.201  13.129
ATOM     5  CB  ARG A  15     -28.969 -15.427  15.547
ATOM     6  CG  ARG A  15     -28.620 -15.175  17.005
ATOM     7  CD  ARG A  15     -28.559 -13.691  17.328
ATOM     8  NE  ARG A  15     -28.108 -13.445  18.705
ATOM     9  CZ  ARG A  15     -27.995 -12.180  19.163
ATOM    10  NH1 ARG A  15     -27.583 -11.956  20.418
ATOM    11  NH2 ARG A  15     -28.295 -11.149  18.362
TER
ENDMDL
```

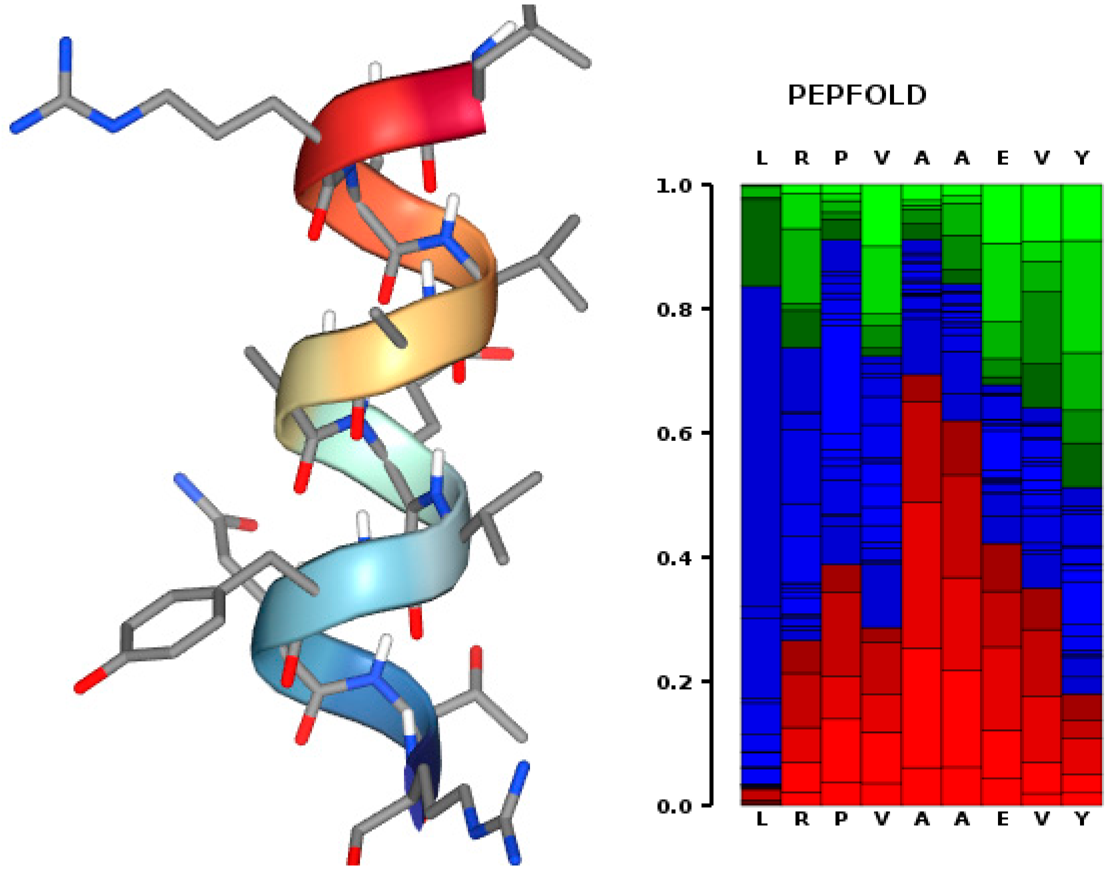

Figure S5 3D structure of LRPVAAEVYQTR and Predicted Local Structure Profile job execution pep-fold4.py --maxSimSze 50 --maxSeqSze 50 --noMQA --sortKey sOPEP -s iSeq.fasta -l PEPFOLD --generator fbt --nRuns 100 --mcSteps 30000 --mcT 370 --seed 1 Debye-Huckel formalism Solvent pH7.5 Ionic strength (150mM)

Table S6 LRPVAAEVYQTR PEPFOLD - 5 bestmodels.pdb

MODEL 1

ATOM	0	N	LEU	A	1	119.852	51.677	228.189
ATOM	1	CA	LEU	A	1	119.205	52.734	228.953
ATOM	2	C	LEU	A	1	120.018	54.023	228.864
ATOM	3	O	LEU	A	1	119.468	55.103	228.650
ATOM	4	H	LEU	A	1	119.433	50.748	228.148
ATOM	5	CB	LEU	A	1	119.097	52.304	230.428
ATOM	6	CG	LEU	A	1	118.247	51.055	230.596
ATOM	7	CD1	LEU	A	1	118.336	50.490	232.004
ATOM	8	CD2	LEU	A	1	116.778	51.324	230.314
ATOM	0	N	ARG	A	2	121.330	53.911	229.032
ATOM	1	CA	ARG	A	2	122.186	55.091	228.954
ATOM	2	C	ARG	A	2	122.064	55.727	227.575
ATOM	3	O	ARG	A	2	121.930	56.948	227.453
ATOM	4	H	ARG	A	2	121.726	53.035	229.207
ATOM	5	CB	ARG	A	2	123.650	54.680	229.199
ATOM	6	CG	ARG	A	2	124.581	55.881	229.229

ATOM	7	CD	ARG	A	2	125.850	55.598	230.016
ATOM	8	NE	ARG	A	2	126.696	56.793	230.144
ATOM	9	CZ	ARG	A	2	127.866	56.724	230.815
ATOM	10	NH1	ARG	A	2	128.637	57.813	230.932
ATOM	11	NH2	ARG	A	2	128.256	55.565	231.363
ATOM	0	N	PRO	A	3	122.110	54.897	226.534
ATOM	1	CA	PRO	A	3	122.009	55.387	225.166
ATOM	2	C	PRO	A	3	120.674	56.080	224.880
ATOM	3	O	PRO	A	3	120.644	57.149	224.265
ATOM	4	CB	PRO	A	3	122.168	54.152	224.260
ATOM	5	CG	PRO	A	3	122.865	53.117	225.164
ATOM	6	CD	PRO	A	3	122.343	53.462	226.561
ATOM	0	N	VAL	A	4	119.570	55.482	225.327
ATOM	1	CA	VAL	A	4	118.262	56.078	225.072
ATOM	2	C	VAL	A	4	118.267	57.421	225.798
ATOM	3	O	VAL	A	4	117.898	58.448	225.234
ATOM	4	H	VAL	A	4	119.636	54.644	225.826
ATOM	5	CB	VAL	A	4	117.144	55.175	225.625
ATOM	6	CG1	VAL	A	4	115.785	55.855	225.572
ATOM	7	CG2	VAL	A	4	117.024	53.875	224.847
ATOM	0	N	ALA	A	5	118.746	57.451	227.020
ATOM	1	CA	ALA	A	5	118.722	58.708	227.724
ATOM	2	C	ALA	A	5	119.538	59.773	227.047
ATOM	3	O	ALA	A	5	119.131	60.918	226.928
ATOM	4	H	ALA	A	5	119.108	56.647	227.440
ATOM	5	CB	ALA	A	5	119.281	58.490	229.142
ATOM	0	N	ALA	A	6	120.689	59.394	226.521
ATOM	1	CA	ALA	A	6	121.495	60.374	225.770
ATOM	2	C	ALA	A	6	120.744	60.907	224.585
ATOM	3	O	ALA	A	6	120.599	62.099	224.326
ATOM	4	H	ALA	A	6	121.003	58.475	226.628
ATOM	5	CB	ALA	A	6	122.785	59.693	225.279
ATOM	0	N	GLU	A	7	120.160	59.978	223.863
ATOM	1	CA	GLU	A	7	119.435	60.392	222.689
ATOM	2	C	GLU	A	7	118.284	61.305	223.003
ATOM	3	O	GLU	A	7	118.051	62.301	222.335
ATOM	4	H	GLU	A	7	120.214	59.035	224.115
ATOM	5	CB	GLU	A	7	118.886	59.139	221.982
ATOM	6	CG	GLU	A	7	119.998	58.282	221.397
ATOM	7	CD	GLU	A	7	119.461	57.015	220.752
ATOM	8	OE1	GLU	A	7	118.286	56.670	220.947
ATOM	9	OE2	GLU	A	7	120.198	56.326	220.031
ATOM	0	N	VAL	A	8	117.579	61.021	224.083

```
ATOM      1  CA  VAL A   8      116.490  61.928 224.492
ATOM      2  C   VAL A   8      117.007  63.306 224.784
ATOM      3  O   VAL A   8      116.547  64.342 224.309
ATOM      4  H   VAL A   8      117.775  60.219 224.606
ATOM      5  CB  VAL A   8      115.819  61.367 225.760
ATOM      6  CG1 VAL A   8      114.786  62.327 226.328
ATOM      7  CG2 VAL A   8      115.108  60.051 225.493
ATOM      0  N   TYR A   9      118.078  63.324 225.543
ATOM      1  CA  TYR A   9      118.633  64.605 225.898
ATOM      2  C   TYR A   9      119.090  65.396 224.704
ATOM      3  O   TYR A   9      118.861  66.590 224.597
ATOM      4  H   TYR A   9      118.487  62.494 225.858
ATOM      5  CB  TYR A   9      119.841  64.379 226.827
ATOM      6  CG  TYR A   9      120.524  65.685 227.200
ATOM      7  CD1 TYR A   9      119.969  66.501 228.150
ATOM      8  CE1 TYR A   9      120.585  67.677 228.487
ATOM      9  CZ  TYR A   9      121.756  68.038 227.873
ATOM     10  CE2 TYR A   9      122.311  67.221 226.923
ATOM     11  CD2 TYR A   9      121.695  66.045 226.586
ATOM     12  OH  TYR A   9      122.376  69.223 228.212
ATOM      0  N   GLN A  10      119.689  64.721 223.740
ATOM      1  CA  GLN A  10      120.084  65.425 222.506
ATOM      2  C   GLN A  10      118.907  65.962 221.684
ATOM      3  O   GLN A  10      118.957  67.090 221.187
ATOM      4  H   GLN A  10      119.868  63.765 223.842
ATOM      5  CB  GLN A  10      120.881  64.446 221.623
ATOM      6  CG  GLN A  10      122.145  63.958 222.312
ATOM      7  CD  GLN A  10      122.906  62.955 221.461
ATOM      8  OE1 GLN A  10      122.443  62.589 220.371
ATOM      9  NE2 GLN A  10      124.078  62.466 221.889
ATOM      0  N   THR A  11      117.847  65.167 221.543
ATOM      1  CA  THR A  11      116.699  65.611 220.758
ATOM      2  C   THR A  11      116.012  66.793 221.508
ATOM      3  O   THR A  11      115.611  67.731 220.815
ATOM      4  H   THR A  11      117.837  64.286 221.967
ATOM      5  CB  THR A  11      115.700  64.451 220.594
ATOM      6  OG1 THR A  11      116.318  63.369 219.892
ATOM      7  CG2 THR A  11      114.464  64.869 219.815
ATOM      0  N   ARG A  12      115.873  66.774 222.833
ATOM      1  CA  ARG A  12      115.205  67.880 223.513
ATOM      2  C   ARG A  12      116.088  69.124 223.589
ATOM      3  O   ARG A  12      115.664  70.152 224.123
ATOM      4  H   ARG A  12      116.220  66.021 223.351
```

```
ATOM      5  CB  ARG A  12      114.846  67.440 224.945
ATOM      6  CG  ARG A  12      114.036  68.496 225.678
ATOM      7  CD  ARG A  12      113.201  67.897 226.798
ATOM      8  NE  ARG A  12      112.329  68.898 227.431
ATOM      9  CZ  ARG A  12      111.516  68.539 228.447
ATOM     10  NH1 ARG A  12      110.722  69.451 229.024
ATOM     11  NH2 ARG A  12      111.504  67.270 228.879
TER
ENDMDL
MODEL        2
ATOM      0  N   LEU A   1      119.842  51.680 228.215
ATOM      1  CA  LEU A   1      119.166  52.735 228.954
ATOM      2  C   LEU A   1      119.971  54.029 228.881
ATOM      3  O   LEU A   1      119.419  55.104 228.646
ATOM      4  H   LEU A   1      119.431  50.747 228.166
ATOM      5  CB  LEU A   1      119.018  52.312 230.427
ATOM      6  CG  LEU A   1      118.173  51.057 230.577
ATOM      7  CD1 LEU A   1      118.226  50.501 231.991
ATOM      8  CD2 LEU A   1      116.711  51.314 230.252
ATOM      0  N   ARG A   2      121.279  53.929 229.087
ATOM      1  CA  ARG A   2      122.128  55.115 229.027
ATOM      2  C   ARG A   2      122.041  55.742 227.641
ATOM      3  O   ARG A   2      121.901  56.962 227.510
ATOM      4  H   ARG A   2      121.676  53.057 229.278
ATOM      5  CB  ARG A   2      123.587  54.716 229.316
ATOM      6  CG  ARG A   2      124.508  55.924 229.366
ATOM      7  CD  ARG A   2      125.756  55.655 230.190
ATOM      8  NE  ARG A   2      126.589  56.857 230.337
ATOM      9  CZ  ARG A   2      127.740  56.800 231.041
ATOM     10  NH1 ARG A   2      128.499  57.896 231.175
ATOM     11  NH2 ARG A   2      128.122  55.647 231.607
ATOM      0  N   PRO A   3      122.123  54.908 226.607
ATOM      1  CA  PRO A   3      122.057  55.390 225.234
ATOM      2  C   PRO A   3      120.725  56.071 224.906
ATOM      3  O   PRO A   3      120.705  57.136 224.285
ATOM      4  CB  PRO A   3      122.251  54.151 224.340
ATOM      5  CG  PRO A   3      122.929  53.127 225.269
ATOM      6  CD  PRO A   3      122.365  53.475 226.649
ATOM      0  N   VAL A   4      119.614  55.467 225.325
ATOM      1  CA  VAL A   4      118.310  56.052 225.030
ATOM      2  C   VAL A   4      118.283  57.398 225.748
ATOM      3  O   VAL A   4      117.923  58.420 225.168
ATOM      4  H   VAL A   4      119.671  54.633 225.830
```

ATOM	5	CB	VAL	A	4	117.183	55.144	225.555
ATOM	6	CG1	VAL	A	4	115.821	55.813	225.460
ATOM	7	CG2	VAL	A	4	117.095	53.839	224.780
ATOM	0	N	ALA	A	5	118.727	57.439	226.983
ATOM	1	CA	ALA	A	5	118.673	58.699	227.679
ATOM	2	C	ALA	A	5	119.500	59.767	227.020
ATOM	3	O	ALA	A	5	119.089	60.908	226.884
ATOM	4	H	ALA	A	5	119.083	56.639	227.417
ATOM	5	CB	ALA	A	5	119.193	58.492	229.114
ATOM	0	N	ALA	A	6	120.669	59.394	226.529
ATOM	1	CA	ALA	A	6	121.489	60.376	225.797
ATOM	2	C	ALA	A	6	120.769	60.897	224.588
ATOM	3	O	ALA	A	6	120.622	62.086	224.318
ATOM	4	H	ALA	A	6	120.987	58.477	226.650
ATOM	5	CB	ALA	A	6	122.798	59.702	225.346
ATOM	0	N	GLU	A	7	120.212	59.960	223.854
ATOM	1	CA	GLU	A	7	119.518	60.362	222.658
ATOM	2	C	GLU	A	7	118.352	61.269	222.934
ATOM	3	O	GLU	A	7	118.131	62.259	222.254
ATOM	4	H	GLU	A	7	120.266	59.019	224.112
ATOM	5	CB	GLU	A	7	118.999	59.101	221.942
ATOM	6	CG	GLU	A	7	120.133	58.250	221.394
ATOM	7	CD	GLU	A	7	119.624	56.976	220.741
ATOM	8	OE1	GLU	A	7	118.447	56.623	220.904
ATOM	9	OE2	GLU	A	7	120.387	56.289	220.045
ATOM	0	N	VAL	A	8	117.618	60.984	223.995
ATOM	1	CA	VAL	A	8	116.512	61.885	224.368
ATOM	2	C	VAL	A	8	117.022	63.248	224.762
ATOM	3	O	VAL	A	8	116.442	64.251	224.377
ATOM	4	H	VAL	A	8	117.806	60.187	224.527
ATOM	5	CB	VAL	A	8	115.746	61.275	225.556
ATOM	6	CG1	VAL	A	8	114.684	62.220	226.094
ATOM	7	CG2	VAL	A	8	115.043	59.982	225.177
ATOM	0	N	TYR	A	9	118.130	63.327	225.477
ATOM	1	CA	TYR	A	9	118.679	64.654	225.811
ATOM	2	C	TYR	A	9	119.115	65.396	224.573
ATOM	3	O	TYR	A	9	118.865	66.585	224.456
ATOM	4	H	TYR	A	9	118.582	62.515	225.781
ATOM	5	CB	TYR	A	9	119.894	64.476	226.741
ATOM	6	CG	TYR	A	9	120.551	65.804	227.080
ATOM	7	CD1	TYR	A	9	119.982	66.631	228.012
ATOM	8	CE1	TYR	A	9	120.574	67.828	228.317
ATOM	9	CZ	TYR	A	9	121.735	68.198	227.690

```
ATOM     10  CE2 TYR A   9      122.304  67.371 226.758
ATOM     11  CD2 TYR A   9      121.712  66.173 226.453
ATOM     12  OH  TYR A   9      122.332  69.404 227.998
ATOM      0  N   GLN A  10      119.715  64.721 223.609
ATOM      1  CA  GLN A  10      120.084  65.419 222.364
ATOM      2  C   GLN A  10      118.864  65.903 221.622
ATOM      3  O   GLN A  10      118.859  67.015 221.118
ATOM      4  H   GLN A  10      119.911  63.770 223.718
ATOM      5  CB  GLN A  10      120.868  64.451 221.458
ATOM      6  CG  GLN A  10      122.167  63.994 222.102
ATOM      7  CD  GLN A  10      122.917  63.001 221.229
ATOM      8  OE1 GLN A  10      122.421  62.618 220.160
ATOM      9  NE2 GLN A  10      124.114  62.539 221.616
ATOM      0  N   THR A  11      117.797  65.125 221.583
ATOM      1  CA  THR A  11      116.573  65.614 220.921
ATOM      2  C   THR A  11      115.925  66.821 221.609
ATOM      3  O   THR A  11      115.499  67.764 220.939
ATOM      4  H   THR A  11      117.817  64.236 221.988
ATOM      5  CB  THR A  11      115.546  64.467 220.894
ATOM      6  OG1 THR A  11      116.086  63.344 220.192
ATOM      7  CG2 THR A  11      114.255  64.875 220.205
ATOM      0  N   ARG A  12      115.856  66.802 222.939
ATOM      1  CA  ARG A  12      115.236  67.912 223.655
ATOM      2  C   ARG A  12      116.134  69.147 223.685
ATOM      3  O   ARG A  12      115.749  70.179 224.243
ATOM      4  H   ARG A  12      116.221  66.044 223.437
ATOM      5  CB  ARG A  12      114.947  67.474 225.102
ATOM      6  CG  ARG A  12      114.187  68.537 225.878
ATOM      7  CD  ARG A  12      113.406  67.944 227.040
ATOM      8  NE  ARG A  12      112.578  68.952 227.718
ATOM      9  CZ  ARG A  12      111.816  68.600 228.775
ATOM     10  NH1 ARG A  12      111.062  69.518 229.394
ATOM     11  NH2 ARG A  12      111.813  67.331 229.206
TER
ENDMDL
MODEL        3
ATOM      0  N   LEU A   1      119.830  51.705 228.256
ATOM      1  CA  LEU A   1      119.213  52.778 229.022
ATOM      2  C   LEU A   1      120.042  54.054 228.903
ATOM      3  O   LEU A   1      119.503  55.140 228.688
ATOM      4  H   LEU A   1      119.397  50.782 228.233
ATOM      5  CB  LEU A   1      119.128  52.365 230.503
ATOM      6  CG  LEU A   1      118.264  51.130 230.700
```

```
ATOM      7  CD1 LEU A   1      118.373  50.579 232.113
ATOM      8  CD2 LEU A   1      116.793  51.417 230.445
ATOM      0  N   ARG A   2      121.355  53.926 229.046
ATOM      1  CA  ARG A   2      122.226  55.093 228.939
ATOM      2  C   ARG A   2      122.086  55.716 227.555
ATOM      3  O   ARG A   2      121.967  56.938 227.424
ATOM      4  H   ARG A   2      121.742  53.046 229.223
ATOM      5  CB  ARG A   2      123.689  54.664 229.159
ATOM      6  CG  ARG A   2      124.637  55.852 229.158
ATOM      7  CD  ARG A   2      125.918  55.559 229.923
ATOM      8  NE  ARG A   2      126.783  56.743 230.022
ATOM      9  CZ  ARG A   2      127.964  56.664 230.670
ATOM     10  NH1 ARG A   2      128.753  57.743 230.761
ATOM     11  NH2 ARG A   2      128.348  55.505 231.223
ATOM      0  N   PRO A   3      122.100  54.875 226.523
ATOM      1  CA  PRO A   3      121.978  55.353 225.152
ATOM      2  C   PRO A   3      120.647  56.061 224.885
ATOM      3  O   PRO A   3      120.620  57.124 224.260
ATOM      4  CB  PRO A   3      122.102  54.106 224.257
ATOM      5  CG  PRO A   3      122.802  53.071 225.157
ATOM      6  CD  PRO A   3      122.312  53.438 226.561
ATOM      0  N   VAL A   4      119.545  55.484 225.360
ATOM      1  CA  VAL A   4      118.241  56.096 225.125
ATOM      2  C   VAL A   4      118.278  57.445 225.836
ATOM      3  O   VAL A   4      117.913  58.472 225.268
ATOM      4  H   VAL A   4      119.608  54.650 225.866
ATOM      5  CB  VAL A   4      117.121  55.215 225.709
ATOM      6  CG1 VAL A   4      115.771  55.913 225.676
ATOM      7  CG2 VAL A   4      116.967  53.908 224.947
ATOM      0  N   ALA A   5      118.782  57.482 227.048
ATOM      1  CA  ALA A   5      118.789  58.746 227.738
ATOM      2  C   ALA A   5      119.606  59.792 227.035
ATOM      3  O   ALA A   5      119.213  60.942 226.912
ATOM      4  H   ALA A   5      119.141  56.676 227.469
ATOM      5  CB  ALA A   5      119.373  58.534 229.148
ATOM      0  N   ALA A   6      120.741  59.391 226.490
ATOM      1  CA  ALA A   6      121.546  60.352 225.713
ATOM      2  C   ALA A   6      120.779  60.883 224.538
ATOM      3  O   ALA A   6      120.646  62.074 224.268
ATOM      4  H   ALA A   6      121.044  58.469 226.600
ATOM      5  CB  ALA A   6      122.817  59.647 225.204
ATOM      0  N   GLU A   7      120.168  59.956 223.837
ATOM      1  CA  GLU A   7      119.426  60.367 222.673
```

ATOM	2	C	GLU	A	7	118.249	61.309	222.934
ATOM	3	O	GLU	A	7	118.030	62.298	222.230
ATOM	4	H	GLU	A	7	120.214	59.015	224.097
ATOM	5	CB	GLU	A	7	118.875	59.102	221.990
ATOM	6	CG	GLU	A	7	119.983	58.246	221.399
ATOM	7	CD	GLU	A	7	119.444	56.967	220.778
ATOM	8	OE1	GLU	A	7	118.275	56.617	220.995
ATOM	9	OE2	GLU	A	7	120.175	56.274	220.054
ATOM	0	N	VAL	A	8	117.494	61.002	223.987
ATOM	1	CA	VAL	A	8	116.344	61.833	224.329
ATOM	2	C	VAL	A	8	116.910	63.207	224.678
ATOM	3	O	VAL	A	8	116.431	64.231	224.197
ATOM	4	H	VAL	A	8	117.709	60.220	224.532
ATOM	5	CB	VAL	A	8	115.594	61.242	225.538
ATOM	6	CG1	VAL	A	8	114.521	62.185	226.059
ATOM	7	CG2	VAL	A	8	114.909	59.929	225.196
ATOM	0	N	TYR	A	9	117.971	63.250	225.450
ATOM	1	CA	TYR	A	9	118.491	64.545	225.811
ATOM	2	C	TYR	A	9	118.983	65.411	224.650
ATOM	3	O	TYR	A	9	118.719	66.613	224.569
ATOM	4	H	TYR	A	9	118.396	62.431	225.770
ATOM	5	CB	TYR	A	9	119.674	64.329	226.773
ATOM	6	CG	TYR	A	9	120.334	65.641	227.166
ATOM	7	CD1	TYR	A	9	119.745	66.450	228.102
ATOM	8	CE1	TYR	A	9	120.340	67.632	228.456
ATOM	9	CZ	TYR	A	9	121.524	68.005	227.876
ATOM	10	CE2	TYR	A	9	122.113	67.195	226.941
ATOM	11	CD2	TYR	A	9	121.518	66.013	226.586
ATOM	12	OH	TYR	A	9	122.123	69.195	228.233
ATOM	0	N	GLN	A	10	119.694	64.772	223.721
ATOM	1	CA	GLN	A	10	120.213	65.501	222.568
ATOM	2	C	GLN	A	10	118.980	65.922	221.752
ATOM	3	O	GLN	A	10	119.011	67.041	221.264
ATOM	4	H	GLN	A	10	119.867	63.814	223.811
ATOM	5	CB	GLN	A	10	121.129	64.594	221.726
ATOM	6	CG	GLN	A	10	122.378	64.182	222.487
ATOM	7	CD	GLN	A	10	123.259	63.248	221.673
ATOM	8	OE1	GLN	A	10	122.891	62.870	220.551
ATOM	9	NE2	GLN	A	10	124.433	62.833	222.168
ATOM	0	N	THR	A	11	117.919	65.145	221.624
ATOM	1	CA	THR	A	11	116.746	65.644	220.882
ATOM	2	C	THR	A	11	116.050	66.842	221.538
ATOM	3	O	THR	A	11	115.673	67.795	220.852

```
ATOM      4  H   THR A  11      117.910  64.251 222.017
ATOM      5  CB  THR A  11      115.724  64.499 220.765
ATOM      6  OG1 THR A  11      116.314  63.385 220.088
ATOM      7  CG2 THR A  11      114.486  64.917 219.989
ATOM      0  N   ARG A  12      115.884  66.804 222.859
ATOM      1  CA  ARG A  12      115.213  67.905 223.543
ATOM      2  C   ARG A  12      116.106  69.139 223.657
ATOM      3  O   ARG A  12      115.682  70.163 224.199
ATOM      4  H   ARG A  12      116.212  66.040 223.371
ATOM      5  CB  ARG A  12      114.820  67.446 224.960
ATOM      6  CG  ARG A  12      114.005  68.499 225.694
ATOM      7  CD  ARG A  12      113.142  67.891 226.786
ATOM      8  NE  ARG A  12      112.267  68.890 227.417
ATOM      9  CZ  ARG A  12      111.430  68.523 228.411
ATOM     10  NH1 ARG A  12      110.633  69.434 228.986
ATOM     11  NH2 ARG A  12      111.396  67.248 228.822
TER
ENDMDL
MODEL        4
ATOM      0  N   LEU A   1      119.835  51.667 228.178
ATOM      1  CA  LEU A   1      119.177  52.721 228.937
ATOM      2  C   LEU A   1      119.989  54.011 228.861
ATOM      3  O   LEU A   1      119.440  55.091 228.642
ATOM      4  H   LEU A   1      119.418  50.738 228.129
ATOM      5  CB  LEU A   1      119.051  52.287 230.409
ATOM      6  CG  LEU A   1      118.201  51.036 230.563
ATOM      7  CD1 LEU A   1      118.273  50.469 231.971
ATOM      8  CD2 LEU A   1      116.735  51.304 230.263
ATOM      0  N   ARG A   2      121.300  53.900 229.045
ATOM      1  CA  ARG A   2      122.155  55.082 228.980
ATOM      2  C   ARG A   2      122.050  55.721 227.601
ATOM      3  O   ARG A   2      121.915  56.942 227.481
ATOM      4  H   ARG A   2      121.694  53.024 229.223
ATOM      5  CB  ARG A   2      123.616  54.672 229.243
ATOM      6  CG  ARG A   2      124.545  55.874 229.287
ATOM      7  CD  ARG A   2      125.805  55.591 230.089
ATOM      8  NE  ARG A   2      126.648  56.787 230.231
ATOM      9  CZ  ARG A   2      127.809  56.717 230.916
ATOM     10  NH1 ARG A   2      128.577  57.807 231.046
ATOM     11  NH2 ARG A   2      128.193  55.557 231.467
ATOM      0  N   PRO A   3      122.110  54.894 226.559
ATOM      1  CA  PRO A   3      122.025  55.387 225.191
ATOM      2  C   PRO A   3      120.692  56.079 224.890
```

ATOM	3	O	PRO	A	3	120.668	57.149	224.278
ATOM	4	CB	PRO	A	3	122.197	54.154	224.285
ATOM	5	CG	PRO	A	3	122.883	53.118	225.194
ATOM	6	CD	PRO	A	3	122.344	53.459	226.586
ATOM	0	N	VAL	A	4	119.584	55.478	225.322
ATOM	1	CA	VAL	A	4	118.279	56.073	225.052
ATOM	2	C	VAL	A	4	118.272	57.414	225.781
ATOM	3	O	VAL	A	4	117.909	58.442	225.215
ATOM	4	H	VAL	A	4	119.644	54.640	225.820
ATOM	5	CB	VAL	A	4	117.155	55.168	225.589
ATOM	6	CG1	VAL	A	4	115.796	55.846	225.521
ATOM	7	CG2	VAL	A	4	117.047	53.869	224.806
ATOM	0	N	ALA	A	5	118.736	57.442	227.009
ATOM	1	CA	ALA	A	5	118.701	58.697	227.715
ATOM	2	C	ALA	A	5	119.524	59.765	227.052
ATOM	3	O	ALA	A	5	119.117	60.910	226.931
ATOM	4	H	ALA	A	5	119.094	56.637	227.432
ATOM	5	CB	ALA	A	5	119.243	58.476	229.140
ATOM	0	N	ALA	A	6	120.682	59.389	226.539
ATOM	1	CA	ALA	A	6	121.496	60.372	225.801
ATOM	2	C	ALA	A	6	120.760	60.906	224.608
ATOM	3	O	ALA	A	6	120.616	62.099	224.349
ATOM	4	H	ALA	A	6	120.996	58.470	226.648
ATOM	5	CB	ALA	A	6	122.794	59.693	225.324
ATOM	0	N	GLU	A	7	120.186	59.979	223.876
ATOM	1	CA	GLU	A	7	119.475	60.394	222.694
ATOM	2	C	GLU	A	7	118.320	61.306	222.996
ATOM	3	O	GLU	A	7	118.093	62.302	222.328
ATOM	4	H	GLU	A	7	120.239	59.035	224.126
ATOM	5	CB	GLU	A	7	118.937	59.142	221.977
ATOM	6	CG	GLU	A	7	120.057	58.289	221.404
ATOM	7	CD	GLU	A	7	119.530	57.022	220.750
ATOM	8	OE1	GLU	A	7	118.353	56.675	220.929
ATOM	9	OE2	GLU	A	7	120.277	56.336	220.037
ATOM	0	N	VAL	A	8	117.601	61.017	224.067
ATOM	1	CA	VAL	A	8	116.507	61.922	224.464
ATOM	2	C	VAL	A	8	117.032	63.279	224.861
ATOM	3	O	VAL	A	8	116.451	64.288	224.493
ATOM	4	H	VAL	A	8	117.792	60.214	224.590
ATOM	5	CB	VAL	A	8	115.756	61.307	225.660
ATOM	6	CG1	VAL	A	8	114.708	62.255	226.222
ATOM	7	CG2	VAL	A	8	115.039	60.021	225.282
ATOM	0	N	TYR	A	9	118.151	63.345	225.558

```
ATOM      1  CA  TYR A   9     118.714  64.666 225.894
ATOM      2  C   TYR A   9     119.134  65.415 224.654
ATOM      3  O   TYR A   9     118.890  66.607 224.551
ATOM      4  H   TYR A   9     118.603  62.528 225.848
ATOM      5  CB  TYR A   9     119.942  64.474 226.802
ATOM      6  CG  TYR A   9     120.613  65.795 227.141
ATOM      7  CD1 TYR A   9     120.064  66.619 228.088
ATOM      8  CE1 TYR A   9     120.669  67.810 228.393
ATOM      9  CZ  TYR A   9     121.822  68.177 227.750
ATOM     10  CE2 TYR A   9     122.370  67.354 226.803
ATOM     11  CD2 TYR A   9     121.766  66.162 226.498
ATOM     12  OH  TYR A   9     122.430  69.377 228.058
ATOM      0  N   GLN A  10     119.714  64.744 223.675
ATOM      1  CA  GLN A  10     120.068  65.449 222.430
ATOM      2  C   GLN A  10     118.863  65.972 221.638
ATOM      3  O   GLN A  10     118.888  67.099 221.139
ATOM      4  H   GLN A  10     119.907  63.791 223.775
ATOM      5  CB  GLN A  10     120.851  64.477 221.528
ATOM      6  CG  GLN A  10     122.139  64.005 222.184
ATOM      7  CD  GLN A  10     122.887  63.008 221.315
ATOM      8  OE1 GLN A  10     122.399  62.634 220.238
ATOM      9  NE2 GLN A  10     124.076  62.532 221.712
ATOM      0  N   THR A  11     117.809  65.165 221.529
ATOM      1  CA  THR A  11     116.636  65.595 220.774
ATOM      2  C   THR A  11     115.956  66.771 221.540
ATOM      3  O   THR A  11     115.527  67.703 220.856
ATOM      4  H   THR A  11     117.820  64.285 221.954
ATOM      5  CB  THR A  11     115.645  64.424 220.639
ATOM      6  OG1 THR A  11     116.256  63.347 219.924
ATOM      7  CG2 THR A  11     114.384  64.827 219.893
ATOM      0  N   ARG A  12     115.854  66.755 222.868
ATOM      1  CA  ARG A  12     115.193  67.854 223.563
ATOM      2  C   ARG A  12     116.063  69.108 223.612
ATOM      3  O   ARG A  12     115.643  70.133 224.156
ATOM      4  H   ARG A  12     116.222  66.006 223.378
ATOM      5  CB  ARG A  12     114.877  67.414 225.005
ATOM      6  CG  ARG A  12     114.075  68.464 225.758
ATOM      7  CD  ARG A  12     113.277  67.858 226.901
ATOM      8  NE  ARG A  12     112.412  68.851 227.555
ATOM      9  CZ  ARG A  12     111.631  68.486 228.594
ATOM     10  NH1 ARG A  12     110.843  69.391 229.190
ATOM     11  NH2 ARG A  12     111.644  67.218 229.029
TER
```

```
ENDMDL
MODEL     5
ATOM      0  N   LEU A   1     119.626  51.727 228.197
ATOM      1  CA  LEU A   1     119.033  52.809 228.969
ATOM      2  C   LEU A   1     119.890  54.067 228.859
ATOM      3  O   LEU A   1     119.376  55.166 228.650
ATOM      4  H   LEU A   1     119.173  50.814 228.168
ATOM      5  CB  LEU A   1     118.938  52.389 230.447
ATOM      6  CG  LEU A   1     118.046  51.172 230.637
ATOM      7  CD1 LEU A   1     118.142  50.610 232.046
ATOM      8  CD2 LEU A   1     116.583  51.494 230.382
ATOM      0  N   ARG A   2     121.201  53.909 229.001
ATOM      1  CA  ARG A   2     122.097  55.056 228.902
ATOM      2  C   ARG A   2     121.972  55.691 227.522
ATOM      3  O   ARG A   2     121.880  56.916 227.398
ATOM      4  H   ARG A   2     121.568  53.019 229.173
ATOM      5  CB  ARG A   2     123.550  54.593 229.121
ATOM      6  CG  ARG A   2     124.525  55.759 229.127
ATOM      7  CD  ARG A   2     125.798  55.433 229.891
ATOM      8  NE  ARG A   2     126.689  56.597 229.998
ATOM      9  CZ  ARG A   2     127.868  56.487 230.647
ATOM     10  NH1 ARG A   2     128.680  57.548 230.744
ATOM     11  NH2 ARG A   2     128.226  55.317 231.193
ATOM      0  N   PRO A   3     121.968  54.856 226.485
ATOM      1  CA  PRO A   3     121.858  55.345 225.117
ATOM      2  C   PRO A   3     120.544  56.085 224.854
ATOM      3  O   PRO A   3     120.541  57.152 224.235
ATOM      4  CB  PRO A   3     121.955  54.101 224.214
ATOM      5  CG  PRO A   3     122.630  53.045 225.108
ATOM      6  CD  PRO A   3     122.148  53.414 226.514
ATOM      0  N   VAL A   4     119.428  55.529 225.324
ATOM      1  CA  VAL A   4     118.138  56.172 225.092
ATOM      2  C   VAL A   4     118.173  57.480 225.898
ATOM      3  O   VAL A   4     117.676  58.458 225.362
ATOM      4  H   VAL A   4     119.472  54.692 225.825
ATOM      5  CB  VAL A   4     116.991  55.270 225.585
ATOM      6  CG1 VAL A   4     115.652  55.987 225.560
ATOM      7  CG2 VAL A   4     116.845  54.019 224.734
ATOM      0  N   ALA A   5     118.754  57.565 227.081
ATOM      1  CA  ALA A   5     118.808  58.870 227.765
ATOM      2  C   ALA A   5     119.653  59.926 227.042
ATOM      3  O   ALA A   5     119.248  61.087 226.944
ATOM      4  H   ALA A   5     119.145  56.773 227.499
```

ATOM	5	CB	ALA	A	5	119.403	58.657	229.170
ATOM	0	N	ALA	A	6	120.817	59.530	226.530
ATOM	1	CA	ALA	A	6	121.677	60.489	225.843
ATOM	2	C	ALA	A	6	120.975	60.930	224.523
ATOM	3	O	ALA	A	6	121.057	62.124	224.224
ATOM	4	H	ALA	A	6	121.095	58.597	226.612
ATOM	5	CB	ALA	A	6	123.031	59.832	225.515
ATOM	0	N	GLU	A	7	120.321	60.048	223.768
ATOM	1	CA	GLU	A	7	119.691	60.478	222.524
ATOM	2	C	GLU	A	7	118.488	61.365	223.019
ATOM	3	O	GLU	A	7	118.325	62.429	222.424
ATOM	4	H	GLU	A	7	120.262	59.114	224.047
ATOM	5	CB	GLU	A	7	119.187	59.270	221.712
ATOM	6	CG	GLU	A	7	120.333	58.440	221.156
ATOM	7	CD	GLU	A	7	119.837	57.220	220.396
ATOM	8	OE1	GLU	A	7	120.641	56.500	219.786
ATOM	9	OE2	GLU	A	7	118.627	56.946	220.386
ATOM	0	N	VAL	A	8	117.718	60.932	224.007
ATOM	1	CA	VAL	A	8	116.587	61.716	224.473
ATOM	2	C	VAL	A	8	117.115	63.140	224.797
ATOM	3	O	VAL	A	8	116.467	64.082	224.332
ATOM	4	H	VAL	A	8	117.914	60.072	224.429
ATOM	5	CB	VAL	A	8	115.987	61.077	225.739
ATOM	6	CG1	VAL	A	8	116.935	61.163	226.924
ATOM	7	CG2	VAL	A	8	114.689	61.749	226.155
ATOM	0	N	TYR	A	9	118.215	63.298	225.529
ATOM	1	CA	TYR	A	9	118.745	64.610	225.890
ATOM	2	C	TYR	A	9	119.088	65.449	224.653
ATOM	3	O	TYR	A	9	118.746	66.632	224.598
ATOM	4	H	TYR	A	9	118.689	62.502	225.838
ATOM	5	CB	TYR	A	9	120.022	64.419	226.729
ATOM	6	CG	TYR	A	9	120.668	65.746	227.093
ATOM	7	CD1	TYR	A	9	120.142	66.510	228.101
ATOM	8	CE1	TYR	A	9	120.725	67.706	228.429
ATOM	9	CZ	TYR	A	9	121.833	68.137	227.749
ATOM	10	CE2	TYR	A	9	122.359	67.373	226.741
ATOM	11	CD2	TYR	A	9	121.777	66.177	226.413
ATOM	12	OH	TYR	A	9	122.420	69.342	228.079
ATOM	0	N	GLN	A	10	119.753	64.852	223.665
ATOM	1	CA	GLN	A	10	120.125	65.608	222.473
ATOM	2	C	GLN	A	10	118.826	65.978	221.694
ATOM	3	O	GLN	A	10	118.778	67.109	221.204
ATOM	4	H	GLN	A	10	119.992	63.907	223.737

```
ATOM      5  CB  GLN A  10     121.040  64.751 221.578
ATOM      6  CG  GLN A  10     122.354  64.414 222.263
ATOM      7  CD  GLN A  10     123.236  63.528 221.398
ATOM      8  OE1 GLN A  10     122.823  63.125 220.300
ATOM      9  NE2 GLN A  10     124.459  63.183 221.822
ATOM      0  N   THR A  11     117.827  65.105 221.575
ATOM      1  CA  THR A  11     116.630  65.463 220.821
ATOM      2  C   THR A  11     115.873  66.585 221.596
ATOM      3  O   THR A  11     115.384  67.492 220.919
ATOM      4  H   THR A  11     117.895  64.224 221.992
ATOM      5  CB  THR A  11     115.719  64.230 220.674
ATOM      6  OG1 THR A  11     116.401  63.203 219.950
ATOM      7  CG2 THR A  11     114.435  64.557 219.928
ATOM      0  N   ARG A  12     115.769  66.549 222.923
ATOM      1  CA  ARG A  12     115.036  67.597 223.627
ATOM      2  C   ARG A  12     115.822  68.905 223.689
ATOM      3  O   ARG A  12     115.334  69.895 224.241
ATOM      4  H   ARG A  12     116.185  65.823 223.427
ATOM      5  CB  ARG A  12     114.747  67.124 225.064
ATOM      6  CG  ARG A  12     113.877  68.111 225.824
ATOM      7  CD  ARG A  12     113.118  67.444 226.960
ATOM      8  NE  ARG A  12     112.189  68.372 227.621
ATOM      9  CZ  ARG A  12     111.431  67.946 228.654
ATOM     10  NH1 ARG A  12     110.584  68.792 229.257
ATOM     11  NH2 ARG A  12     111.527  66.679 229.078
TER
ENDMDL
```
